# Discovery of a Dysprosium Metallocene Single-Molecule Magnet with Two High-Temperature Orbach Processes

**DOI:** 10.1021/acs.inorgchem.1c03980

**Published:** 2022-04-14

**Authors:** Fu-Sheng Guo, Mian He, Guo-Zhang Huang, Sean R. Giblin, David Billington, Frank W. Heinemann, Ming-Liang Tong, Akseli Mansikkamäki, Richard A. Layfield

**Affiliations:** † Department of Chemistry, School of Life Sciences, University of Sussex, Brighton BN1 9QR, U.K.; ‡ Key Laboratory of Bioinorganic and Synthetic Chemistry of the Ministry of Education, School of Chemistry, Sun-Yat Sen University, Guangzhou 510006, P. R. China; § School of Physics and Astronomy, Cardiff University, Cardiff CF24 3AA, U.K.; ∥ Department of Chemistry and Pharmacy, Inorganic Chemistry, Friedrich-Alexander-University Erlangen-Nürnberg, Egerlandstrabe 1, 91058 Erlangen, Germany; ⊥ NMR Research Group, University of Oulu, P.O. Box 8000, Oulu FI-90014, Finland

## Abstract

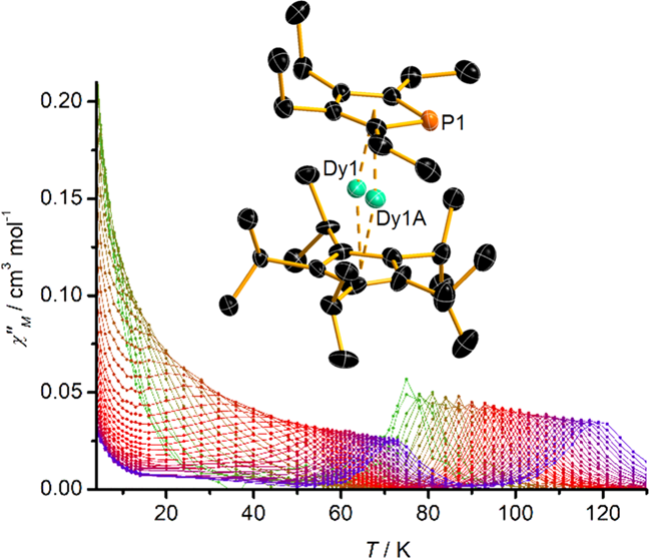

Magnetic bistability in single-molecule magnets (SMMs) is a potential basis for new types of nanoscale information storage material. The standard model for thermally activated relaxation of the magnetization in SMMs is based on the occurrence of a single Orbach process. Here, we show that incorporating a phosphorus atom into the framework of the dysprosium metallocene [(Cp^*i*Pr5^)Dy(Cp^PEt4^)]^+^[B(C_6_F_5_)_4_]^−^ (Cp^*i*Pr5^ is penta-isopropylcyclopentadienyl, Cp^PEt4^ is tetraethylphospholyl) leads to the occurrence of two distinct high-temperature Orbach processes, with energy barriers of 1410(10) and 747(7) cm^–1^, respectively. These barriers provide experimental evidence for two different spin–phonon coupling regimes, which we explain with the aid of ab initio calculations. The strong and highly axial crystal field in this SMM also allows magnetic hysteresis to be observed up to 70 K, using a scan rate of 25 Oe s^–1^. In characterizing this SMM, we show that a conventional Debye model and consideration of rotational contributions to the spin–phonon interaction are insufficient to explain the observed phenomena.

## Abstract 2

A phospholyl-ligated dysprosium metallocene single-molecule magnet shows two high-temperature Orbach relaxation processes with effective energy barriers of 1410(10) and 747(7) cm^−1^, and magnetic hysteresis up to 70 K when using a scan rate of 25 Oe s^−1^.

## Introduction

The magnetic anisotropy within some lanthanide and transition-metal ions forms the basis of the single-molecule magnet (SMM) and single-atom magnet (SAM) effects, in which long magnetization relaxation times can occur in coordination compounds^1−3^ and in single molecules^4,5^ or atoms^6−9^ on surfaces, typically at liquid-helium temperatures. The magnetic hysteresis properties of SMMs are reminiscent of traditional magnetic materials; however, the quantum nature of molecules introduces possibilities for addressing their properties through bespoke modification to the molecular and, hence, electronic structures. Attributes such as these introduce possibilities for the discovery of new physical phenomena, which could lead to the design of novel magnetic materials with applications in data storage and as qubits and spintronic materials.^10−15^ Challenges surrounding the development of SMMs include the need to increase the temperature at which the materials display hysteresis (i.e., the magnetic blocking temperature, *T*_B_) and to maximize the anisotropy barrier (*U*_eff_), which defines the energy required to flip the orientation of the magnetic moment. Many strategies have been employed to address the challenges, such as those based on molecular symmetry,^16−19^ magnetic exchange coupling,^20^ encapsulation of lanthanides within fullerenes,^21^ and, most recently, a dimetallic mixed-valence dysprosium SMM featuring metal–metal bonding that leads to remarkable magnetic hysteresis properties.^22^ Some of us reported the cationic dysprosium metallocene [(Cp^*i*Pr5^)Dy(Cp*)]^+^ (Cp^*i*Pr5^ = penta-isopropylcyclopentadienyl, Cp* = pentamethylcyclopentadienyl), which has a blocking temperature of 80 K and an anisotropy barrier of 1541(11) cm^–1^ (2217(16) K).^23^ The properties of this SMM and related systems originate from the oblate spheroidal 4f^9^ electron density of Dy^3+^ and the strong magnetic anisotropy,^24^ which is enhanced when sandwiching the lanthanide between two cyclopentadienyl ligands to give a strong and highly axial crystal field.^25−32^ At a microscopic level, the dominant relaxation mechanism in dysprosium metallocene SMMs above liquid-helium temperatures is an Orbach-type process consisting of a series of spin–phonon transitions promoted by the vibrational modes of the Cp groups and their substituents.

Our findings on [(Cp^*i*Pr5^)Dy(Cp*)][B(C_6_F_5_)_4_] prompted us to investigate the effects of incorporating atoms other than carbon into the cyclopentadienyl framework of an isostructural dysprosium metallocene SMM. Specifically, our aim was to determine if the third-period element phosphorus would influence the strength and axiality of the crystal field and, hence, the magnetic relaxation. We therefore targeted the phosphorus-containing dysprosium metallocene cation [(Cp^*i*Pr5^)Dy(Cp^PEt4^)]^+^ (**2**) (Cp^PEt4^ is tetraethylphospholyl), which was synthesized as the salt of [B(C_6_F_5_)_4_]^−^ and studied using susceptometry and ab initio calculations.

## Results

### Synthesis

**Figure 1:**
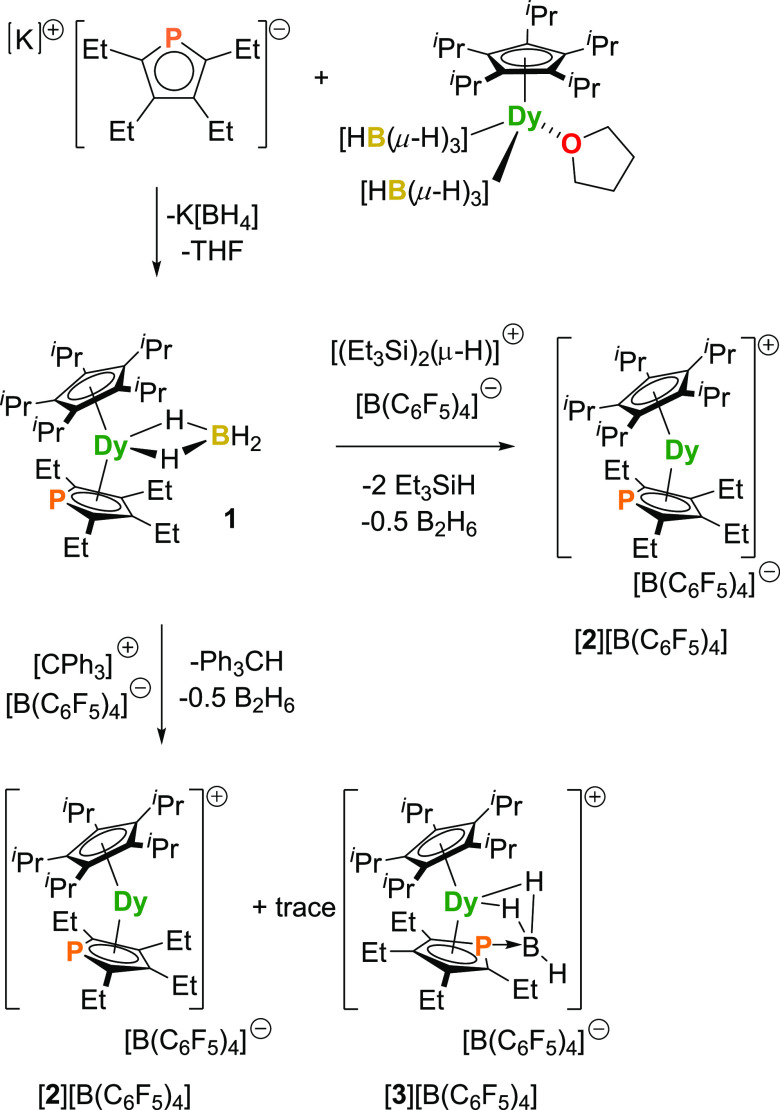
Synthesis of [(Cp*i*Pr5)Dy(Cp^PEt4^)][B(C_6_F_5_)_4_] ([**2**][B(C_6_F_5_)_4_]) by the electrophilic abstraction of a borohydride ligand from [(Cp*i*Pr5)Dy(Cp^PEt4^)(BH_4_)] (**1**). Using [CPh_3_][B(C_6_F_5_)_4_] as the electrophile also leads to the formation of [(Cp*i*Pr5)Dy(C_4_Et_4_PBH_3_)][B(C_6_F_5_)_4_] ([**3**][B(C_6_F_5_)_4_]) as a trace byproduct.

Synthesis of the target compound [**2**][B(C_6_F_5_)_4_] was attempted using two routes, as shown in Figure 1. The phospholyl ligand was installed into the coordination environment of dysprosium through the reaction of [K(Cp^PEt4^)] (Figures S1–S4) with the previously reported half-sandwich compound [(Cp^*i*Pr5^)Dy(BH_4_)_2_(THF)], resulting in the formation of the heteroleptic metallocene [(Cp^*i*Pr5^)Dy(Cp^PEt4^)(BH_4_)] (**1**).^23^ Subsequently, removal of the borohydride ligand was undertaken using the electrophilic reagents [(Et_3_Si)_2_(μ-H)][B(C_6_F_5_)_4_] and [Ph_3_C][B(C_6_F_5_)_4_]. When using the more reactive silylium electrophile, [**2**][B(C_6_F_5_)_4_] was isolated cleanly. In contrast, when the less electrophilic trityl reagent was used, the target compound formed alongside trace amounts (a few crystals) of [(Cp^*i*Pr5^)Dy(C_4_Et_4_PBH_3_)][B(C_6_F_5_)_4_] ([**3**][B(C_6_F_5_)_4_]), in which the nascent BH_3_ byproduct has been captured by the nucleophilic phosphorus atom of the phospholyl ligand.

Mechanical separation of co-crystallized [**2**][B(C_6_F_5_)_4_] and [**3**][B(C_6_F_5_)_4_], obtained from the reaction of [CPh_3_][B(C_6_F_5_)_4_] with **1**, could not reliably be achieved. Therefore, all subsequent analyses were conducted on samples of [**2**][B(C_6_F_5_)_4_] isolated from the reaction of the precursor compound with the silylium reagent, the phase purity of which was established using powder X-ray diffraction (see below). Furthermore, although the FTIR spectrum of **1** shows absorptions in the region of 2038–2455 cm^–1^, corresponding to B–H stretching vibrations, no such signatures were observed in the FTIR spectrum of [**2**][B(C_6_F_5_)_4_] isolated from the reaction of **1** with the silylium reagent (Figures S5 and S6).

### Molecular Structures

**Figure 2:**
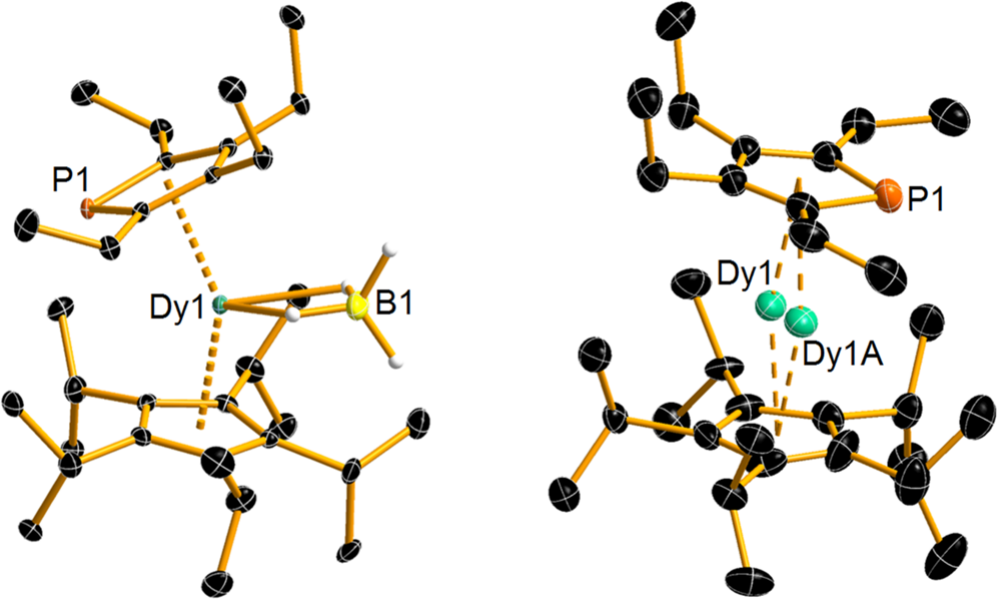
Thermal ellipsoid representation (50% probability) of the structure of **1** (left) and the cation **2** (right) at 100 K. For clarity, only hydrogen atoms bound to boron are shown. Green = Dy, orange = P, black = C, yellow = B.

The molecular structure of [**2**][B(C_6_F_5_)_4_] was determined by single-crystal X-ray diffraction at 100 K using two different diffractometers, using copper and molybdenum Kα radiation, respectively, and at 30 K using Mo Kα radiation (Figures 2, S7, and S8 and Tables S1–S6). The detailed discussion of geometric parameters here focuses on the structure at 100 K collected with Cu Kα radiation, with the other experiments summarized in the Supporting Information. At 100 K, the molecular structure of **2** consists of a Dy^3+^ ion sandwiched between the Cp^*i*Pr5^ and Cp^PEt4^ ligands. The dysprosium atom is disordered across two positions, with occupancies for Dy1 and Dy1A of 72.4 and 27.6%, respectively. In the major disordered component of the structure, the Cp^*i*Pr5^ ligand adopts a distorted η^5^-type interaction with dysprosium, with Dy–C distances in the range of 2.481(8)–2.724(12) Å, whereas the η^5^ interaction with the Cp^PEt4^ ligand produces longer Dy–C distances of 2.618(10)–2.728(9) Å and a Dy–P distance of 2.893(4) Å. The Dy–Cp^*i*Pr5^ and Dy–Cp^PEt4^ distances to the centers of the two ligands are 2.295(7) and 2.366(2) Å, respectively, and the (Cp^*i*Pr5^)-Dy-(Cp^PEt4^) angle is 165.2(1)°.

The structural parameters in the minor disordered component are significantly different from those in the major component. For example, the Dy–C distances to the Cp^*i*Pr5^ and Cp^PEt4^ ligands are 2.344(16)–2.672(11) and 2.657(9)–2.995(16) Å, respectively, and the dysprosium-centroid distances are 2.212(4) and 2.483(5) Å, respectively. The Dy–P distance in the minor component is 2.685(6) Å, i.e., shorter by more than 0.20 Å relative to the major component, and the (Cp^*i*Pr5^)-Dy-(Cp^PEt4^) angle is slightly more bent at 162.0(7)°. At 30 K, only a minor change in the occupancies of the two disordered positions of the dysprosium atom was observed (to 76.4 and 23.6%). Furthermore, the differences in the bond lengths and angles in [**2**][B(C_6_F_5_)_4_] at 30 K (Tables S2–S5) are all within three estimated standard deviations of those at 100 K; hence, no significant structural changes occur. Comparing the structures of **2** and [(Cp^*i*Pr5^)Dy(Cp*)]^+^, the centroids of the ligands in each complex subtend similar bending angles at dysprosium, i.e., 165.2(1)/162.0(7)° and 162.51(1)°, respectively. The Dy–Cp^*i*Pr5^ centroid distances in the two complexes are essentially the same at 2.295(7) and 2.284(1) Å for **2** and [(Cp^*i*Pr5^)Dy(Cp*)]^+^, respectively, but the Dy–Cp^PEt4^ distances of 2.366(2)/2.483(5) Å in **2** are significantly longer than the Dy–Cp* distance of 2.296(1) Å in [(Cp^*i*Pr5^)Dy(Cp*)]^+^. Overall, therefore, these structural parameters provide a qualitative indication that the crystal field experienced by dysprosium in **2** should be highly axial but somewhat weaker than that in [(Cp^*i*Pr5^)Dy(Cp*)]^+^. The origin of this effect is the relatively large phosphorus atom in the phospholyl ligand, which interacts with dysprosium over significantly longer distances than the carbon atoms in the same ligand.^23^ For comparison, at 100 K, the structure of **1** features Dy–Cp^*i*Pr5^ and Dy–Cp^PEt4^ centroid distances of 2.355(2) and 2.415(2) Å, respectively, a Dy–P distance of 2.852 (1) Å and a much more bent (Cp^*i*Pr5^)-Dy-(Cp^PEt4^) angle of 147.69(4)° (Figure 2). The Dy···B distance to the equatorial borohydride ligand is 2.688(4) Å.

The phase purity of [**2**][B(C_6_F_5_)_4_] was established through powder X-ray diffraction experiments using synchrotron radiation at the Diamond Light Source. The powder patterns (Figures S10–S13) were collected at room temperature and 100 K on a crushed polycrystalline sample sealed in a glass capillary. No appreciable changes in the diffraction pattern were observed upon cooling from room temperature, and good matches were found between the experimental 2θ patterns and those calculated using the single-crystal X-ray diffraction data. Furthermore, a comparison of the experimental powder pattern at 100 K with the powder pattern calculated for the byproduct [**3**][B(C_6_F_5_)_4_] confirmed the absence of byproduct from bulk samples of [**2**][B(C_6_F_5_)_4_] (Figure S14), consistent with the FTIR spectrum of this material.

### Magnetic Properties

**Figure 3:**
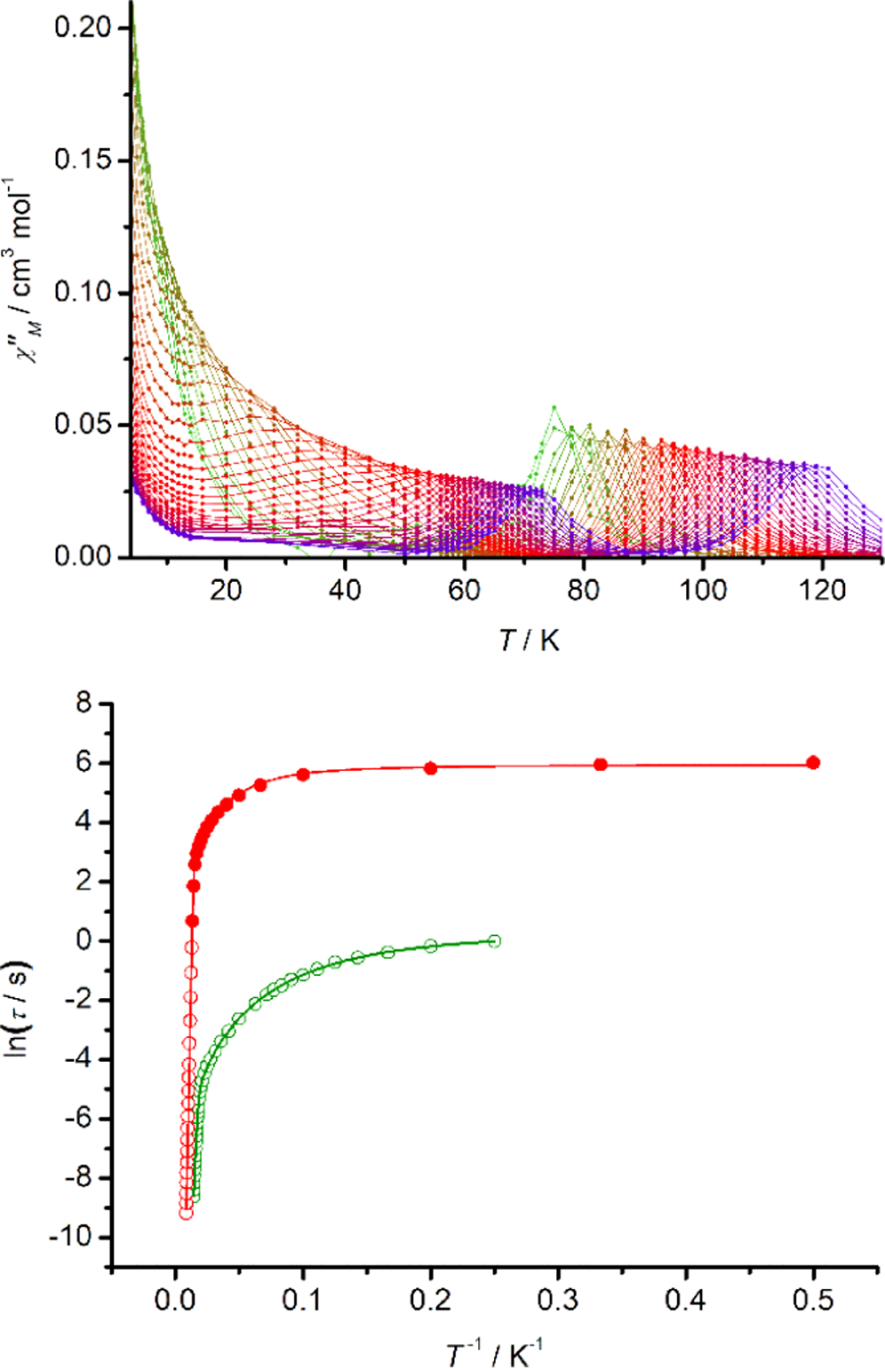
Top: temperature dependence of χ″ at various AC frequencies in the range 0.1–1488 Hz for [**2**][B(C_6_F_5_)_4_]. Solid lines represent a guide for the eye. Bottom: dependence of relaxation time on temperature, plotted as ln (τ/*s*) versus *T*^–1^. Red points correspond to the high-temperature process, with solid points being determined from DC relaxation times. Green points correspond to the lower-temperature process. Solid lines represent fits to the data using the parameters stated in the text.

Having established the phase purity of [**2**][B(C_6_F_5_)_4_], magnetic measurements were then undertaken. The temperature dependence of the DC molar magnetic susceptibility (χ_M_) of [**2**][B(C_6_F_5_)_4_] was determined in a DC field of 1000 Oe (Figure S15). The value of χ_M_*T* at 300 K is 14.25 cm^3^ K mol^–1^, which is close to the theoretical value expected for a single Dy^3+^ ion at this temperature.^33^ A gradual decrease in χ_M_*T* occurs down to ∼35 K before a much more rapid decrease at a lower temperature, indicating the onset of magnetic blocking. At 1.8 K, the value of χ_M_*T* is 3.25 cm^3^ K mol^–1^. The dynamic magnetic properties were then investigated using AC susceptometry. The temperature dependence of the imaginary component of the susceptibility (χ″) was measured at various intervals in the range of 4–130 K and frequencies of ν = 0.1–1488 Hz, using an AC field of 5 Oe and zero DC field (Figures 3 and S17). The χ″(*T*) data consist of a series of well-defined maxima starting at *T* = 119 K and ν = 1488 Hz, with the position of the maximum gradually shifting to *T* = 73 K as the AC frequency is lowered to 0.1 Hz. Below 72 K, a second set of well-defined maxima emerges, with the position of the maximum shifting to 25 K with an AC frequency of 5.34 Hz before the peaks become poorly defined within the frequency window.

The occurrence of two sets of maxima in the χ″(*T*) data at each AC frequency is unusual for a monometallic SMM. The results imply two thermally activated relaxation processes in a system consisting of a single dysprosium ion. Repeated measurement of the AC susceptibility using an independently synthesized batch of [**2**][B(C_6_F_5_)_4_] reproduced the results (Figure S18). Overall, three regions of interest in the χ″(*T*) data can be highlighted, i.e., below 70 K, above 80 K, and the crossover region at 70–80 K, where both peaks are observed within the frequency response of the susceptometer. Considering the individual peak regions, the ratio of the individual isothermal susceptibilities, which was obtained from fits to the real and imaginary parts of the frequency-dependent susceptibility in the high- and low-temperature regions, is ∼3:1. If the two relaxation processes were due to a mixed phase, such an impurity would need to account for ∼35% of the sample, which has been excluded based on powder X-ray diffraction measurements and IR spectroscopy. At 75 K, we attempted to fit the susceptibility data using two interdependent standard Cole–Cole models, where the low-temperature isothermal susceptibility is the adiabatic susceptibility for the high-temperature component. A fit could not be achieved, as confirmed by a fit to the data for two independent Cole–Cole processes (Figures S19 and S20).

Two relaxation processes occurring at such high temperatures are unprecedented. The well-defined nature of the two sets of maxima allows characteristic magnetic relaxation times (τ) to be extracted and modeled using the terms in eq 1
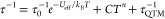
1Orbach relaxation is accounted for by the first term, with *U*_eff_ as the effective energy barrier to reversal of the magnetization and τ_0_ as the attempt time. Raman processes are accounted for by the second term with *C* and *n* the Raman coefficient and Raman exponent, respectively. The rate of quantum tunneling of the magnetization (QTM) is accounted for by the third term, τ_QTM_^–1^ (Figures S21–S29 and Tables S7 and S8).

The dependence of ln *τ* on *T*^–1^ in Figure 3b shows that the two processes are qualitatively similar, being dominated by Orbach relaxation at higher temperatures and QTM below 10 K, with Raman processes accounting for the curvature in the data at intermediate temperatures. Quantitatively, however, the two processes are different since the anisotropy barrier associated with the high-temperature process of *U*_eff_ = 1410(10) cm^–1^ (or 2029(14) K) is almost double the barrier of *U*_eff_ = 747(7) cm^–1^ (or 1075(9) K) for the low-temperature process. The other fitting parameters are *τ*_0_ = 4.77 × 10^–12^ s, *C* = 5.36 × 10^–6^ s^–1^ K^–*n*^, *n* = 2.23, and τ_QTM_ = 381.7 s for the high-temperature process, and *τ*_0_ = 4.23 × 10^–11^ s, *C* = 8.73 × 10^–3^ s^–1^ K^–*n*^, *n* = 2.43, and τ_QTM_ = 1.33 s for the low-temperature process. The small Raman exponents are similar to those found in other SMMs with large energy barriers, particularly dysprosocenium-type SMMs, and consistent with the occurrence of Raman processes at high temperatures.^34,35^ This analysis also reveals that the rates of the two QTM processes differ by more than two orders of magnitude.

### Theoretical Study

To gain insight into the unusual dynamic magnetism of **2**, the electronic structure of both disordered forms of the complex in their crystal structure geometries was studied by ab initio multireference calculations.^36−40^ The energies and principal components of the **g** tensors of the eight lowest Kramers doublets (KDs) corresponding to the crystal-field (CF) split ^6^H_15/2_ ground multiplet are listed for **2a** and **2b** in Table 1.

**Table 1:** Energies and Principal Components of the **g** Tensors of the Eight Lowest Kramers Doublets of **2a** and **2b** Corresponding to the Crystal-Field Split Ground ^6^H_15/2_ Multiplet^a^

	*E* (cm^–1^)	**g**_*x*_	**g**_*y*_	**g**_*z*_	θ (deg)^b^
Cation **2a** (Major Disordered Component of **2**)					
KD1	0	0.00000	0.00000	19.90069	
KD2	537	0.00002	0.00003	16.99708	2.2
KD3	808	0.00417	0.00425	14.41981	3.5
KD4	972	0.02311	0.03172	11.80067	1.4
KD5	1125	0.37495	0.40721	9.10956	5.4
KD6	1281	1.10878	1.69981	6.25320	6.7
KD7	1410	8.35118	7.05939	2.87624	1.1
KD8	1546	0.50594	1.94854	17.54999	89.9
Cation **2b** (Minor Disordered Component of **2**)					
KD1	0	0.00000	0.00000	19.90003	
KD2	543	0.00010	0.00011	16.97607	1.8
KD3	816	0.00120	0.00134	14.38364	4.5
KD4	984	0.02403	0.02705	11.76065	2.6
KD5	1146	0.18290	0.23035	9.10611	4.6
KD6	1313	1.26674	1.64159	6.32628	4.9
KD7	1457	3.25965	4.59251	8.10344	92.3
KD8	1569	0.82290	3.84034	16.43730	89.7

a KD = Kramers doublet.

b Angle between the principal magnetic axis of the doublet and that of the ground doublet.

**Figure 4:**
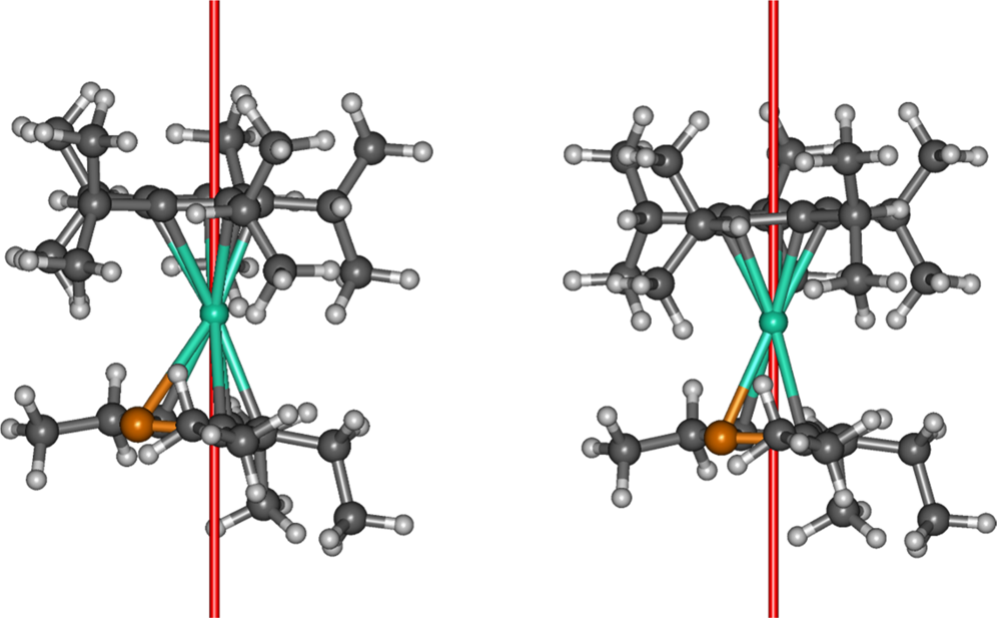
Principal magnetic axis (red line) of the ground Kramers doublet in **2a** (left) and **2b** (right). Green = Dy, orange = P, dark gray = C, light gray = H.

The principal magnetic axes of the ground KD in **2a** and **2b** follow the pseudo-rotational axis of the molecule (Figure 4). The ground KD in both disordered forms of **2** is strongly axial with vanishingly small transverse components in the respective **g** tensors. The transverse components in **2a** increase toward the higher KDs, but all doublets except the highest retain a quasi-axial structure, reflecting the highly axial molecular geometry. A similar pattern is observed for **2b** up to the seventh KD. The ab initio crystal field (CF) parameters were calculated along with the decomposition of the KDs onto angular momentum eigenstates following the CF decomposition (Tables S12 and S13).^41^ The CF splitting is dominated by the very large negative *B*_20_ parameter, which is very similar for **2a** and **2b**. Although the off-diagonal components of the CF make appreciable contributions, they are much smaller than the diagonal components leading to an effectively axial CF. The squared projection of all doublets on some doublet of angular momentum eigenstates with a definite value of angular momentum projection ±*M* is larger than 0.98 in **2a** and **2b** (Tables S14 and S15).

**Figure 5:**
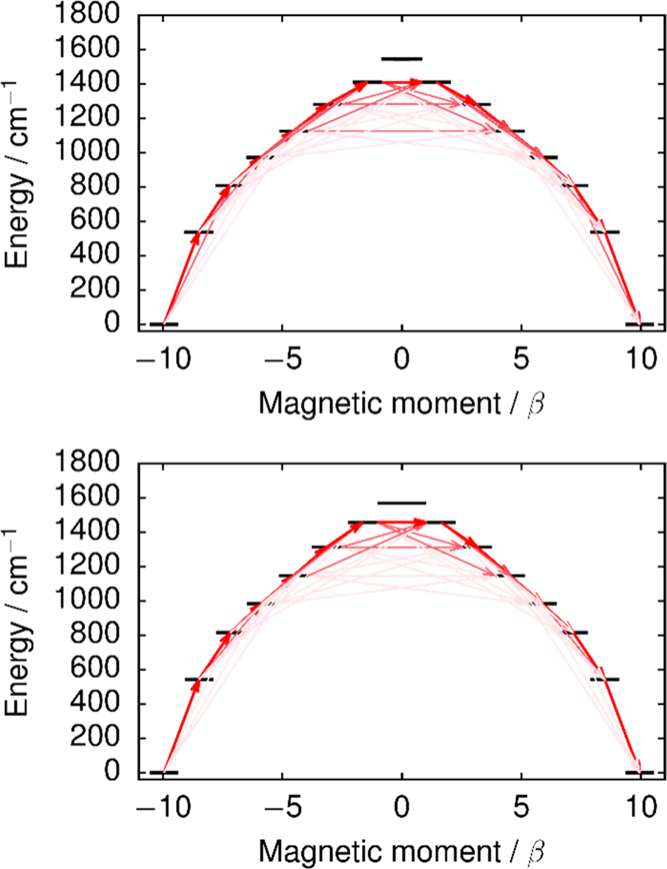
Relaxation barrier in **2a** (top) and **2b** (bottom). Red arrows represent the transition magnetic moments, with stronger color indicating larger values, i.e., more probable transitions.

To explain the two relaxation processes, relaxation barriers for **2a** and **2b** were constructed using an established methodology.^42^ The approach is based on the evaluation of the transition magnetic moments that describes the lowest-rank terms of the electronic part of the spin–phonon transition probability. Assuming that the phonon-dependent terms of the transition probability are roughly constant for each matrix element, the transition probability is proportional to the transition magnetic moment. Such a situation arises, for example, when it is assumed that the relaxation is instigated by acoustic phonon modes, which correspond to rotations of rigid molecules within the crystal lattice, and that the Debye model is valid. In this case, the relaxation process between the various states in the ground manifold can be traced by following the largest transition magnetic moments. The barriers constructed using calculated transition magnetic moments are shown in Figure 5.^43,44^

All transition magnetic moments for barrier-crossing transitions are relatively small even in the higher KDs (Tables S16 and S17). In **2a**, for example, the earliest point at which the barrier can be crossed is KD4; however, KD5–KD7 are more probable crossing points. Since the experimental energy barrier and the energy of KD7 are essentially the same at 1410 cm^–1^, barrier crossing in **2a** most probably takes place via this doublet. However, based on the calculations, crossing via KD5 is also likely. Overall, the close similarities in the electronic structure of the Dy^3+^ ions in **2a** and **2b** make it very unlikely that the two disordered forms of the cation give rise to the two Orbach processes observed in the AC susceptibility.

Although this model explains the higher-barrier process, the lower energy barrier of 747 cm^–1^ is more difficult to rationalize. The value is comparable to the energies of KD2 and KD3 (537 and 808 cm^–1^, respectively), but the transition magnetic moments describing any barrier-crossing process involving these states are vanishingly small. The lower-barrier relaxation process is, therefore, necessarily due to effects other than the rotational contributions to the relaxation—most likely intramolecular vibrations. Thus, we calculated the spin–phonon coupling constants explicitly following a full DFT geometry optimization of **2** (see the Supporting Information for details). The phonon modes were approximated as molecular normal modes, which neglects all effects of acoustic phonons and phonon dispersion. The former approximation has been shown to be reasonable in the relevant energy ranges,^45^ whereas the effect of the latter approximation has not been investigated in detail. The transition rates between states are proportional to the product of the square of the spin–phonon coupling constant and the phonon correlation function, which is different for each vibrational mode. Thus, the barrier cannot simply be constructed by following the largest spin–phonon coupling constants between the different states, which would lead to relaxation via KD7 in this case.

A closer examination of the spin–phonon coupling constants (provided in the Supporting Information) shows that the relaxation via KD7 is instigated with a transition from KD1 and to KD3 on the same side of the barrier (Figure 5). The spin–phonon coupling is mediated by two near-resonant normal modes, and the squared magnitudes of the respective spin–phonon coupling coefficients are 27.2 and 4.9 cm^–1^, respectively. This mechanism is seriously hindered at low temperatures because the phonon modes are extremely weakly occupied. When this mechanism is thermally slowed down, it is possible that another, barrier-crossing Orbach-type mechanism from KD1 on one side of the barrier via KD2 on the other side becomes thermally favorable, as it would require phonons with a lower energy. Such a mechanism would be mediated by two near-resonant vibrational modes. The squared norms of the respective spin–phonon coupling constants are 2.4 × 10^–6^ and 2.5 × 10^–6^ cm^–1^. The values are 7 orders of magnitude smaller than those for the non-barrier-crossing KD1 to KD3 transition. Assuming a harmonic lattice, the ratio of the rates for the barrier-crossing KD1 to KD2 transition and the KD1 to KD3 same-side transition is determined by the ratio of the squared module of the coupling constants and the Bose–Einstein populations of the resonant phonon modes.^46^ With this approximation, the rate for the KD1 to KD2 process would be greater up to a temperature of 27.5 K, suggesting that the lower-barrier process dominates the relaxation up to this temperature and, at higher temperatures, the higher-barrier process dominates. Although this temperature deviates from the 70 K found experimentally, it is within the right order of magnitude.

For completeness, we also calculated the properties of the by-product cation **3**, which also exists in two disordered forms, a major component (**3a**, 87%) and a minor component (**3b**, 13%). The aim of these studies was to support further the idea that the two observed thermal relaxation processes originate solely from within the monometallic SMM **2** and are not due to a mixed-phase impurity, as already shown by powder diffraction measurements and FTIR spectroscopy. The principal magnetic axis in the ground KD in both forms of **3** is roughly aligned with the molecular pseudo-symmetry axis, but with a slight shift away from the phosphorus atom (Figure S58). The principal components of the **g** tensors in the ground KDs of **3a** and **3b** reveal strongly axial character, although somewhat less than found in **2a** and **2b**. However, in the first-excited and higher-lying KDs of **3a** and **3b**, non-negligible transverse **g** tensor components were determined. Furthermore, the principal magnetic axis in the first-excited KDs of **3a** and **3b** are oriented at angles of 12.8 and 7.6° with respect to their ground KDs and, therefore, relaxation is expected to proceed via this route. Since the first-excited KDs lie at 365 and 520 cm^–1^, respectively, above the ground KD, it is extremely unlikely that they account for the lower-energy Orbach process observed in the AC susceptibility measurements on **2**.

### Magnetic Hysteresis

**Figure 6:**
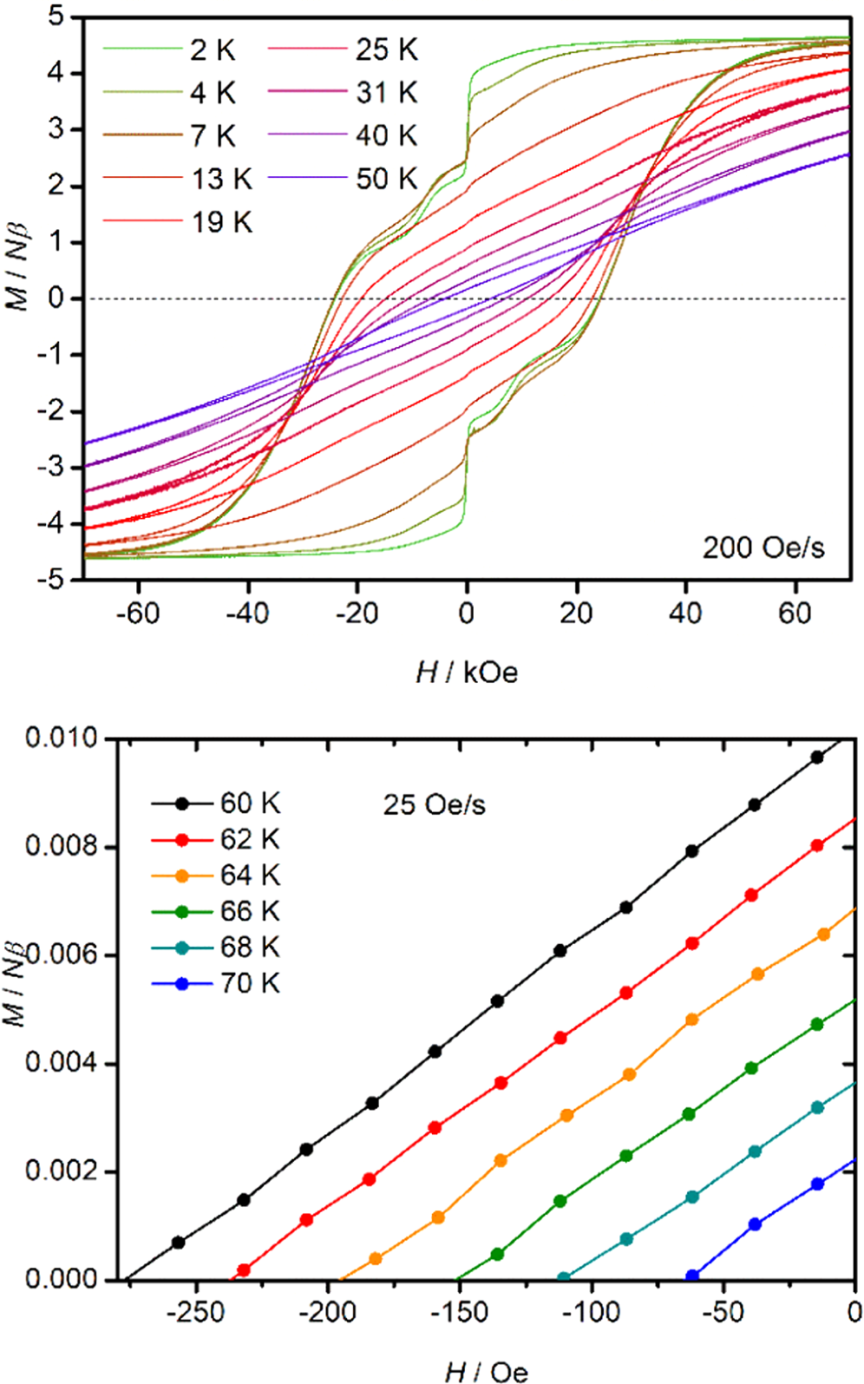
Top: hysteresis loops at various temperatures in the range of 2–50 K using a sweep rate of 200 Oe s^–1^. Bottom: section of the hysteresis loops at low fields and temperatures in the range of 60–70 K, using a sweep rate of 25 Oe s^–1^. Solid lines are a guide to the eye.

Magnetic hysteresis is the key property that underpins the possible applications of SMMs as data storage materials. In the case of [**2**][B(C_6_F_5_)_4_], we first studied the magnetization (*M*) versus field (*H*) hysteresis in the temperature range of 2–75 K using a scan rate of 200 Oe s^–1^ (Figures 6, S47, and S48 and Tables S10 and S11). At 2 K, where both Orbach processes observed in the AC susceptibility do not occur, the magnetization approaches saturation when the magnetic field is increased to 70 kOe. Lowering the field to ∼10 kOe produces only a slight decrease in the magnetization. At lower fields, the magnetization gradually decreases followed by a sharp drop around zero field. Increasing the field in the opposite direction results in two distinct steps centered on 8 and 28 kOe. The coercive field (*H*_c_) under these conditions is 25 kOe. As the temperature of the hysteresis measurement increases, the steps become difficult to discern around 10 K and the loops gradually close as the temperature is increased to 75 K. Reducing the sweep rate to 25 Oe s^–1^ allows the loops to remain open up to 70 K, which defines the blocking temperature [**2**][B(C_6_F_5_)_4_].

Intermolecular dipolar coupling between nearest-neighbor complexes of **2** and intramolecular hyperfine coupling were considered as possible explanations for the steps in the hysteresis at fields of roughly ±8 kOe. For a routine SMM, the dipolar exchange can be investigated by synthesizing the analogous, diamagnetic yttrium compound and preparing a magnetically dilute sample containing 5–10% of the SMM. However, it has not been possible to synthesize the yttrium version of [**2**][B(C_6_F_5_)_4_]. Therefore, we have gained insight using a theoretical approach within the point dipole approximation taking into account the shortest distance between two Dy^3+^ ions in the crystal structure as 10.794 Å. Using the calculated **g** tensors, the energy splitting of the ground doublet resulting from the dipolar coupling corresponds to an equivalent magnetic field of 1.2 kOe. This is considerably weaker than the field at which the steps are observed, making it unlikely that they correspond to any resonance with the dipolar splitting. Furthermore, similar steps were not found in the magnetic hysteresis of [(Cp^*i*Pr5^)Dy(Cp*)]^+^,^23^ which has similar intermolecular Dy···Dy distances. To eliminate hyperfine interactions as the source of the steps in the hysteresis, the isotropic intramolecular hyperfine coupling between the spin system and the nuclear spin of a ^163^Dy nucleus was calculated at the DFT level as 0.011 cm^–1^.^47^ This is a similar order of magnitude to the isotropic coupling constant of 0.03 cm^–1^ determined for a pentagonal bipyramidal Ho^3+^ complex that features several steps in the hysteresis in the range 0–2 kOe.^48^ Considering that the hyperfine coupling in **2** is calculated to be weaker than in the holmium complex, and the steps still take place at a stronger field, it is very unlikely that the hyperfine coupling involving Dy^3+^ is the cause of the steps. Furthermore, the *I* = 5/2 nuclear spin of ^163^Dy should produce several steps in the hysteresis from multiple resonant fields. The hyperfine coupling to the ^31^P nucleus in the [Cp^PEt4^]^−^ ligand was also calculated. The isotropic value is −0.005 cm^–1^, which is much too weak to cause the steps. Based on these results, both dipolar coupling and hyperfine coupling can be ruled out as possible causes for the steps in the magnetic hysteresis of **2**.

## Discussion and Conclusions

The blocking temperature of 70 K and effective energy barriers of 1410(10) and 747(7) cm^–1^ found for **2** are among the highest SMM performance metrics. The dramatic impact of removing the borohydride group from the precursor compound [(Cp^*i*Pr5^)Dy(Cp^Et4P^)(BH_4_)] is reflected in the miniscule energy barrier of *U*_eff_ = 43 cm^–1^ for this compound in an applied DC field of 1500 Oe (Figures S49–S57). Based on a comparison of their molecular structures, the qualitative prediction of the SMM properties of **2** being slightly below those of [(Cp^*i*Pr5^)Dy(Cp*)]^+^ was, generally, observed. A further comparison of the properties of **2** with those reported for a *bis*(phospholyl)-ligated dysprosium SMM is also consistent with this qualitative structure–property relationship. Thus, the narrower bending angle of 157.9(4)° and longer dysprosium-centroid distance of 2.354(3) Å in [(Dtp)_2_Dy][Al{OC(CF_3_)_3_}_4_] (Dtp = {P(C^*t*^BuCMe)_2_}) produce a weaker, less axial crystal and, therefore, a lower energy barrier of 1223 cm^–1^ and a lower blocking temperature of 48 K (sweep rate ∼20 Oe s^–1^).

The theoretical treatment of the relaxation in **2** (in the form of **2a** and **2b**) agrees qualitatively with the experimental results and explains the two thermally activated mechanisms. A more accurate estimate would require more precise values of the spin–phonon coupling constants and a full periodic treatment of the phonon modes with phonon dispersion. Indeed, it has been pointed out that anharmonic effects can play a role already at a qualitative level in SMMs.^49^ The occurrence of two or more Orbach processes in monometallic SMMs has previously been attributed to factors such as ligand-based crystallographic disorder,^50^ dipolar exchange, the effects of external magnetic fields,^51−54^ or they have not been explained.^55^ Ab initio calculations have shown that more than one relaxation mechanism of a purely intramolecular nature can be expected,^56^ and a recent study of a *bis*(phospholyl) dysprosium SMM simulated the Orbach-type relaxation from first principles and also predicted two Orbach relaxation processes.^57^ However, only the higher-barrier process was observed experimentally, and whether the lower-energy barrier and transition temperature between the two relaxation processes were predicted correctly could not be determined. Unlike in **2**, the magnetic hysteresis in most SMMs typically drops sharply around zero field, resulting in narrow loops with small coercive fields. The usual explanation for these properties invokes rapid QTM based on hyperfine interactions and/or the effect of dipolar magnetic fields originating from neighboring spin centers. It has been proposed that such explanations may not be appropriate for SMMs with large anisotropy barriers and that the appearance of the hysteresis loops and the efficiency of the QTM relates to the rigidity of the coordination environment.^58^ If valid, this explanation would account for the hysteresis in **2** since the two ligands are conformationally restricted five-membered rings.

The observation of two Orbach relaxation processes at high temperatures in the monometallic dysprosium SMM **2** demonstrates that, to understand more complicated dynamic behavior in SMMs, it is necessary to go beyond the classical Debye model and to consider the explicit values of the spin–phonon coupling constants. Furthermore, we note that, at a given temperature, subtle effects associated with the molecular geometry can determine the dominant relaxation process, which is extremely difficult to predict a priori. Despite the complex nature of these phenomena, the properties of **2** highlight the switchable nature of SMMs in the high-temperature regime, which may be of use for designing molecule-based magnetic materials.

## Supporting Information Available

General considerations, synthesis details, and NMR spectra (Figures S1–S59 and Tables S1–S19) (PDF)
ESI optimized **2** (XYZ)
Spin–phonon coupling constants (TXT)

## Supplementary Material

ic1c03980_si_001.pdf

ic1c03980_si_002.xyz

## Accession Codes

CCDC 1996560–1996561 and 2096032–2096034 contain the supplementary crystallographic data for this paper. These data can be obtained free of charge via www.ccdc.cam.ac.uk/data_request/cif, or by emailing data_request@ccdc.cam.ac.uk, or by contacting The Cambridge Crystallographic Data Centre, 12 Union Road, Cambridge CB2 1EZ, UK; fax: +44 1223 336033.

## Notes

Additional research data supporting this publication are available as supplementary information at 10.25377/sussex.19519246.
