# Supplementary material for: Discovery of a Dysprosium Metallocene Single-Molecule Magnet with Two High-Temperature Orbach Processes
Source: Inorg Chem. 2022 Apr 14;61(16):6017–25. doi: 10.1021/acs.inorgchem.1c03980 (PMC9044448; doi:10.1021/acs.inorgchem.1c03980)
Supplement: Supplementary file 1 — ic1c03980_si_001.pdf [file ic1c03980_si_001.pdf]

Discovery of a Dysprosium Metallocene Single-Molecule Magnet with Two High-Temperature Orbach Processes

Fu-Sheng Guo,[1] Mian He,[1] Guo-Zhang Huang,[2] Sean R. Giblin,[3] David Billington,[3] Frank W. Heinemann,[4] Ming-Liang Tong,[2,*] Akseli Mansikkamäki,[5,*] and Richard A. Layfield[1,*]

1. Department of Chemistry, School of Life Sciences, University of Sussex, Brighton BN1 9QR, UK
2. Key Laboratory of Bioinorganic and Synthetic Chemistry of the Ministry of Education, School of Chemistry, Sun-Yat Sen University, Guangzhou 510006, P. R. China
3. School of Physics and Astronomy, Cardiff University, Cardiff, CF24 3AA, UK
4. Department of Chemistry and Pharmacy, Inorganic Chemistry, Friedrich-Alexander-University Erlangen-Nürnberg, Egerlandstraße 1, 91058 Erlangen, Germany
5. NMR Research Group, University of Oulu, P.O. Box 8000, Oulu FI-90014, Finland

*Correspondence: tongml@mail.sysu.edu.cn
akseli.mansikkamaki@oulu.fi
r.layfield@sussex.ac.uk

General considerations

All reactions were carried out under rigorous anaerobic, anhydrous conditions and atmospheres of argon or nitrogen. Standard Schlenk and glove-box techniques were used throughout. All solvents were refluxed over an appropriate drying agent for a minimum of three days (molten potassium for toluene and pyridine-D$_5$, Na/K alloy for hexane, CaH_2 for CH_2Cl_2) before distilling, and were stored in ampoules over activated 4 Å molecular sieves. Literature procedures were used to synthesize [(Cp^{iPr5})Dy(BH$_4$)$_2$(THF)],[1] tetraethyl-1-chlorophosphole[2] and [Et$_3$Si(H)SiEt$_3$][B(C$_6$F$_5$)$_4$].[3] Elemental analyses (C, H) were carried out at London Metropolitan University, U.K. IR spectra were collected on a Bruker Alpha FTIR spectrometer fitted with a Platinum ATR module. NMR spectra were acquired on a Varian VNMR S400 spectrometer operating at 400 MHz.

Synthesis of potassium tetraethylphospholide, [K][Cp^{Et4P}]

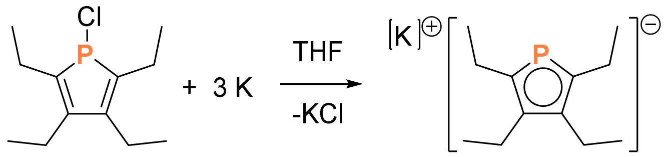

[K][Cp^{Et4P}]

A solution of tetraethyl-1-chlorophosphole (4.02 g, 17.3 mmol) in THF (100 ml) was added to potassium metal (2.00 g, 51.2 mmol) and a glass coated stirrer bar. The mixture was stirred overnight at room temperature. The resulting orange suspension was filtered through a medium frit and the filtrate was evaporated to dryness under a dynamic vacuum, leaving a pale yellow powder. The solid was washed with hexane (4 × 10 ml) and dried, yielding [K][Cp^{Et4P}] as white powder (3.51 g, 86%). ^{1}H NMR spectrum (303 K, pyridine-D$_5$, δ/ppm) 1.33 (t, 6H, 2 × CH$_3$, ^3J = 7.44 Hz), 1.60 (t, 6H, 2 × CH$_3$, ^3J = 7.42 Hz), 2.80 (q, 4H, 2 × CH$_2$, ^3J = 7.44 Hz), 3.11 (m, 4H, 2 × CH$_2$, ^3J = 7.44 Hz). ^{13}C{^{1}H} NMR spectrum (303 K, pyridine-D$_5$, δ/ppm): 18.32 (s, $\underline{C}H_3CH_2C_{Ar}$), 20.52 (d, $\underline{C}H_3CH_2C_{Ar}P$, ^3J = 0.122 Hz), 22.13 (s, $\underline{C}H_2CCP$), 24.99 (d, $\underline{C}H_2CP$, ^2J = 26.51 Hz),131.17 (s, $\underline{C}$–CP), 145.52 (d, $\underline{C}$–P, J = 40.25 Hz). ^{31}P{^{1}H} NMR spectrum (400 MHz, 303 K, pyridine-D$_5$, δ/ppm): 63.6. IR spectrum ($\tilde{\nu}$/cm^{-1}): 2948s, 2924s, 2863s, 1456s, 1389m, 1365m, 1313s, 1255w, 1150m,

1093m, 1049m, 1016w, 935w, 895w, 841w, 812w, 769w, 738w, 634w, 616w, 571w, 545w, 507w, 455w, 416s.

Synthesis of [(Cp^{iPr5})Dy(Cp^{Et4P})(BH$_4$)] (1)

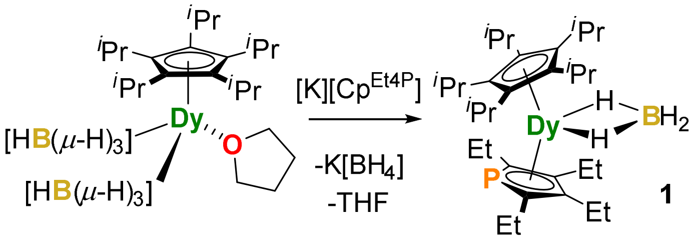

Toluene (15 ml) was added to a solid mixture of [(Cp^{iPr5})Dy(BH$_4$)$_2$THF] (532 mg, 1 mmol), KCp^{Et4} (234 mg, 1 mmol) and a glass coated stirrer bar, and the resulting suspension was stirred with heating at 110°C for two days. The toluene was removed under vacuum, and the product was extracted into hexane (5 × 10 mL) and filtered. Removal of the solvent gave a yellow powder, which was recrystallized from hexane at –40 °C to yield yellow crystals of [(Cp^{iPr5})Dy(Cp^{Et4P})(BH$_4$)] (**1**). Yield = 350 mg, 54 %. Elemental analysis found (calc.) for $C_{32}H_{59}BDyP$ (%): C 59.12 (59.30); H 9.43(9.18). IR spectrum ($\tilde{\upsilon}$/cm^{-1}): 2968s, 2926s, 2870s, 2455m, 2398m, 2121m, 2038w, 1451s, 1366s, 1313m, 1291w, 1214w, 1194w, 1159m, 1122s, 1088s, 1056m, 1027w, 973w, 953w, 900w, 843w, 815w, 767w, 751w, 714w, 609w, 565w, 548m, 508s, 482m, 439s.

Synthesis of [(Cp^{iPr5})Dy(Cp^{Et4P})][B(C$_6$F$_5$)$_4$] ([2][B(C$_6$F$_5$)$_4$])

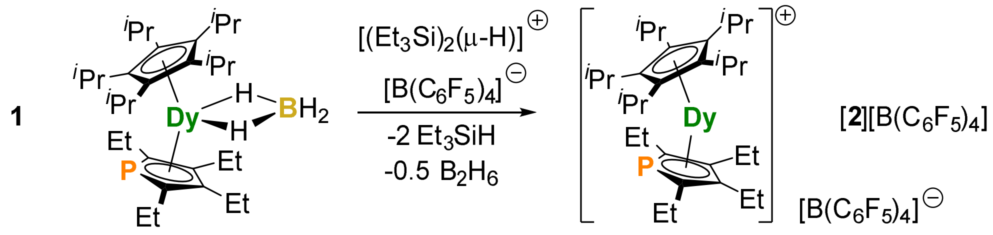

Cold (–40°C) hexane (10 ml) was added into a cold (–40°C) ampoule containing [Et$_3$Si(H)SiEt$_3$][B(C$_6$F$_5$)$_4$] (–40 °C) (180 mg, 0.20 mmol) and a glass coated stirrer bar. A cold solution of [(Cp^{iPr5})Dy(Cp^{Et4P})(BH$_4$)] (**1**) in hexane (–40 °C, 136 mg, 0.21 mmol in 10 ml, 5% excess) was then slowly added. The resulting suspension was sonicated for 50 minutes and stirred for two days to give a yellow suspension. After letting the solid settle, as much of the solution was decanted away and hexane (10ml) was added. This was repeated eight times before drying the solid *in vacuo* to give a yellow powder. Toluene (5 ml) was then added and the suspension was heated to 90°C for 20 minutes to give a yellow solution, which was slowly cooled to room temperature, producing [**2**][B(C$_6$F$_5$)$_4$] as yellow crystals over two days (105 mg, 58 %). Elemental analysis found (calc.) for $C_{56}H_{55}BDyF_{20}P$ (%): C 51.05 (51.25); H 4.29 (4.22). IR spectrum ($\tilde{\upsilon}$/cm^{-1}): 2983w, 2941w, 2878w, 2785w, 1641m, 1510s, 1460s, 1372m, 1312w, 1274m, 1160w, 1082s, 1054w, 979s, 907w, 775s, 756s, 726w, 683s, 660s, 609w, 572m, 512m, 478w, 431w.

Alternative synthesis leading to a mixture of [2][B(C$_6$F$_5$)$_4$] and [3][B(C$_6$F$_5$)$_4$]. [Ph$_3$C][B(C$_6$F$_5$)$_4$] (142 mg, 0.15 mmol) was added to a solution of [(Cp^{iPr5})Dy(Cp^{Et4P})(BH$_4$)] (**1**) (100 mg, 0.15 mmol) in cold hexane (–40 °C, 10mL) with a glass-coated stirrer bar. The reaction was warmed to room temperature. The resulting suspension was sonicated for 50 minutes and then stirred for 48 hours, which produced a yellow powder. After letting the solid settle, the hexane was decanted away and the residue washed with hexane (5 × 10 mL). The resulting yellow powder was dried under vacuum. Toluene (5 ml) was added and the suspension was heated to 90°C to give a yellow solution with an orange oil. Cooling to room temperature and storing for two days produced bright yellow crystals. The solvent was decanted away and the crystals were washed with cold toluene (3 × 5 mL) and hexane (3 × 5 mL), giving a mixture of crystals of [**2**][B(C$_6$F$_5$)$_4$] and [**3**][B(C$_6$F$_5$)$_4$] as the major and minor product, respectively.

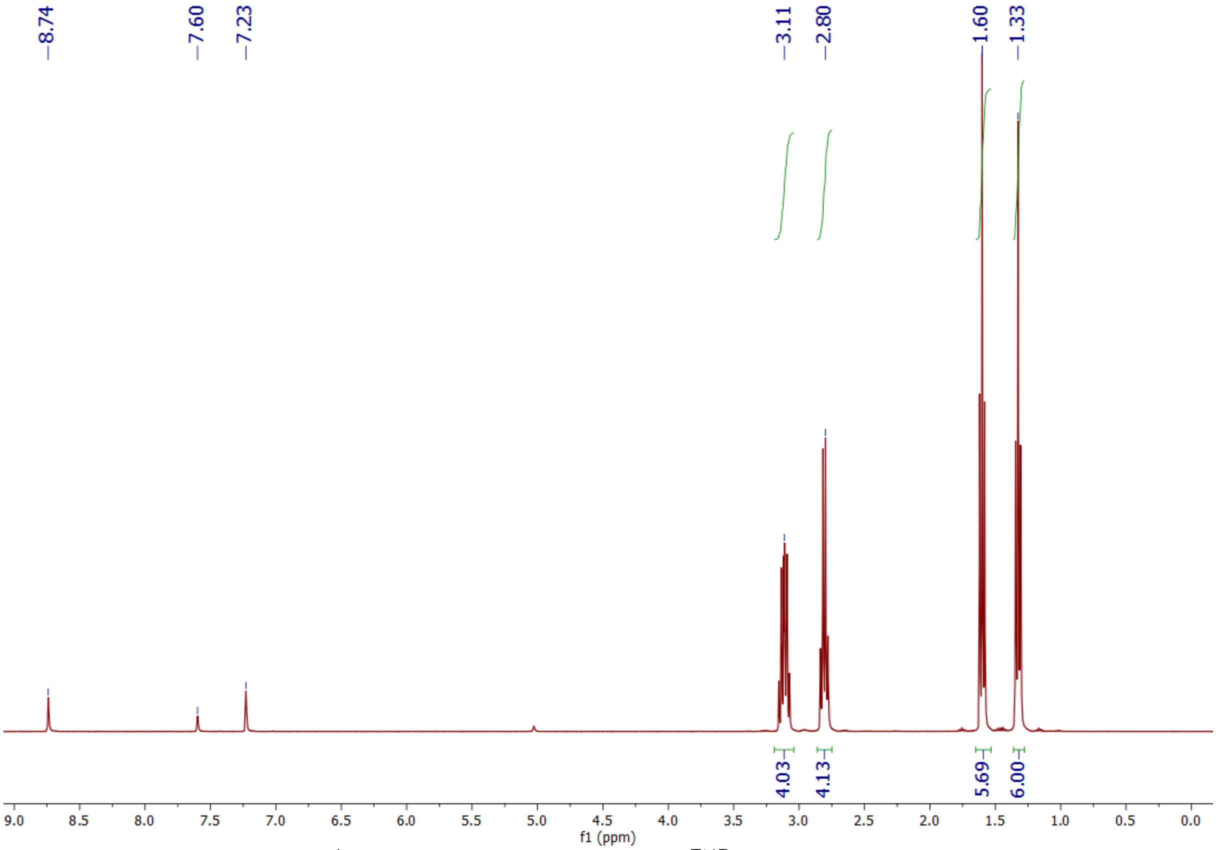

Figure S1. ^{1}H NMR spectrum of [K][Cp^{Et4P}] in pyridine-D$_5$ at 30°C.

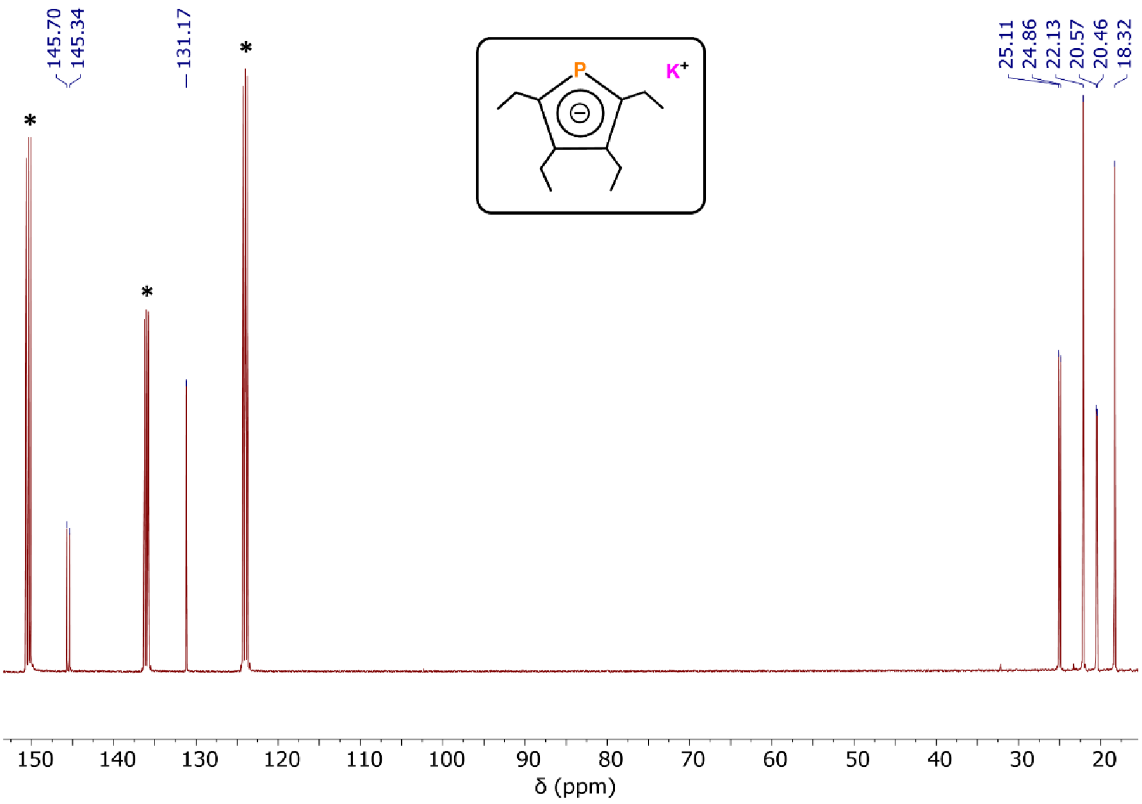

Figure S2. ^{13}C NMR spectrum of [K][Cp^{Et4P}] in pyridine-D_5 at 303 K. Black asterisks correspond to residual solvent peaks.

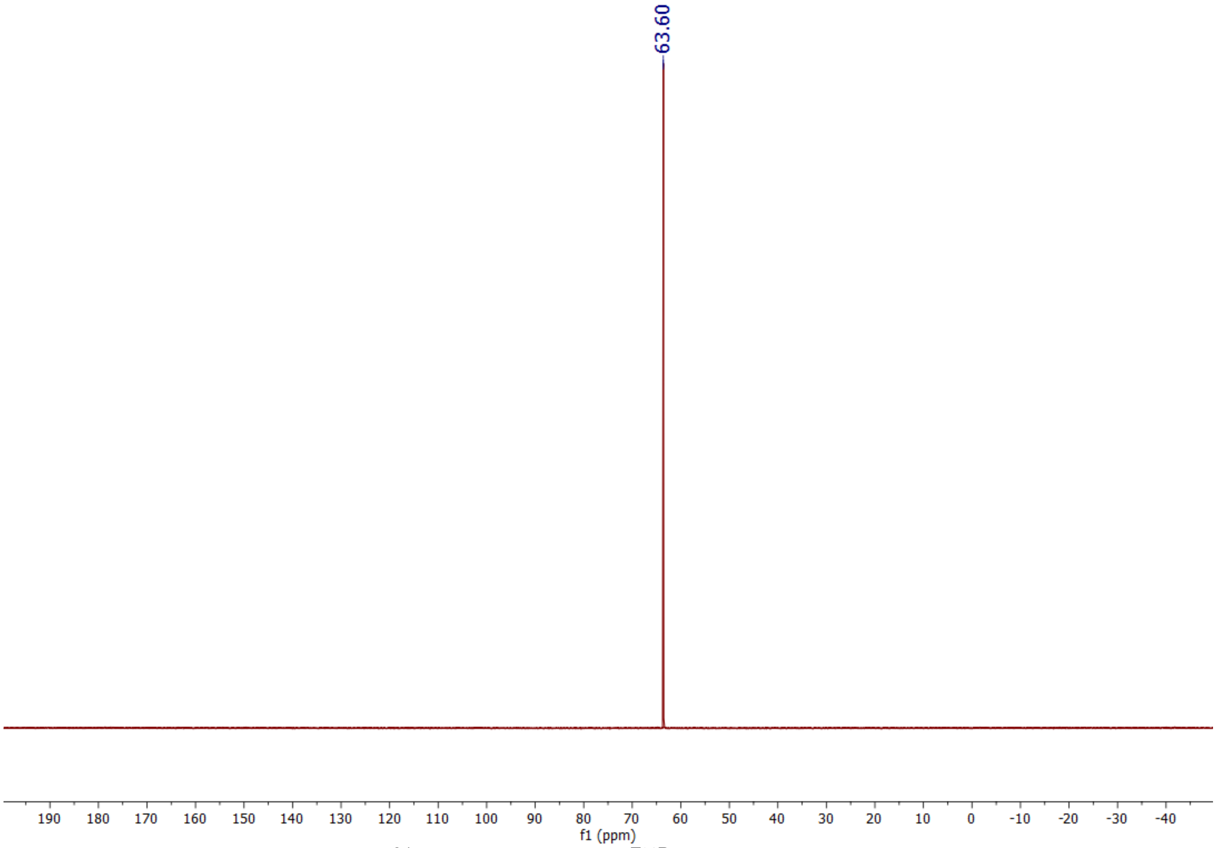

Figure S3. ^{31}P NMR of [K][Cp^{Et4P}] in pyridine-D$_5$ at 30°C.

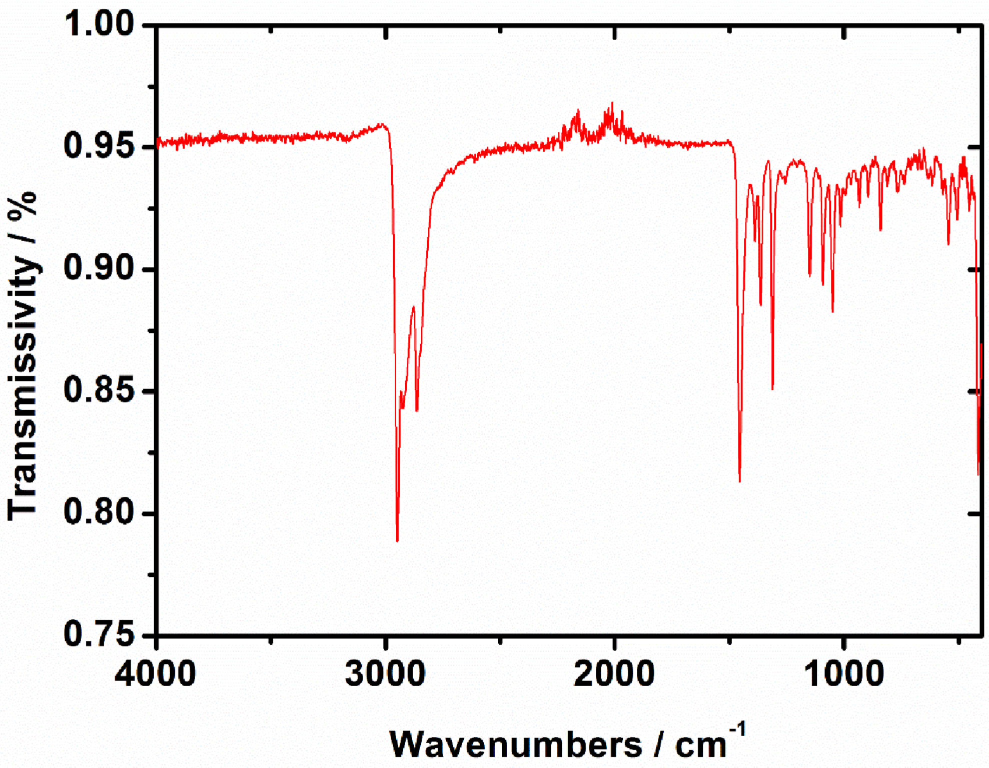

Figure S4. Infrared spectrum of [K][Cp^{Et4P}].

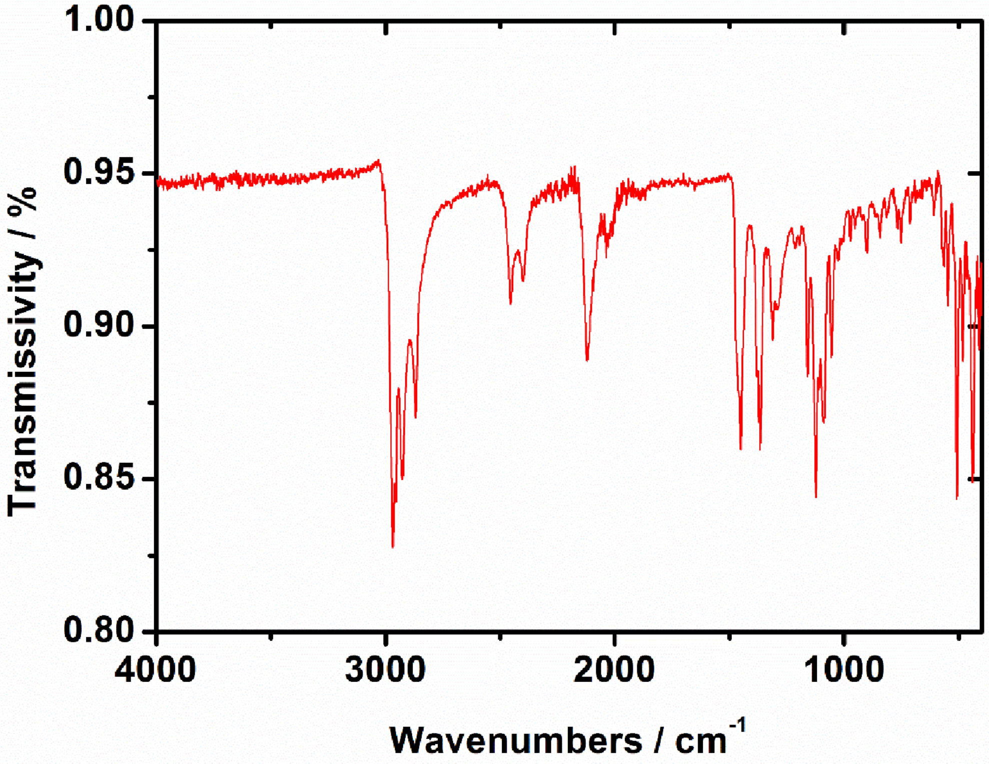

Figure S5. Infrared spectrum of [(Cp^{iPr5})Dy(Cp^{Et4P})(BH_4)] (**1**).

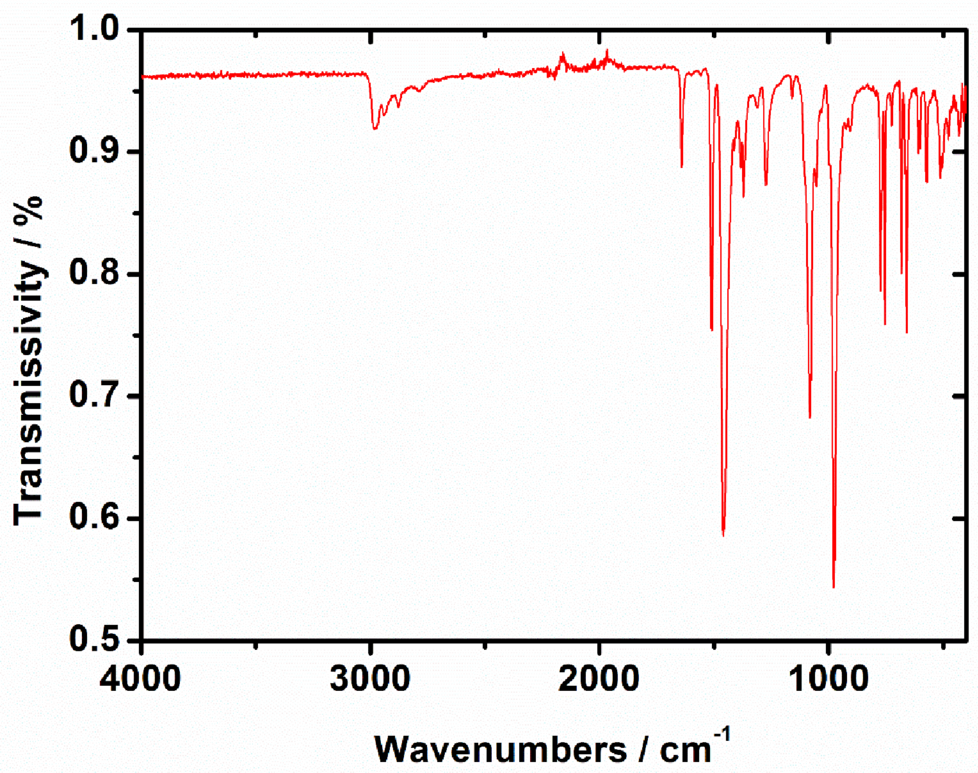

Figure S6. Infrared spectrum of [**2**][B(C_6F_5)$_4$].

X-ray crystallography

X-ray diffraction data for [(Cp^{iPr5})Dy(Cp^{Et4P})(BH_4)] (**1**) and [**3**][B(C_6F_5)$_4$] were collected on an Agilent Gemini Ultra diffractometer at the University of Sussex. Data for [**2**][B(C_6F_5)$_4$] were collected using a Rigaku microfocus rotating anode diffractometer at the University of Sussex using Cu-Kα radiation. Data for [**2**][B(C_6F_5)$_4$] at 100 K and 30 K were also collected on a Bruker Smart APEX II diffractometer, using Mo-Kα radiation. Structures were solved in Olex2 with SHELXT using intrinsic phasing and were refined with SHELXL using least squares minimisation.[4–6] Anisotropic thermal parameters were used for the non-hydrogen atoms and isotropic parameters for the hydrogen atoms. Hydrogen atoms on carbons were added geometrically and refined using a riding model. Hydrogen atoms for BH_4 fragments in compound **1** were located from the Q peaks around the boron atoms and isotropically refined. Structures were deposited at the Cambridge Structural Database and assigned the references codes shown in Table S1.

Table S1. Crystal data and structure refinement for **1**, [**2**][B(C_6F_5)$_4$] (on two instruments at 100 K, and at 30 K on one) and [**3**][B(C_6F_5)$_4$].

	**1**	**[2][B(C_6F_5)$_4$]^c**	**[2][B(C_6F_5)$_4$]^d**	**[2][B(C_6F_5)$_4$]^d**	**[3][B(C_6F_5)$_4$]**
CSD ref. code	1996560	1996561	2096032	2096033	2096034
Formula	$C_{32}H_{59}BDyP$	$C_{56}H_{55}BDyF_{20}P$	$C_{56}H_{55}BDyF_{20}P$	$C_{56}H_{55}BDyF_{20}P$	$C_{56}H_{58}B_2DyF_{20}P$
FW	648.07	1312.28	1312.28	1312.28	1326.11
T/K	100	100	100	30	100
Crystal system	Triclinic	Orthorhombic	Orthorhombic	Orthorhombic	Monoclinic
Space group	P–1	$Pna2_1$	$Pna2_1$	$Pna2_1$	$P2_1/c$
a/Å	9.8357(4)	17.8192(4)	17.6552(3)	17.5963(4)	16.7542(2)
b/Å	11.2310(5)	16.9757(5)	17.0120(2)	16.9862(4)	14.7179(2)
c/Å	16.6786(6)	17.5726(4)	17.4808(3)	17.4311(4)	22.3108(3)
α/°	70.974(4)	90	90	90	90
β/°	87.088(3)	90	90	90	97.1450(10)
γ/°	67.371(4)	90	90	90	90
V/Å^3	1601.77(13)	5315.6(2)	5250.36(14)	5210.1(2)	5458.82(12)
Z	2	4	4	4	4
ρ_{calc}/g·cm^{-3}	1.344	1.640	1.660	1.673	1.614
μ/mm^{-1}	13.053	8.778	1.564	1.576	8.551
Crystal size/mm^3	0.12 × 0.1 × 0.03	0.08 × 0.07 × 0.05	0.5 × 0.1 × 0.05	0.5 × 0.1 × 0.05	0.1 × 0.05 × 0.05
2θ range/°	8.924 to 134.154	7.192 to 134.14	4.06 to 57.394	4.07 to 55.754	7.212 to 142.438
Reflections collected	10946	47958	112637	150038	18695
Independent reflections	5711	9379	13221	12378	10342
R_{int}	0.0297	0.0812	0.0875	0.0955	0.0300
R_{sigma}	0.0417	0.0540	0.0937	0.0627	0.0450
Final $R_1{}^a$ values (I > 2σ(I))	0.0314	0.0561	0.0584	0.0592	0.0479
Final $wR_2{}^b$(F^2) values (I > 2σ(I))	0.0802	0.1422	0.1259	0.1449	0.1229
Final $R_1{}^a$ values (all data)	0.0325	0.0657	0.0970	0.0747	0.0549
Final $wR_2{}^b$(F^2) values (all data)	0.0811	0.1560	0.1380	0.1516	0.1289
Completeness/%	99.8	99.7	99.8	99.9	99.8
Data/restraints/parameters	5711/0/334	9379/41/781	13221/900/769	12378/978/769	10342/0/757
Goodness-of-fit on F^2	1.037	1.032	1.026	1.038	1.039

^{a} $R_1 = \sum||F_o|-|F_c||/\sum|F_o|$, ^{b} $wR_2 = [\sum w(F_o{}^2-F_c{}^2)^2/\sum w(F_o{}^2)^2]^{1/2}$, ^{c} Rigaku diffractometer, ^{d} Bruker diffractometer.

Table S2. Selected bond lengths (Å) and angles (°) for **1**.

Dy–C (Cp^{iPr5})	Dy1–C1: 2.653(3)<br>Dy1–C2: 2.628(3)<br>Dy1–C3: 2.638(3)<br>Dy1–C4: 2.651(3)<br>Dy1–C5: 2.683(3)
Dy–C (Cp^{Et4P})	Dy1–C21: 2.693(3)<br>Dy1–C22: 2.746(3)<br>Dy1–C23: 2.738(3)<br>Dy1–C24: 2.741(3)
Dy–P	2.8526(7)
Dy···B	2.688(4)
Dy–centroid (Cp^{iPr5})	2.355(2)
Dy–centroid (Cp^{Et4P})	2.415(2)
Cp_c–Dy–Cp_c	147.69(4)

Table S3. Selected bond lengths (Å) and angles (°) at 100 K for [**2**][$B(C_6F_5)_4$] collected using a Rigaku microfocus rotating anode diffractometer.

	Disordered part 1 (72.4 %)	Disordered part 2 (27.6 %)
Dy–C (Cp^{ipr5})	Dy1–C1: 2.724(12)<br>Dy1–C2: 2.630(10)<br>Dy1–C3: 2.481(8)<br>Dy1–C4: 2.508(8)<br>Dy1–C5: 2.666(10)	Dy1A–C1: 2.344(16)<br>Dy1A–C2: 2.415(11)<br>Dy1A–C3: 2.603(11)<br>Dy1A–C4: 2.672(11)<br>Dy1A–C5: 2.531(10)
Dy–C (Cp^{Et4P})	Dy1–C21: 2.659(9)<br>Dy1–C22: 2.618(10)<br>Dy1–C23: 2.649(9)<br>Dy1–C24: 2.728(9)	Dy1A–C21: 2.824(12)<br>Dy1A–C22: 2.995(16)<br>Dy1A–C23: 2.897(13)<br>Dy1A–C24: 2.657(9)
Dy–P	Dy1–P1: 2.893(4)	Dy1A–P1: 2.685(6)
Dy–centroid (Cp^{ipr5})	Dy1–Cp: 2.295(7)	Dy1A–Cp: 2.212(4)
Dy–centroid (Cp^{Et4P})	Dy1–Cp: 2.366(2)	Dy1A–Cp: 2.483(5)
Cp_c–Dy–Cp_c	Dy1: 165.2(1)	Dy1A: 162.0(7)

Table S4. Selected bond lengths (Å) and angles (°) at 100 K for [**2**][$B(C_6F_5)_4$] collected using a Bruker Smart APEX II diffractometer.[a]

	Disordered part 1 (74.7 %)	Disordered part 2 (25.3 %)
Dy–C (Cp^{iPr5})	Dy1–C1: 2.699(12)<br>Dy1–C2: 2.636(12)<br>Dy1–C3: 2.489(11)<br>Dy1–C4: 2.523(10)<br>Dy1–C5: 2.648(11)	Dy1A–C1: 2.370(13)<br>Dy1A–C2: 2.423(12)<br>Dy1A–C3: 2.571(13)<br>Dy1A–C4: 2.665(11)<br>Dy1A–C5: 2.537(12)
Dy–C (Cp^{Et4P})	Dy1–C21: 2.670(10)<br>Dy1–C22: 2.632(11)<br>Dy1–C23: 2.687(9)<br>Dy1–C24: 2.702(10)	Dy1A–C21: 2.819(11)<br>Dy1A–C22: 2.949(12)<br>Dy1A–C23: 2.875(10)<br>Dy1A–C24: 2.614(10)
Dy–P	Dy1–P1: 2.881(3)	Dy1A–P1: 2.697(6)
Dy–centroid (Cp^{iPr5})	Dy1–Cp: 2.305(1)	Dy1A–Cp: 2.209(1)
Dy–centroid (Cp^{Et4P})	Dy1–Cp: 2.369(1)	Dy1A–Cp: 2.458(1)
Cp_c–Dy–Cp_c	Dy1: 164.8(2)	Dy1A: 166.2(2)

[a] The atom labelling used in this table has been adapted to the numbering used in Table S3 and is different to the atom labelling scheme in the corresponding CIF.

Table S5. Selected bond lengths (Å) and angles (°) at 30 K for [**2**][$B(C_6F_5)_4$] collected using a Bruker Smart APEX II diffractometer.[a]

	Disordered part 1 (76.4 %)	Disordered part 2 (23.6 %)
Dy–C (Cp^{iPr5})	Dy1–C1: 2.687(13)	Dy1A–C1: 2.347(14)
	Dy1–C2: 2.637(13)	Dy1A–C2: 2.409(14)
	Dy1–C3: 2.508(12)	Dy1A–C3: 2.584(13)
	Dy1–C4: 2.514(11)	Dy1A–C4: 2.662(13)
	Dy1–C5: 2.651(12))	Dy1A–C5: 2.546(12)
Dy–C (Cp^{Et4P})	Dy1–C21: 2.671(11)	Dy1A–C21: 2.830(12)
	Dy1–C22: 2.641(11)	Dy1A–C22: 2.971(12)
	Dy1–C23: 2.689(11)	Dy1A–C23: 2.876(12)
	Dy1–C24: 2.709(11)	Dy1A–C24: 2.619(11)
Dy–P	Dy1–P1: 2.881(3)	Dy1A–P1: 2.695(6)
Dy–centroid (Cp^{iPr5})	Dy1–Cp_c^{iPr5}: 2.305(1)	Dy1A–Cp_c^{iPr5}: 2.205(1)
Dy–centroid (Cp^{Et4P})	Dy1–Cp_c^{Et4P}: 2.372(1)	Dy1A–Cp_c^{Et4P}: 2.466(1)
Cp_c–Dy–Cp_c	Dy1: 164.5(2)	Dy1A: 165.7(2)

[a] The atom labelling used in this table has been adapted to the numbering used in Table S3 and is different to the atom labelling scheme in the corresponding CIF.

Table S6. Selected bond lengths (Å) and angles (°) for [**3**][B(C_6F_5)$_4$].

	Disordered part 1 (87 %)	Disordered part 2 (13 %)
Dy–C (Cp^{iPr5})	Dy1–C1: 2.578(4)<br>Dy1–C2: 2.665(4)<br>Dy1–C3: 2.691(4)<br>Dy1–C4: 2.618(5)<br>Dy1–C5: 2.559(4)	Dy1A–C1: 2.718(5)<br>Dy1A–C2: 2.761(5)<br>Dy1A–C3: 2.377(5)<br>Dy1A–C4: 2.034(6)<br>Dy1A–C5: 2.304(5)
Dy–C (Cp^{Et4P})	Dy1–C21: 2.728(4)<br>Dy1–C22: 2.874(4)<br>Dy1–C23: 2.850(4)<br>Dy1–C24: 2.669(4)	Dy1A–C21: 2.976(5)<br>Dy1A–C22: 2.761(5)<br>Dy1A–C23: 2.739(5)<br>Dy1A–C24: 2.929(5)
Dy–P	Dy1–P1: 2.8406(11)	Dy1A–P1: 3.4049(1)
Dy–centroid (Cp^{iPr5})	Dy1-Cp$_c$: 2.3253(1)	Dy1-Cp$_c$: 2.1328(1)
Dy–centroid (Cp^{Et4P})	Dy1-Cp$_c$: 2.4581(1)	Dy1A-Cp$_c$: 2.6584(1)
P–B	P1-B1: 2.016(6)	P1-B1: 2.016(6)
Dy···B	Dy1–B1: 2.555(5)	Dy1A–B1: 3.3781(1)
Cp$_c$–Dy–Cp$_c$	Dy1: 159.0710(1)	Dy1A: 157.952(1)

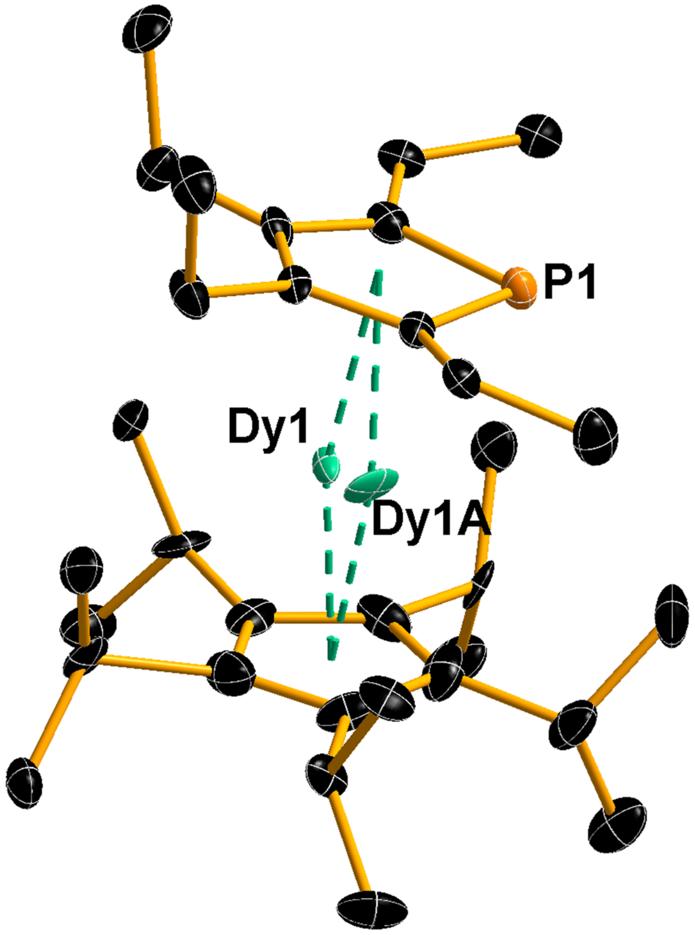

Figure S7. Molecular structure of the cation 2. Collected on a Bruker Smart APEX II diffractometer at 100 K. Thermal ellipsoid representation (50% probability). Green = dysprosium, orange = phosphorus, black = carbon. Hydrogen atoms are omitted for clarity.

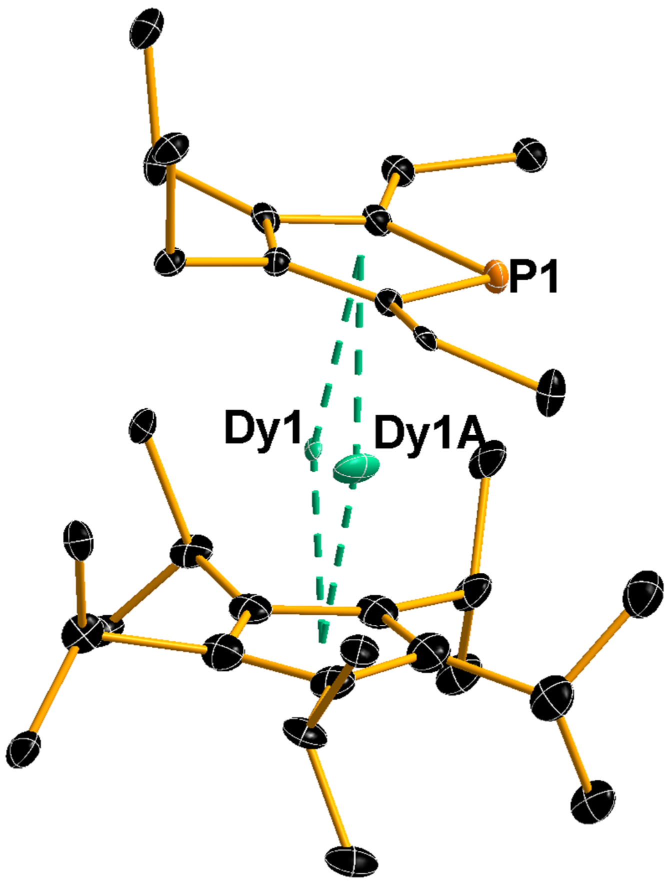

Figure S8. Molecular structure of the cation 2. Collected on a Bruker Smart APEX II diffractometer at 30 K. Thermal ellipsoid representation (50% probability). Green = dysprosium, orange = phosphorus, black = carbon. Hydrogen atoms are omitted for clarity.

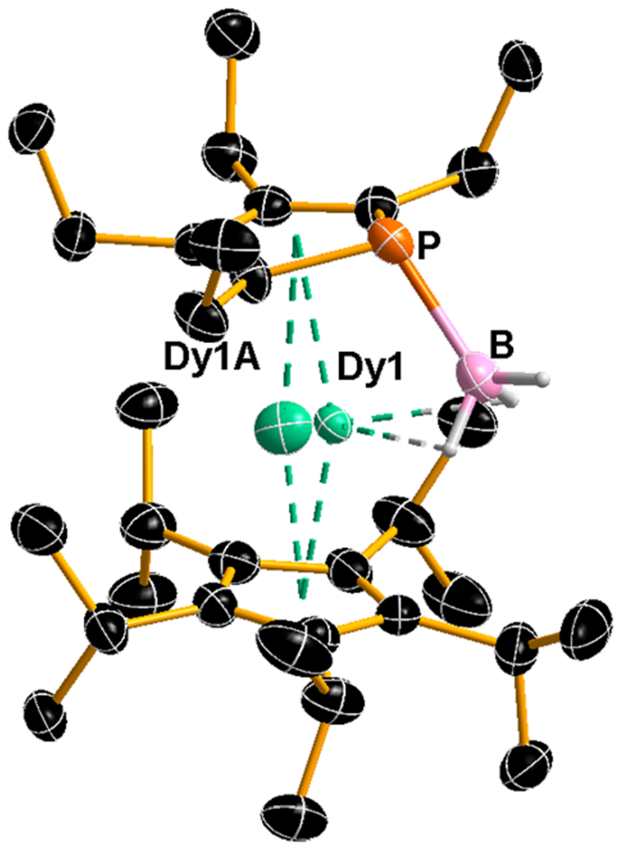

Figure S9. Thermal ellipsoid representation (50% probability) of the structure of the cation **3**. Green = dysprosium, orange = phosphorus, black = carbon, pink = boron, white = hydrogen (only non-C–H hydrogen atoms are shown).

Powder X-ray Diffraction

Powder XRD data were collected using synchrotron radiation at the Diamond Light Source, beamline I11, at room temperature and 100 K. All experiments were conducted by Dr Sarah Day. The sample took the form of crushed polycrystalline material and was measured in a glass capillary. A beam current of 300 mA was used and data files were obtained using the MAC detection system. Instrument calibrations were performed using Si powder standard (NIST SRM640c). The wavelength was λ = 0.826838(5) Å and the 2θ zero-point was 0.00159°.

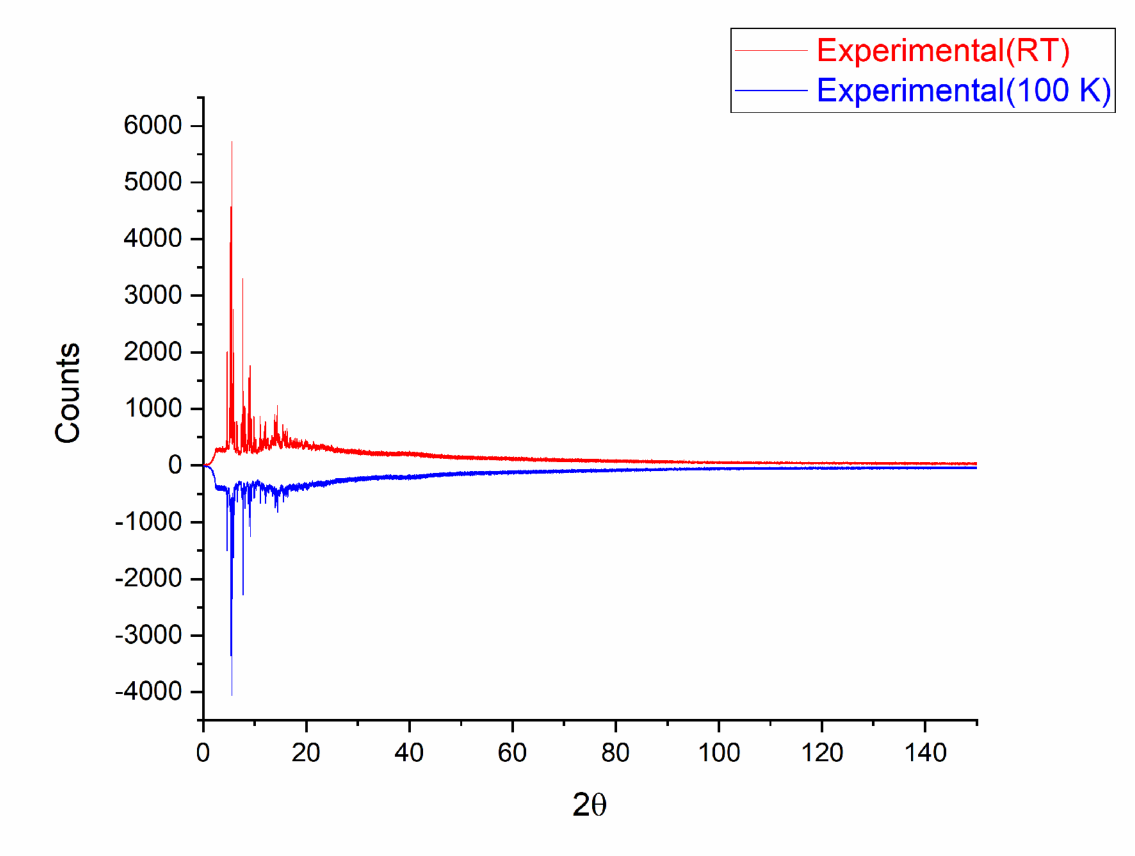

Figure S10. Powder XRD pattern of [**2**][$B(C_6F_5)_4$] at room temperature (red line) and at 100 K (blue line).

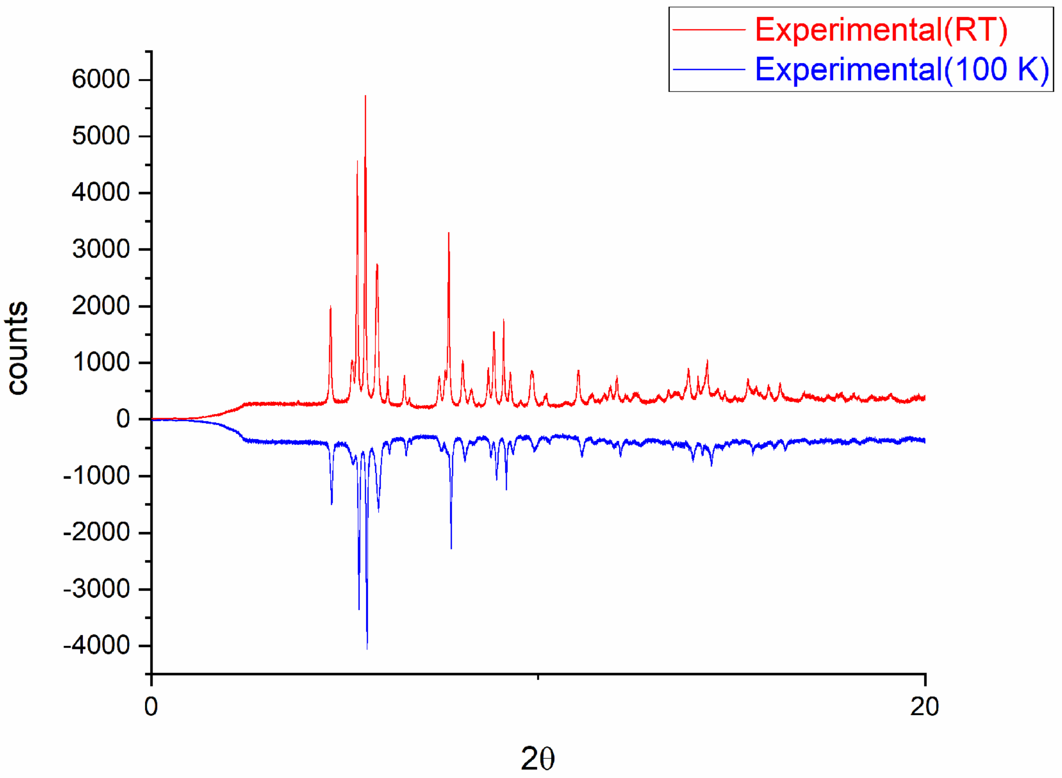

Figure S11. Powder XRD pattern of [**2**][B(C_6F_5)$_4$] at room temperature (red line) and at 100 K (blue line) up to 2θ = 20°.

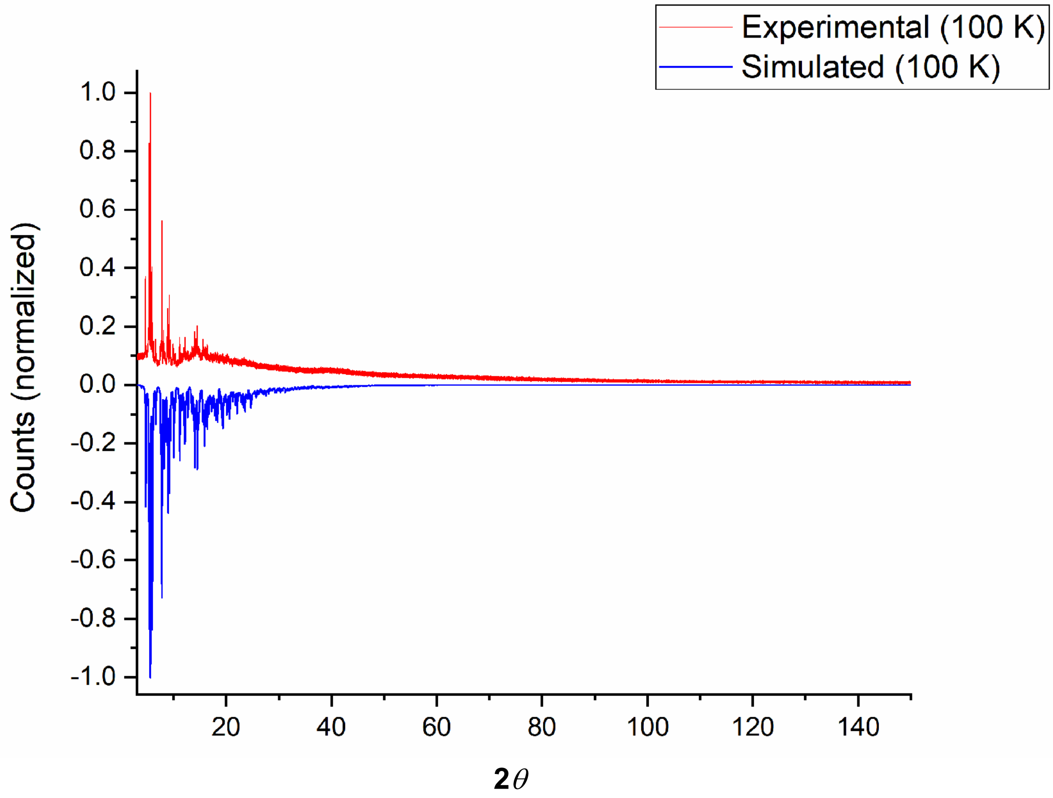

Figure S12. Experimental powder XRD pattern of [**2**][B(C_6F_5)$_4$] at 100 K (red line) compared to the calculated pattern based on the CIF file obtained from a single crystal measurement at 100 K (blue line).

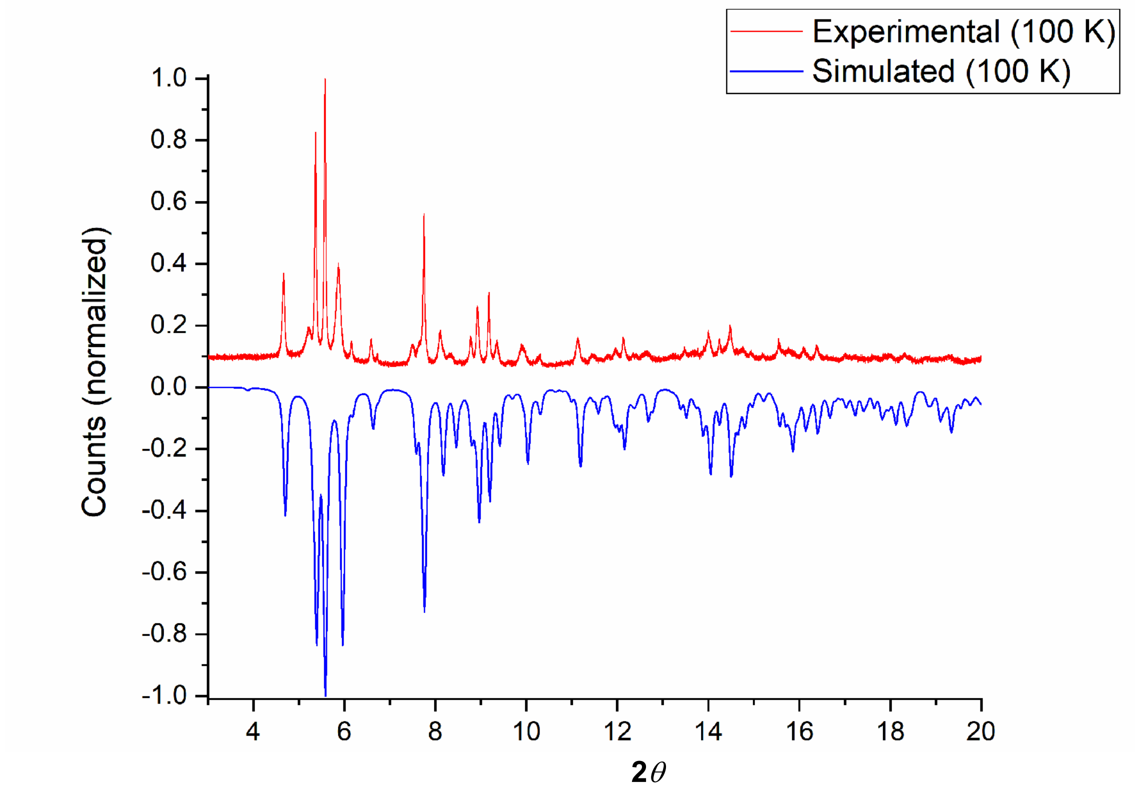

Figure S13. Experimental powder XRD pattern of [**2**][B(C_6F_5)$_4$] at 100 K (red line) compared to the calculated pattern based on the CIF file obtained from a single crystal measurement at 100 K (blue line) up to 2θ = 20°.

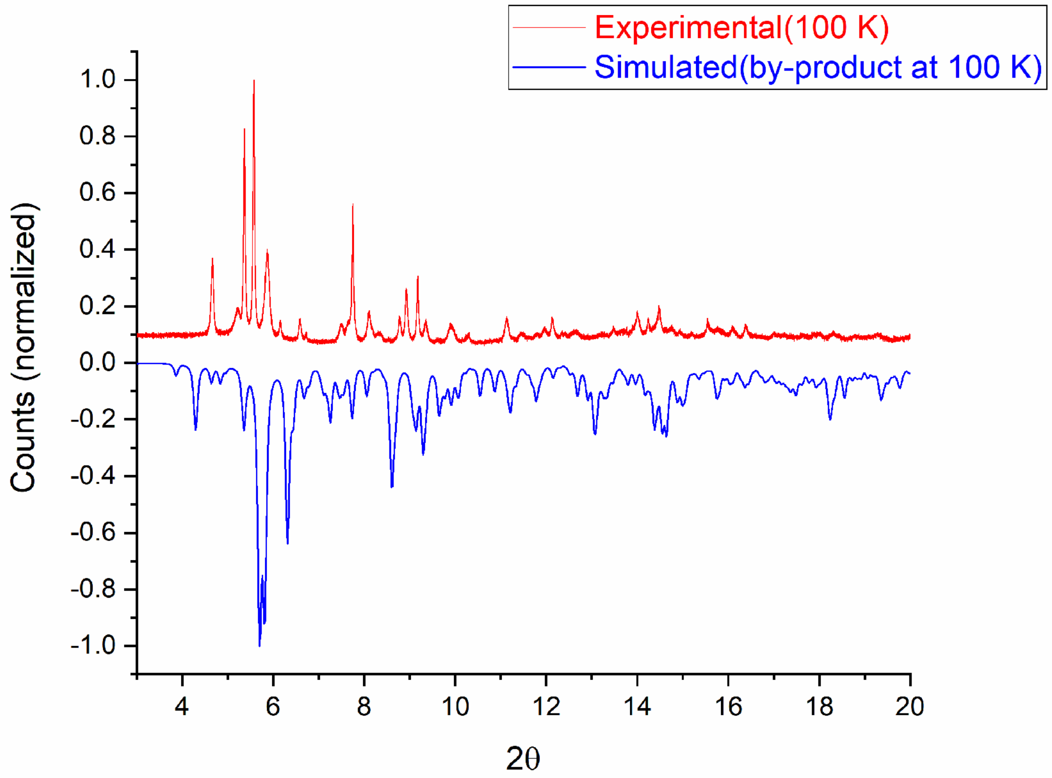

Figure S14. Experimental powder XRD pattern of [**2**][B(C_6F_5)$_4$] at 100 K (red line) compared to the calculated pattern of [**3**][B(C_6F_5)$_4$] based on the CIF file obtained from a single crystal measurement at 100 K (blue line).

Magnetic property measurements

Samples were prepared by adding the crushed crystalline materials and eicosane into 7mm NMR tubes. The tubes were flame sealed under a static vacuum. The eicosane was melted in a water bath at 40°C to prevent crystallite torquing. The direct current (D.C.) magnetic susceptibility and magnetization data were collected using a Quantum Design MPMS3-VSM SQUID magnetometer in cooling mode. Alternating current (A.C.) magnetic susceptibility measurements were performed using a Quantum Design MPMS XL-7 SQUID magnetometer using an oscillating field of 5 Oe. Measurements of the magnetization decay were performed by first magnetizing the sample in a field of 7 T, and then removing the field and measuring the magnetization over time with the VSM option. Diamagnetic corrections were performed using Pascal's coefficients.

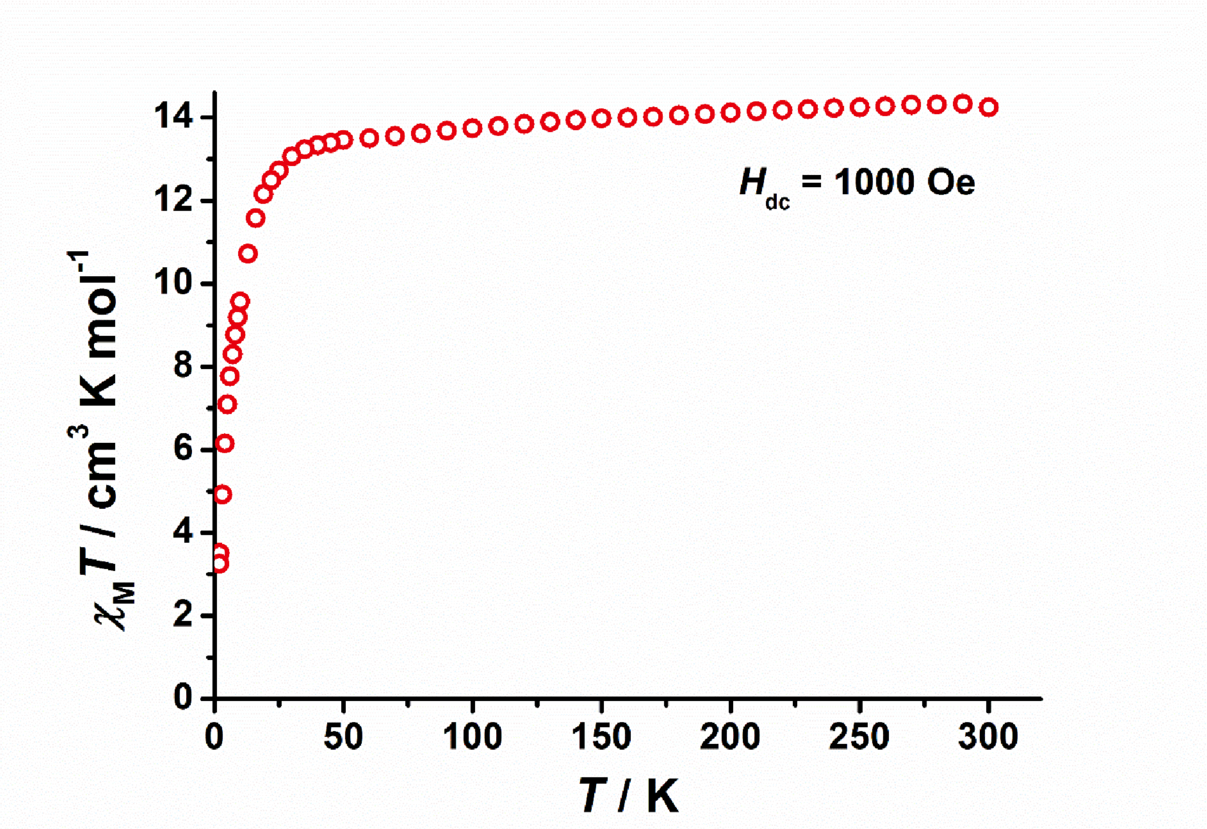

Figure S15. Plot of $\chi_M T$ versus temperature for [**2**][B(C_6F_5)$_4$] in an applied magnetic field of 1 kOe. $\chi_M T$(300 K) = 14.25 cm^3 K mol^{-1}, $\chi_M T$(1.8 K) = 3.25 cm^3 K mol^{-1}.

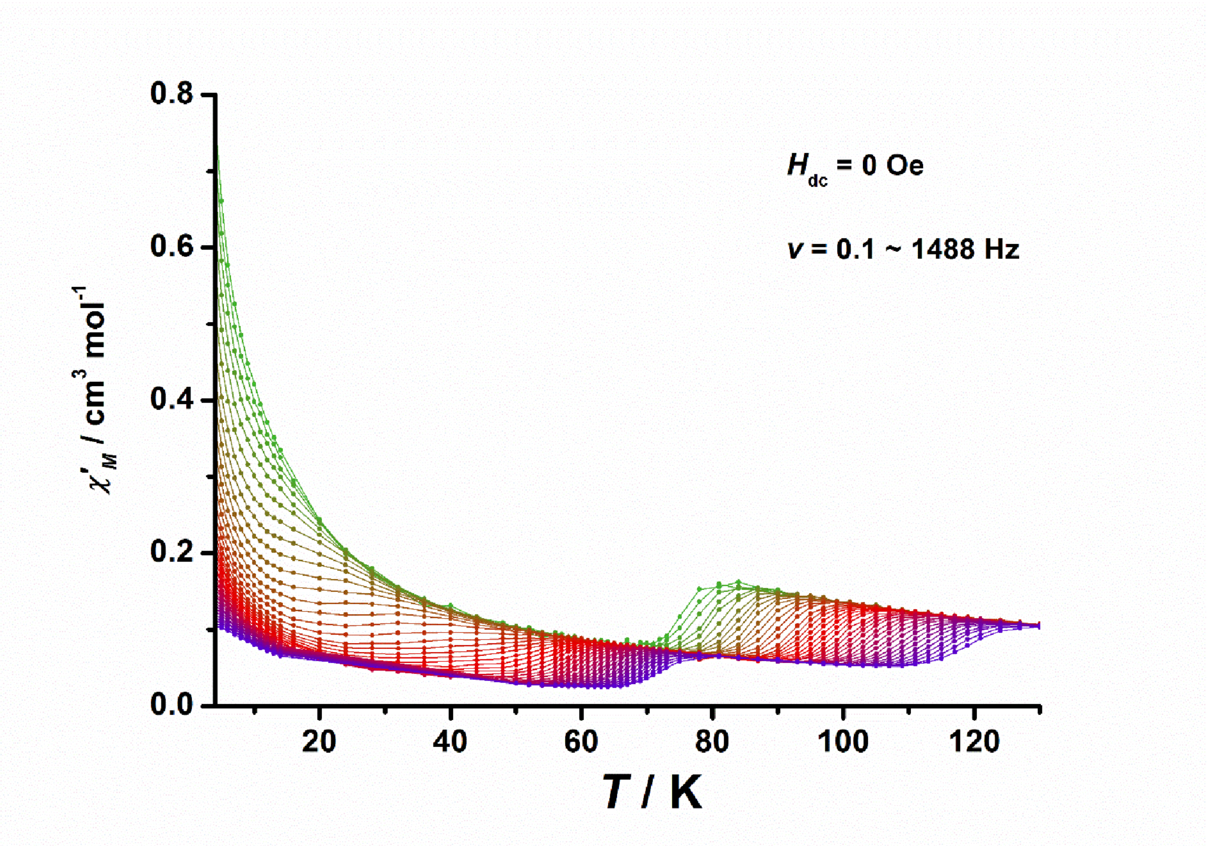

Figure S16. Temperature dependence of the in-phase susceptibility (χ'_M) for [**2**][B(C_6F_5)$_4$] in zero DC field at AC frequencies of 0.1-1488 Hz from 4 to 130 K. Solid lines are a guide to the eye.

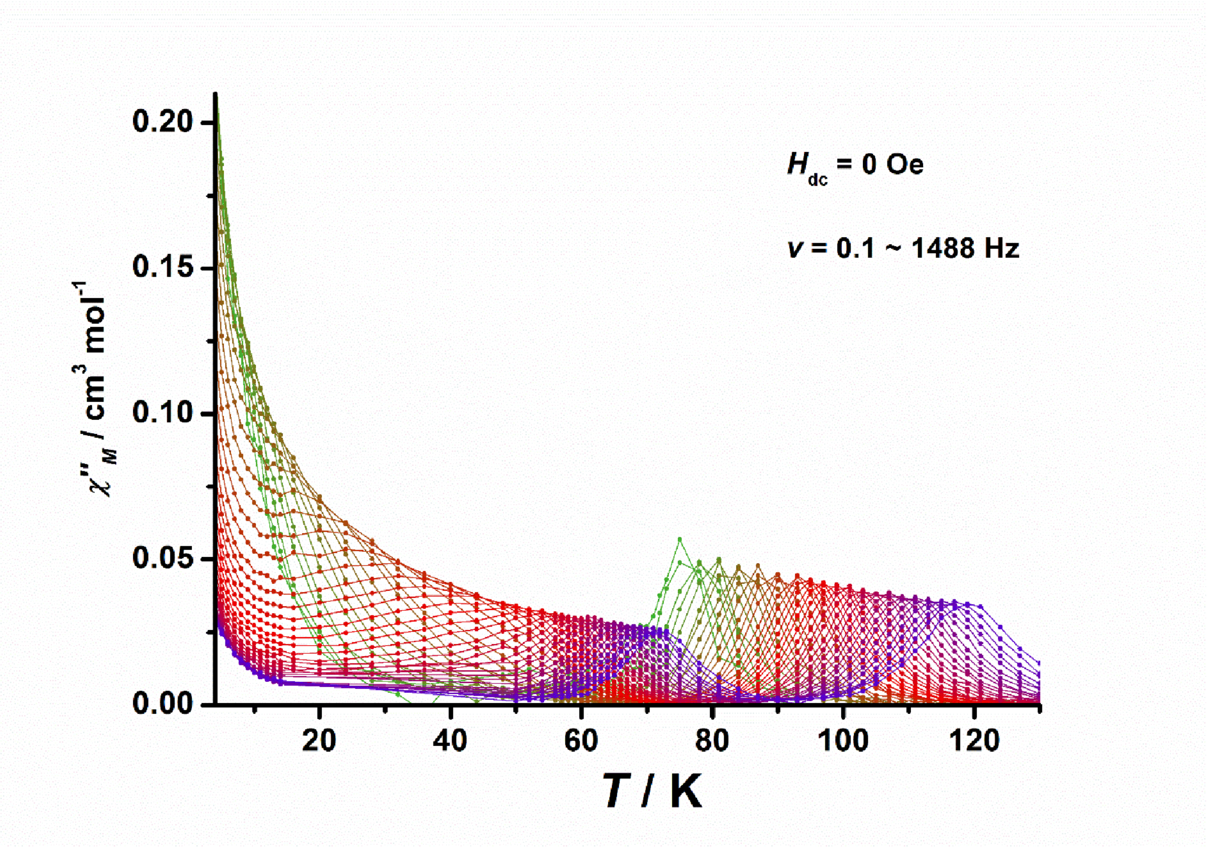

Figure S17. Temperature dependence of the out of phase susceptibility (χ''_M) for [**2**][B(C_6F_5)$_4$] in zero DC field at AC frequencies of 0.1-1488 Hz from 4 to 130 K. Solid lines are a guide to the eye.

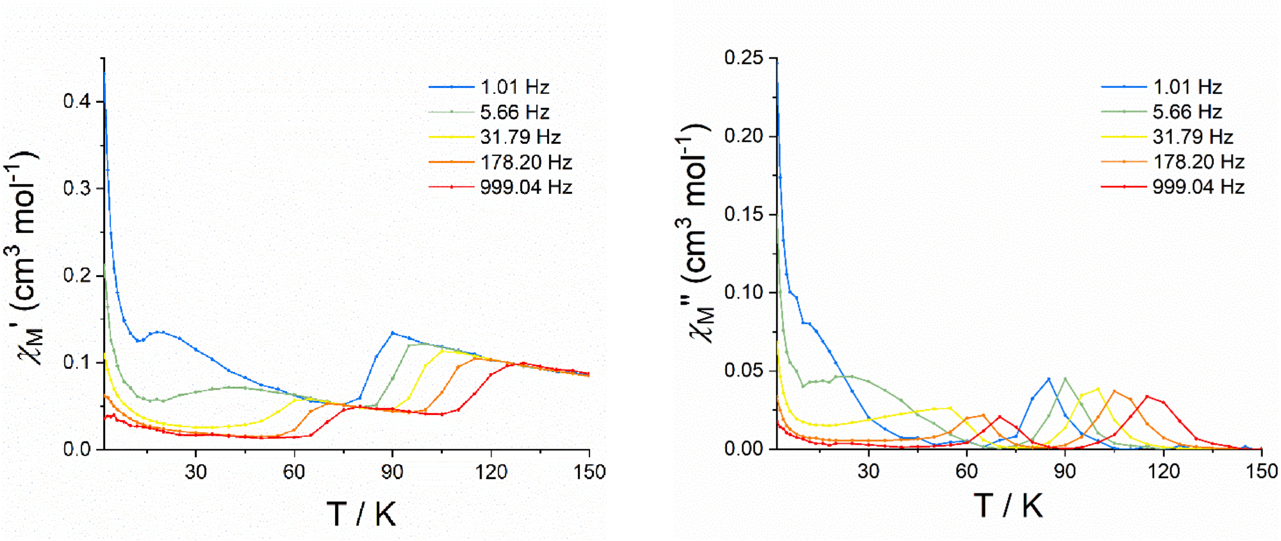

Figure S18. Repeat measurements of $\chi'_M(T)$ and $\chi''_M(T)$ on an independently synthesized batch of of [**2**][B(C_6F_5)$_4$] in zero DC field at the AC frequencies indicated. Solid lines are a guide to the eye.

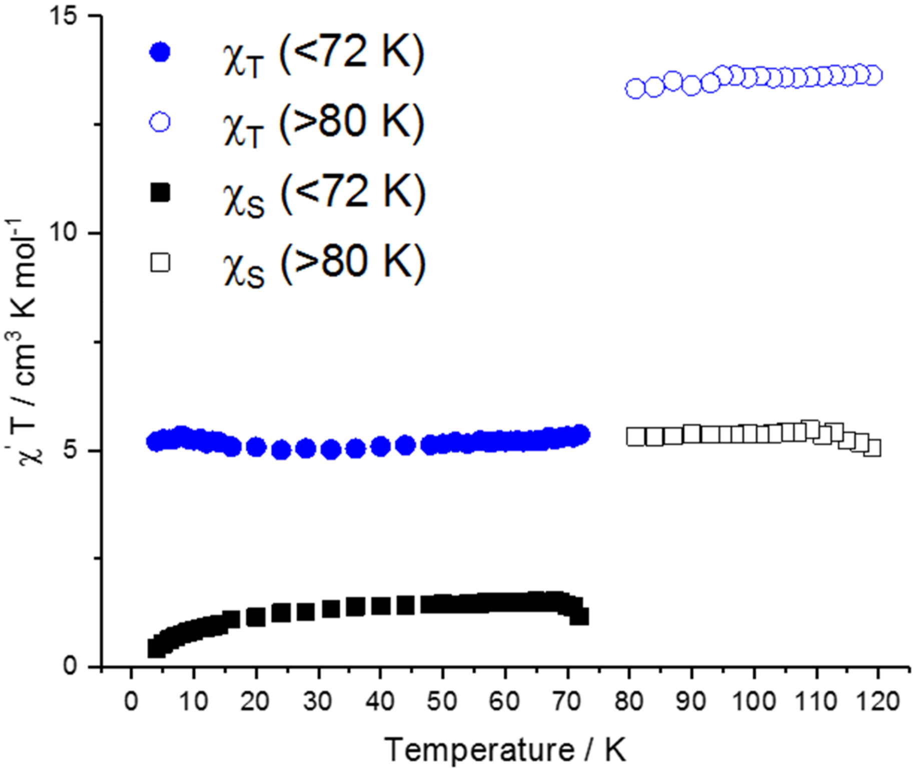

Figure S19. Temperature dependence of the isothermal and adiabatic susceptibility obtained from fitting the frequency-dependent susceptibility data (equation S1 and S2) using a single Cole-Cole model. The temperature dependence suggests two processes with a relative magnitude of 35% of the two relaxation processes.

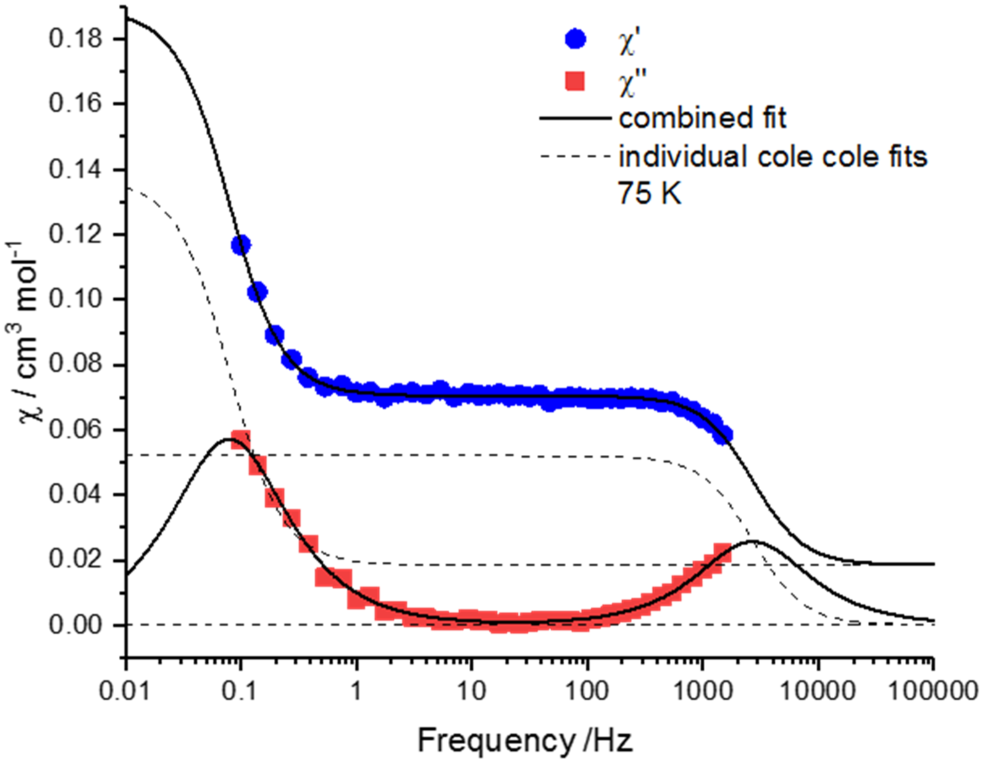

Figure S20. Frequency-dependent AC susceptibility data taken at 75 K, clearly demonstrating two relaxation processes. When fitted to two independent Cole-Cole processes (dashed lines), the resultant fit (solid lines) gives a ratio of the isothermal susceptibility of 38% (0.137 vs 0.052 cm^3 mol^{-1}), strongly suggesting two processes. The data therefore cannot be fitted with two interdependent Cole-Cole processes.

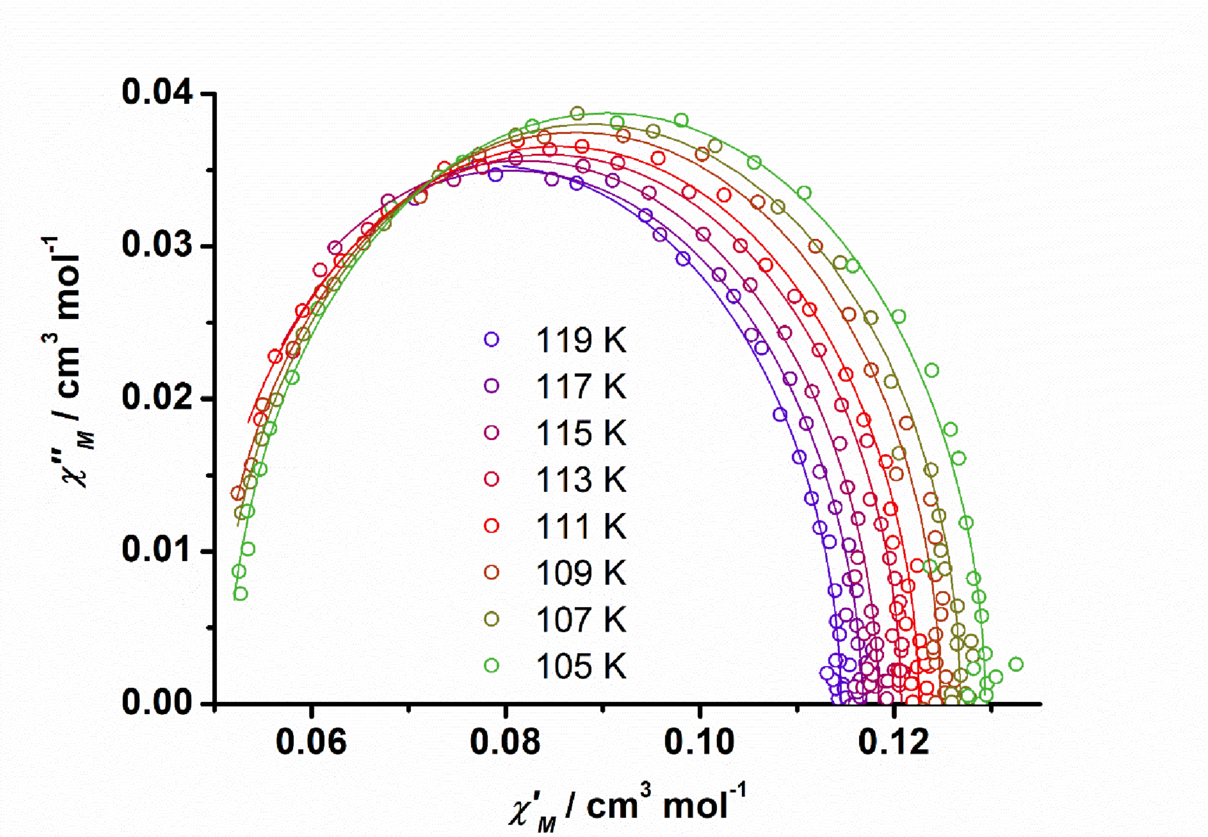

Figure S21. Cole-Cole plots for the AC susceptibilities in zero DC field for [**2**][B(C_6F_5)$_4$] from 105-119 K. Solid lines represent fits to the data using equations 1 and 2, which describe χ' and χ'' in terms of frequency, isothermal susceptibility (χ_T), adiabatic susceptibility (χ_S), relaxation time (τ), and a variable representing the distribution of relaxation times (α).

$$\chi'(\nu_{ac}) = \chi_S + \frac{(\chi_T - \chi_S)[1 + (2\pi\nu_{ac}\tau)^{1-\alpha}\sin(\alpha\pi/2)]}{1 + 2(2\pi\nu_{ac}\tau)^{1-\alpha}\sin(\alpha\pi/2) + (2\pi\nu_{ac}\tau)^{2(1-\alpha)}}$$ Equation S1

$$\chi''(\nu_{ac}) = \frac{(\chi_T - \chi_S)(2\pi\nu_{ac}\tau)^{1-\alpha}\cos(\alpha\pi/2)}{1 + 2(2\pi\nu_{ac}\tau)^{1-\alpha}\sin(\alpha\pi/2) + (2\pi\nu_{ac}\tau)^{2(1-\alpha)}}$$ Equation S2

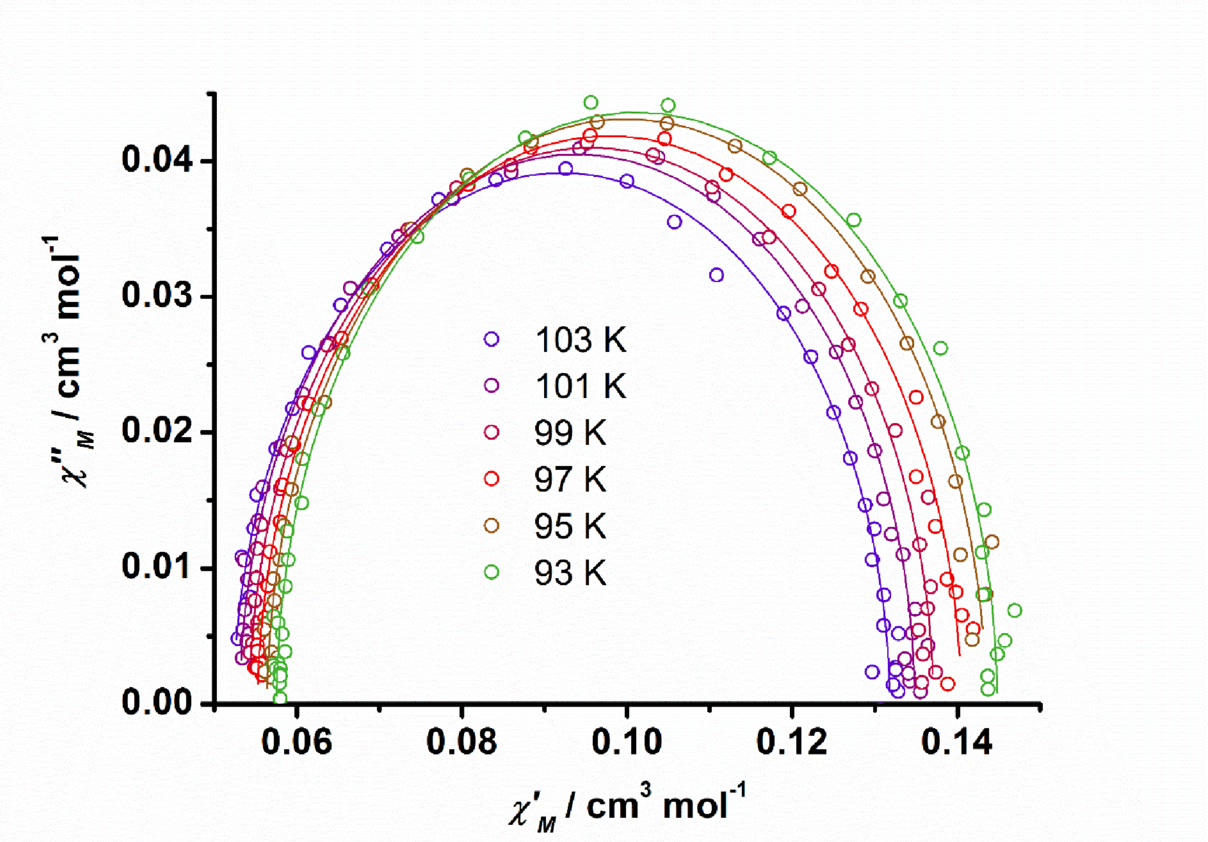

Figure S22. Cole-Cole plots for the AC susceptibilities in zero DC field for [**2**][B(C_6F_5)$_4$] from 93-103 K. Solid lines represent fits to the data using equations 1 and 2.

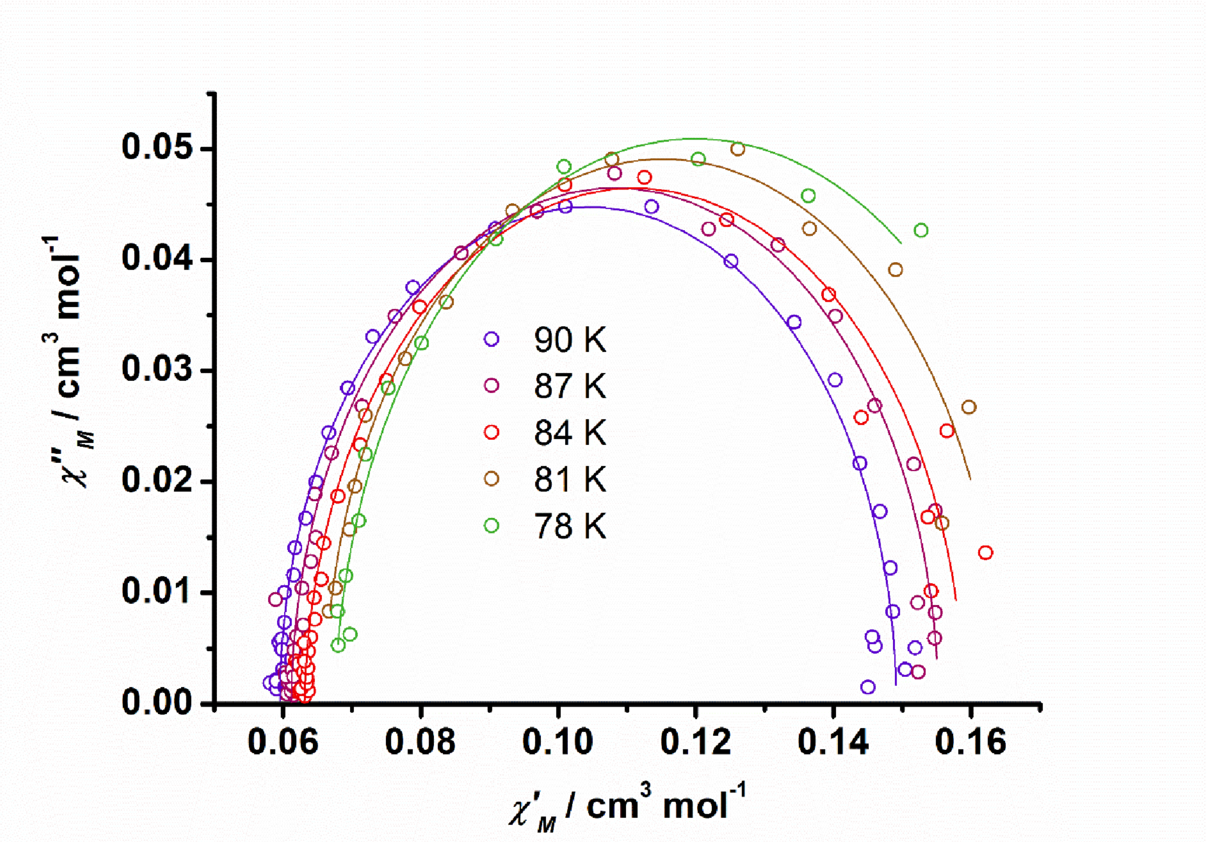

Figure S23. Cole-Cole plots for the AC susceptibilities in zero DC field for [**2**][B(C_6F_5)$_4$] from 78-90 K. Solid lines represent fits to the data using equations 1 and 2.

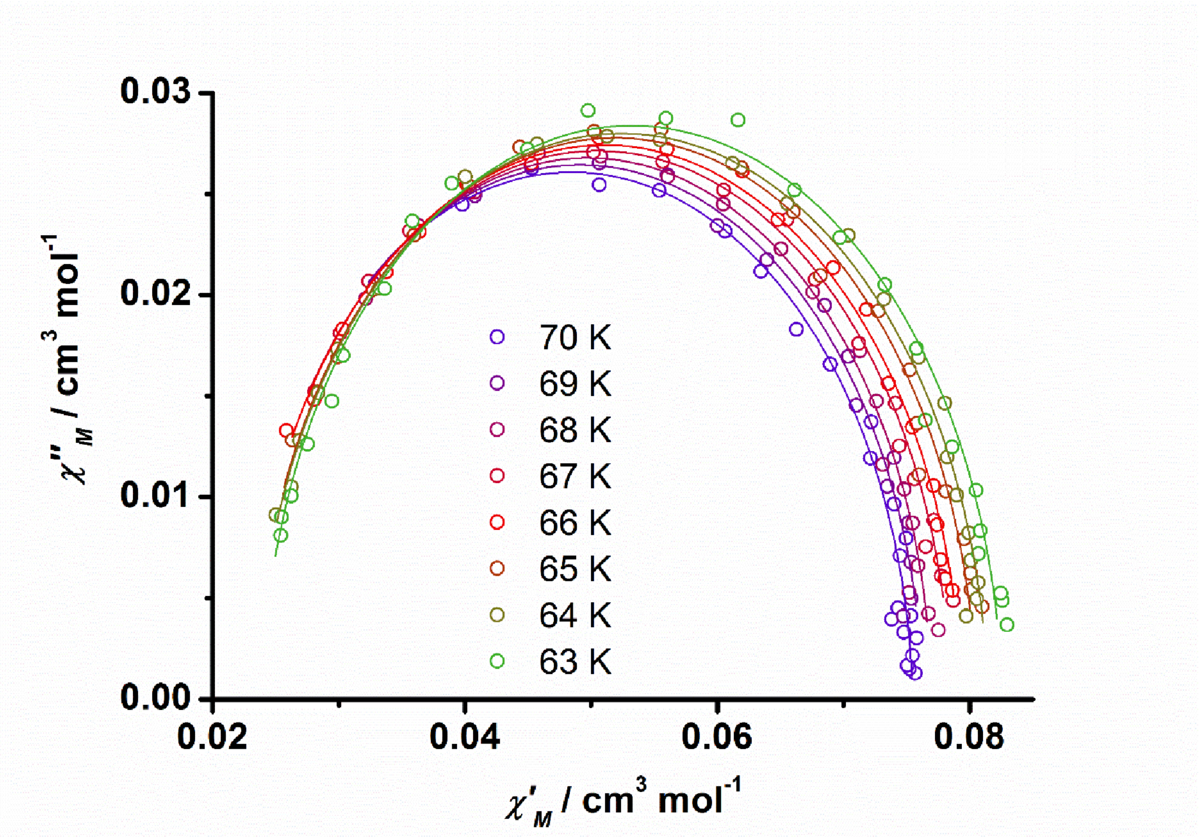

Figure S24. Cole-Cole plots for the AC susceptibilities in zero DC field for [**2**][$B(C_6F_5)_4$] from 63-70 K. Solid lines represent fits to the data using equations 1 and 2.

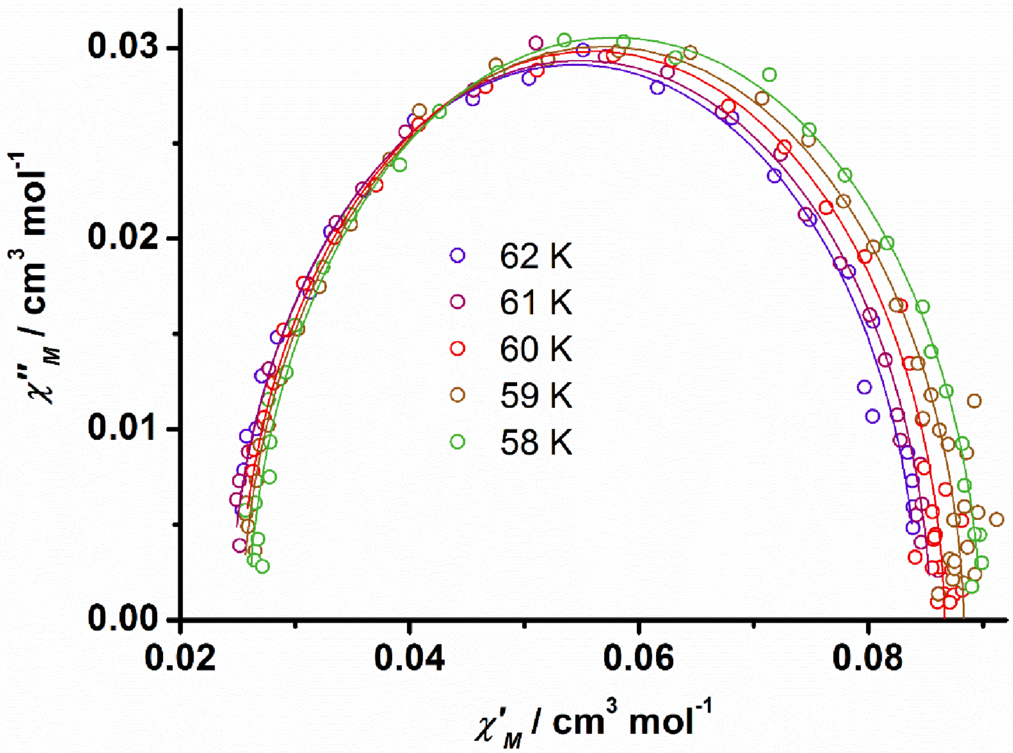

Figure S25. Cole-Cole plots for the AC susceptibilities in zero DC field for [**2**][B(C_6F_5)$_4$] from 58-62 K. Solid lines represent fits to the data using equations 1 and 2.

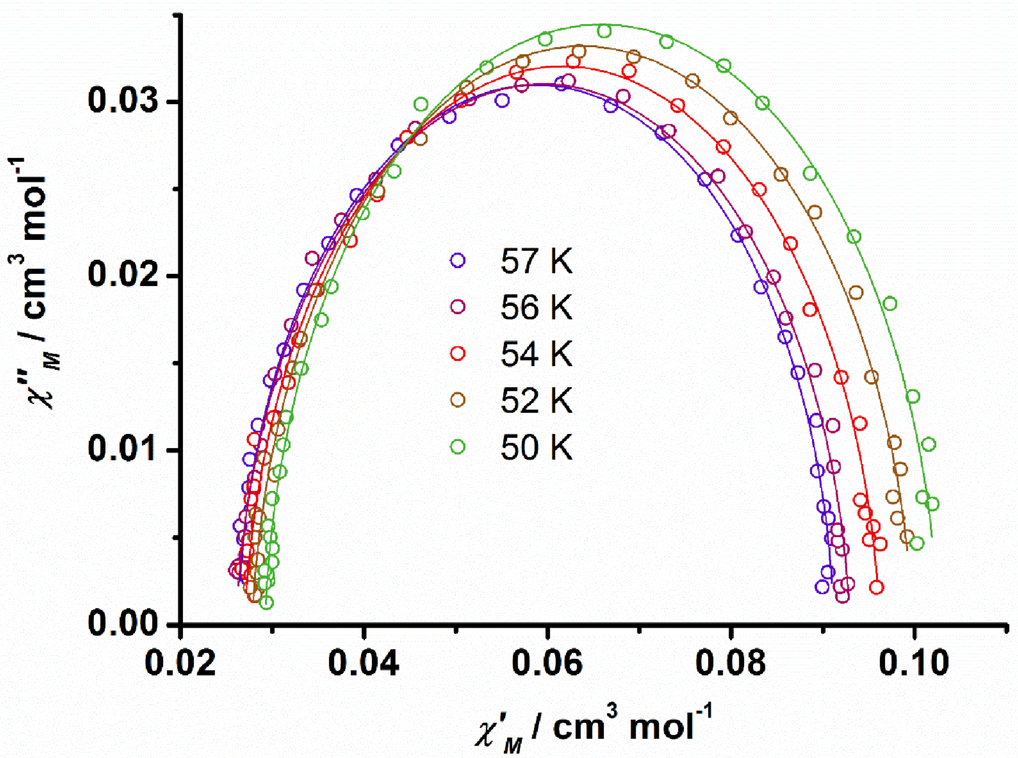

Figure S26. Cole-Cole plots for the AC susceptibilities in zero DC field for [**2**][B(C_6F_5)$_4$] from 50-57 K. Solid lines represent fits to the data using equations 1 and 2.

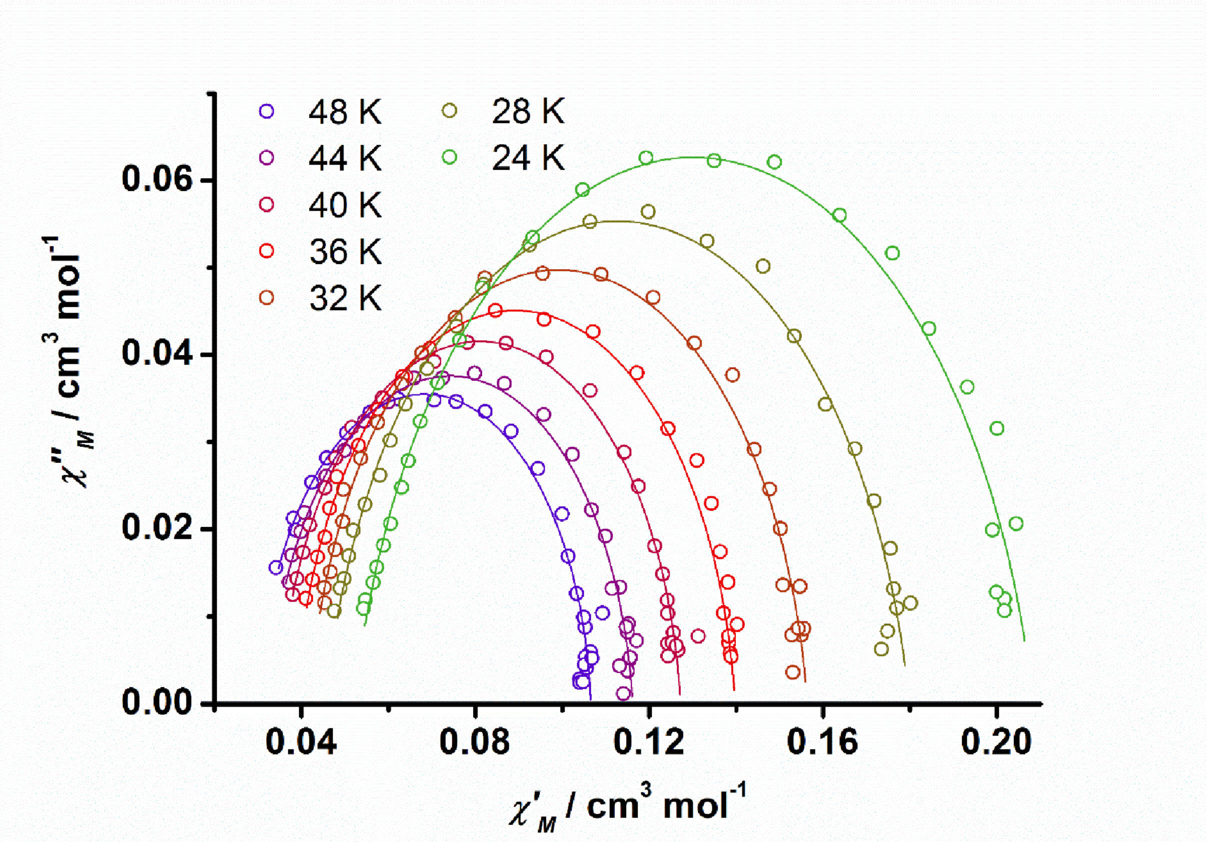

Figure S27. Cole-Cole plots for the AC susceptibilities in zero DC field for [**2**][B(C_6F_5)$_4$] from 32-48 K. Solid lines represent fits to the data using equations 1 and 2.

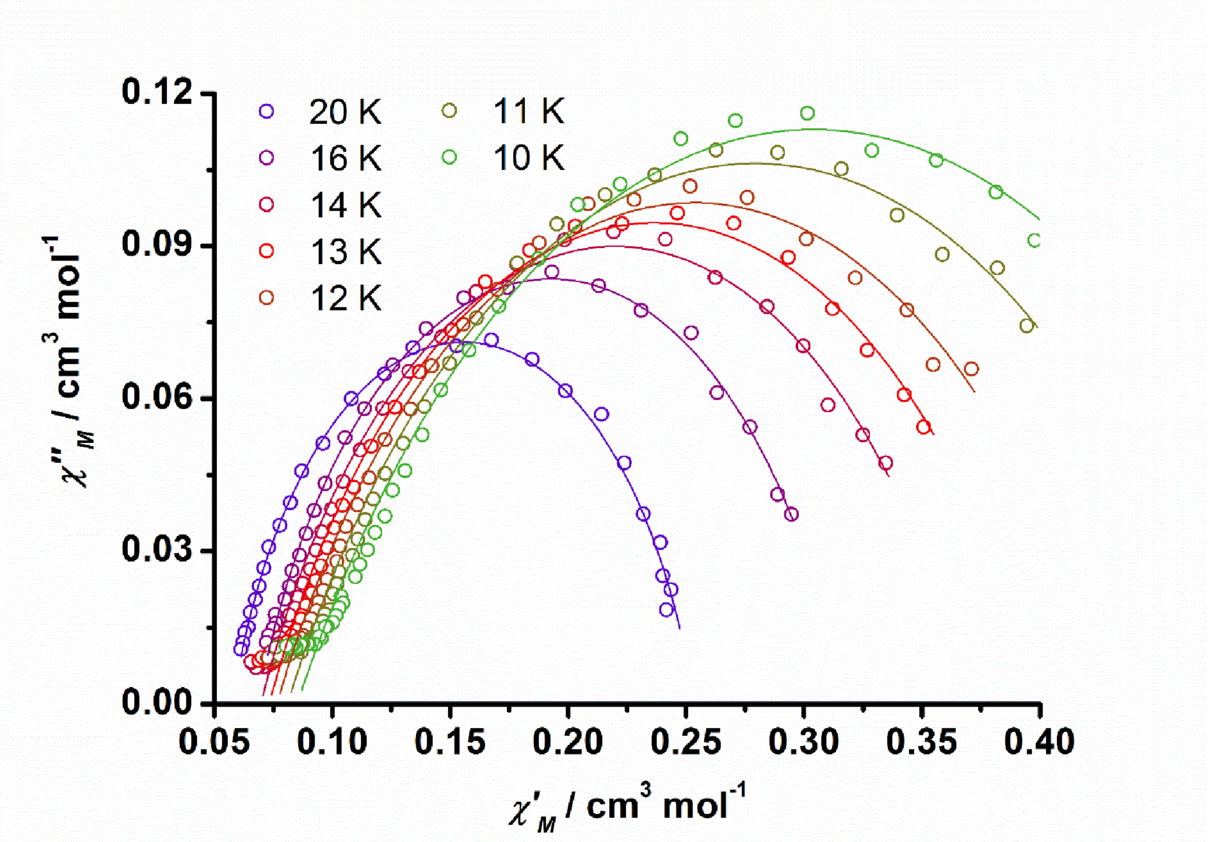

Figure S28. Cole-Cole plots for the AC susceptibilities in zero DC field for [**2**][B(C_6F_5)$_4$] from 10-20 K. Solid lines represent fits to the data using equations 1 and 2.

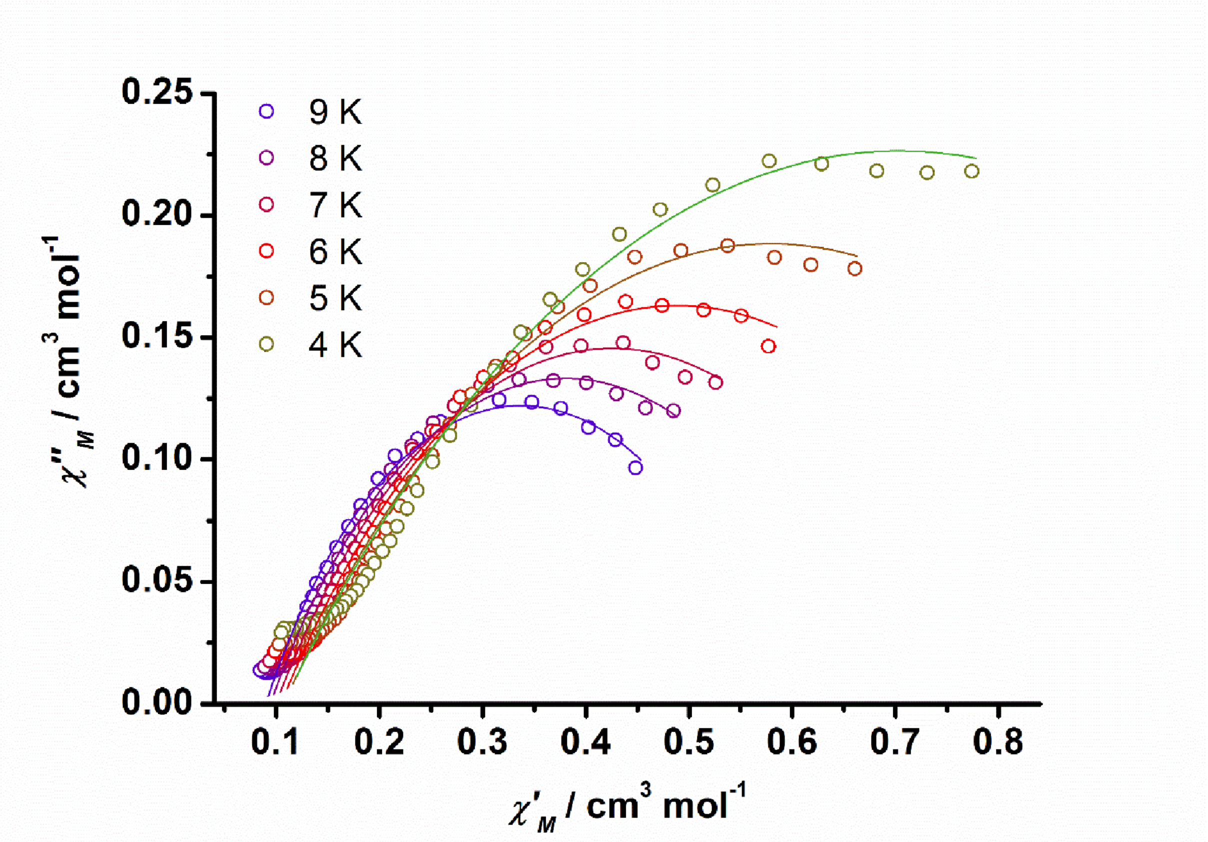

Figure S29. Cole-Cole plots for the AC susceptibilities in zero DC field for [**2**][B(C_6F_5)$_4$] from 4-9 K. Solid lines represent fits to the data using equations 1 and 2.

Table S7. Relaxation fitting parameters for [**2**][$B(C_6F_5)_4$] corresponding to Figures. S11-S13.

T / K	χ_T / cm^3 mol^{-1}	χ_S / cm^3 mol^{-1}	α	τ / s
119	0.11454	0.04239	0.0142	1.03182E-4
117	0.11677	0.04417	0.02389	1.43932E-4
115	0.11843	0.04535	0.01614	2.01127E-4
113	0.12072	0.04796	0.00643	2.90005E-4
111	0.12259	0.04818	0.01097	4.0449E-4
109	0.12479	0.04983	0 [a]	5.79921E-4
107	0.12688	0.05052	0.00263	8.36849E-4
105	0.12937	0.05141	0.00385	0.00122
103	0.1318	0.05231	0.00967	0.00183
101	0.13484	0.05307	0.00565	0.00271
99	0.13709	0.05424	0.00624	0.00418
97	0.14046	0.0552	0.01123	0.00642
95	0.14345	0.05636	0.00598	0.01005
93	0.1448	0.05755	0 [a]	0.01552
90	0.1491	0.0595	0 [a]	0.03163
87	0.15523	0.06118	0.007	0.06791
84	0.15905	0.06309	0.02019	0.15061
81	0.16428	0.06611	0 [a]	0.34238
78	0.17215	0.06755	0.01699	0.80502

[a] These parameter values were restricted to non-negative.

Table S8. Relaxation fitting parameters for [**2**][$B(C_6F_5)_4$] corresponding to Figures. S14-S19.

T / K	χ_T / cm^3 mol^{-1}	χ_S / cm^3 mol^{-1}	α	τ / s
70	0.07548	0.02146	0.02215	1.8161E-4
69	0.07627	0.02197	0.01665	2.2755E-4
68	0.07697	0.02252	0.01017	2.84347E-4
67	0.0785	0.02258	0.01884	3.62356E-4
66	0.07936	0.02268	0.02073	4.47172E-4
65	0.0805	0.02335	0.01776	5.74249E-4
64	0.08144	0.02341	0.02261	7.10332E-4
63	0.08256	0.02387	0.02105	9.04734E-4
62	0.08438	0.02435	0.01917	0.00113
61	0.08548	0.02429	0.02641	0.00141
60	0.08665	0.02513	0.01929	0.00174
59	0.08834	0.02533	0.02904	0.00212
58	0.08981	0.02589	0.02848	0.00263
57	0.09118	0.02624	0.02953	0.0031
56	0.09295	0.02608	0.04714	0.00369
54	0.09621	0.02699	0.04871	0.00489
52	0.09978	0.02779	0.05102	0.00629
50	0.10256	0.0292	0.03972	0.00753
48	0.10658	0.03013	0.04691	0.00895
44	0.11618	0.03242	0.06849	0.01175
40	0.12722	0.03495	0.06637	0.01478
36	0.13976	0.03866	0.07221	0.01892
32	0.15641	0.04181	0.0893	0.02441
28	0.17986	0.04556	0.12212	0.03379
24	0.20838	0.05204	0.13938	0.04776
20	0.25345	0.05772	0.19998	0.073
16	0.31724	0.06827	0.24742	0.11897
14	0.3704	0.06964	0.31382	0.1635
13	0.40014	0.0732	0.33207	0.19192
12	0.43047	0.07649	0.35323	0.22265
11	0.47763	0.08094	0.37372	0.27257
10	0.52278	0.08505	0.39349	0.31875
9	0.585	0.08967	0.41689	0.38696
8	0.6675	0.09409	0.44595	0.49188
7	0.75236	0.09939	0.46594	0.56904
6	0.87338	0.10416	0.4894	0.68014
5	1.0506	0.10728	0.51602	0.84022
4	1.30082	0.10676	0.53832	0.99147

[a] These parameter values were restricted to non-negative.

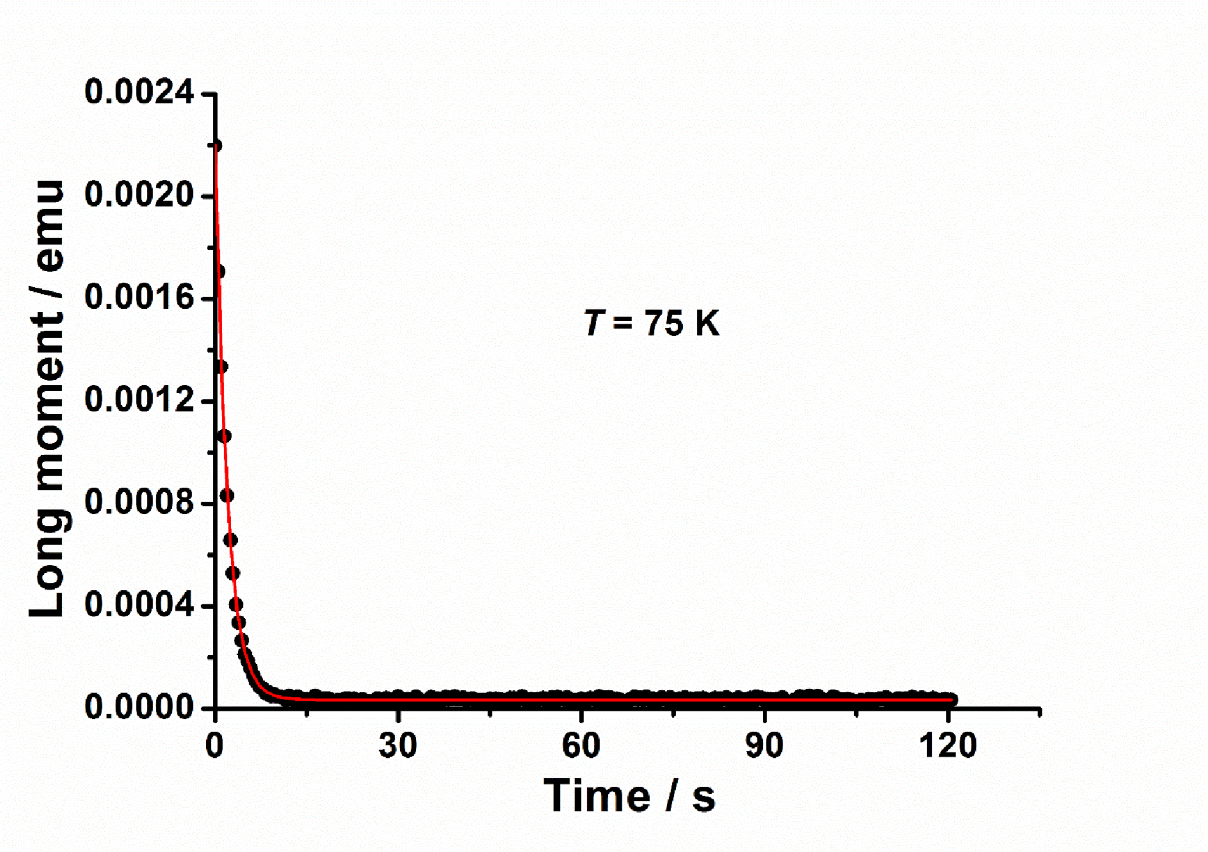

Figure S30. Plot of magnetization decay *vs.* time used to derive DC relaxation times for [**2**][B(C_6F_5)$_4$] at 75 K. The solid lines are the best fit to the exponential decay as $M(t) = M_f + (M_0 - M_f)\ \exp[(-(t/\tau)^{\beta}]$, where M_0 is the initial magnetization, M_f is the final magnetization, τ is the relaxation time, and β is a generalized coefficient, which should be equal to 1 for an ideal exponential decay.

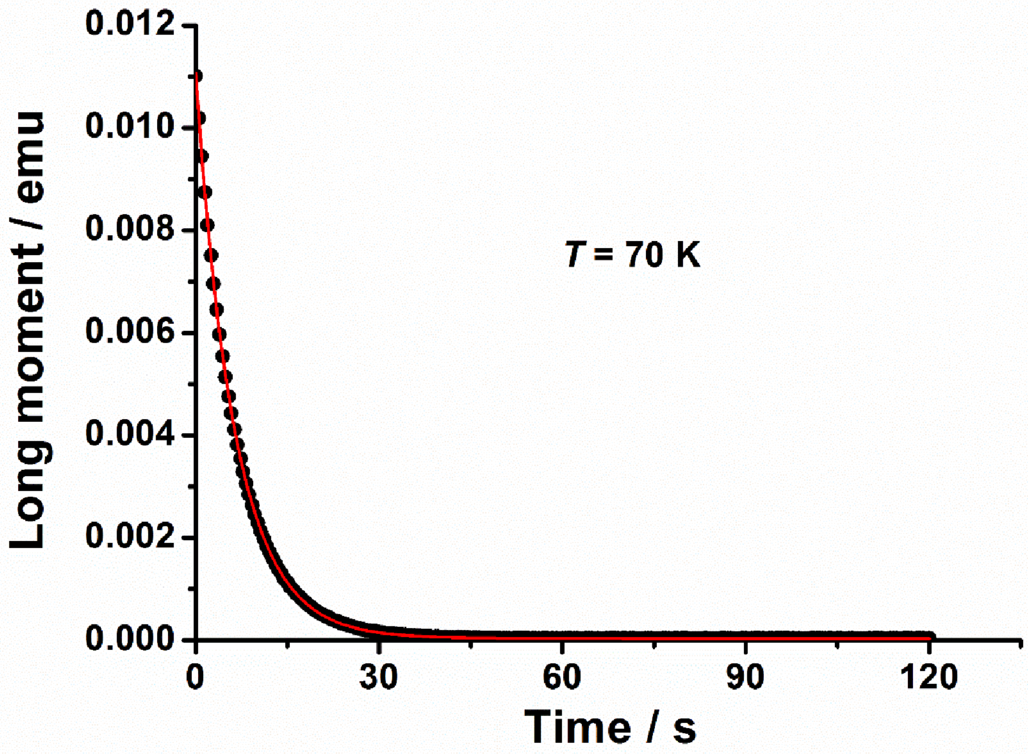

Figure S31. Plot of magnetization decay *vs.* time used to derive relaxation times for [**2**][$B(C_6F_5)_4$] at 70 K.

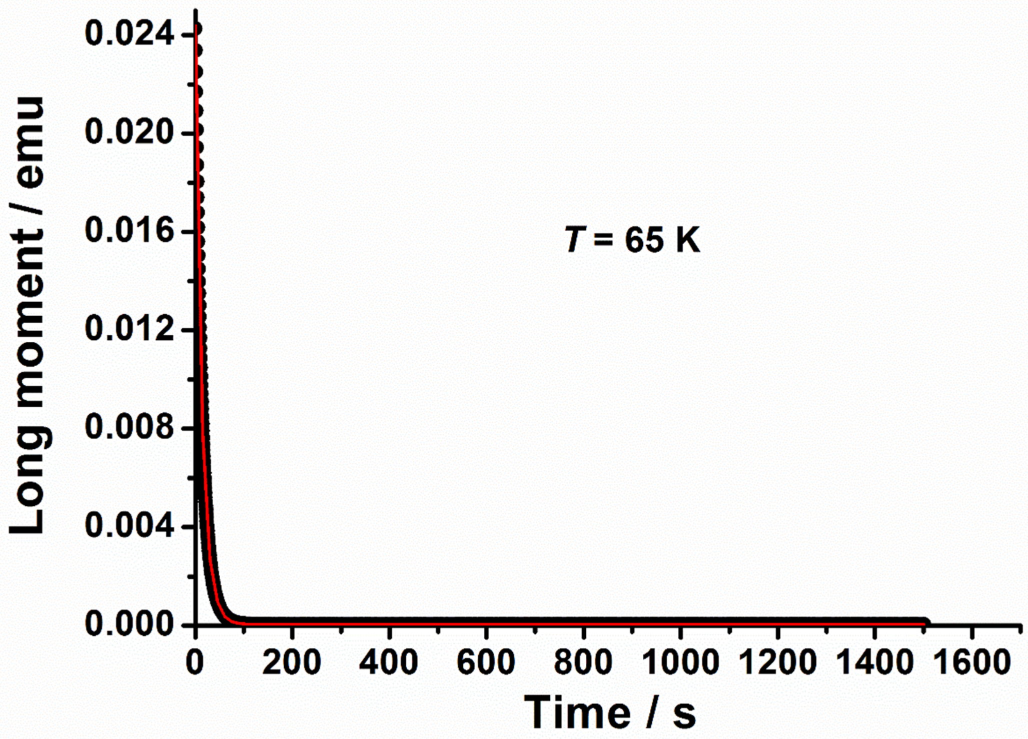

Figure S32. Plot of magnetization decay *vs.* time used to derive relaxation times for [**2**][B(C_6F_5)$_4$] at 65 K.

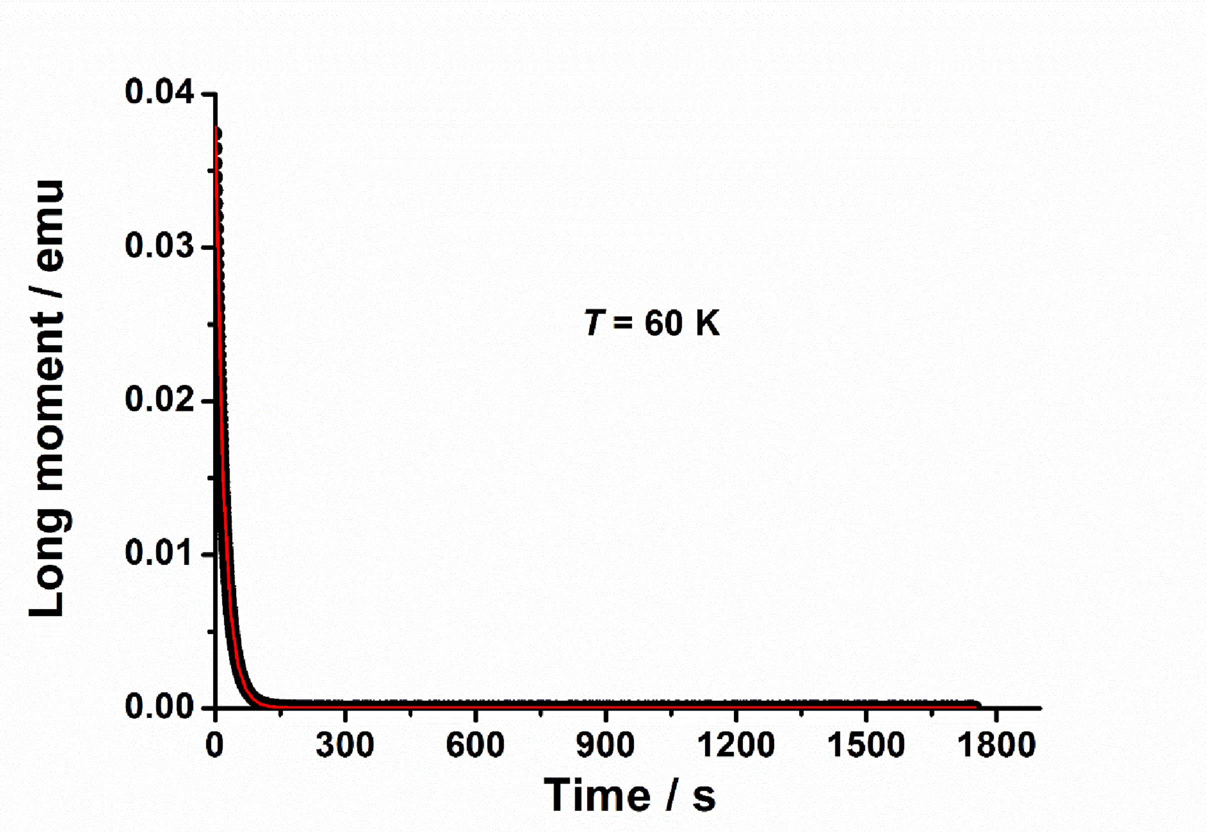

Figure S33. Plot of magnetization decay *vs.* time used to derive relaxation times for [**2**][$B(C_6F_5)_4$] at 60 K.

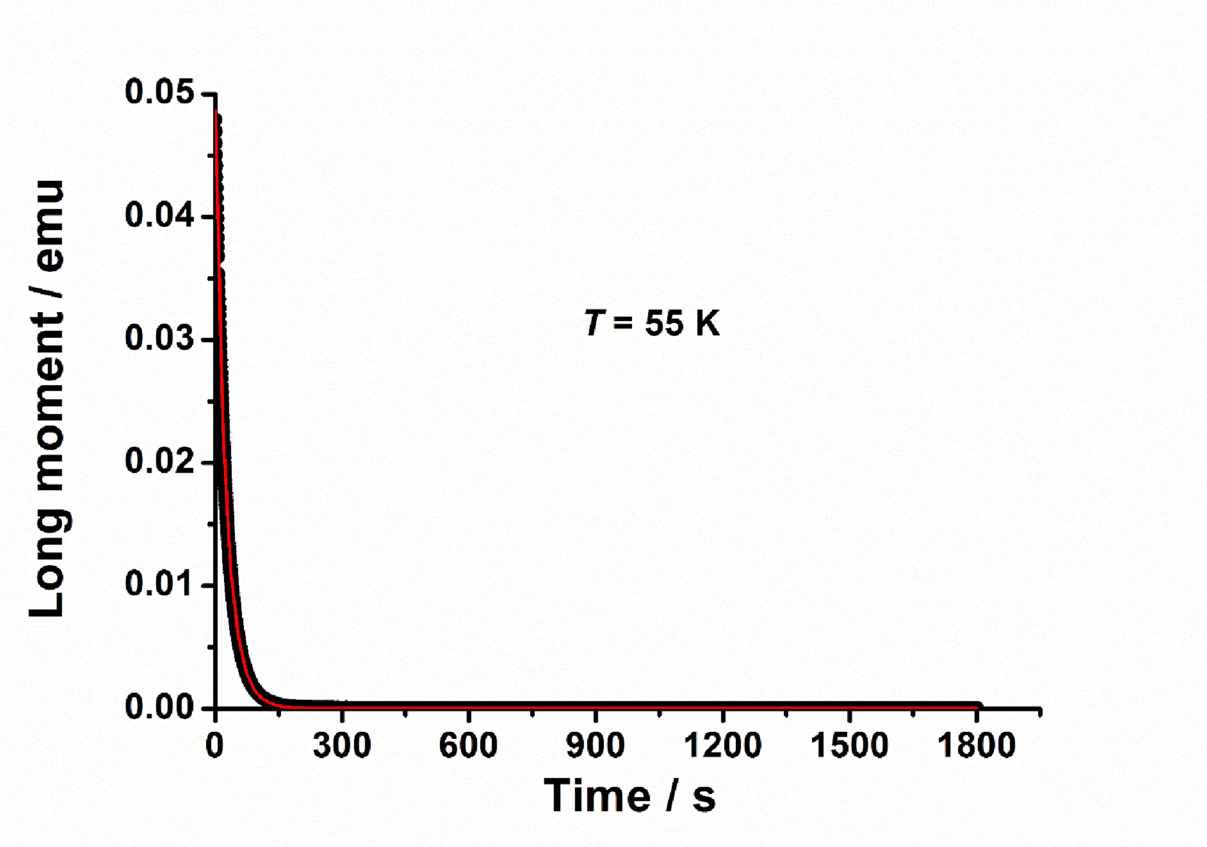

Figure S34. Plot of magnetization decay *vs.* time used to derive relaxation times for [**2**][$B(C_6F_5)_4$] at 55 K.

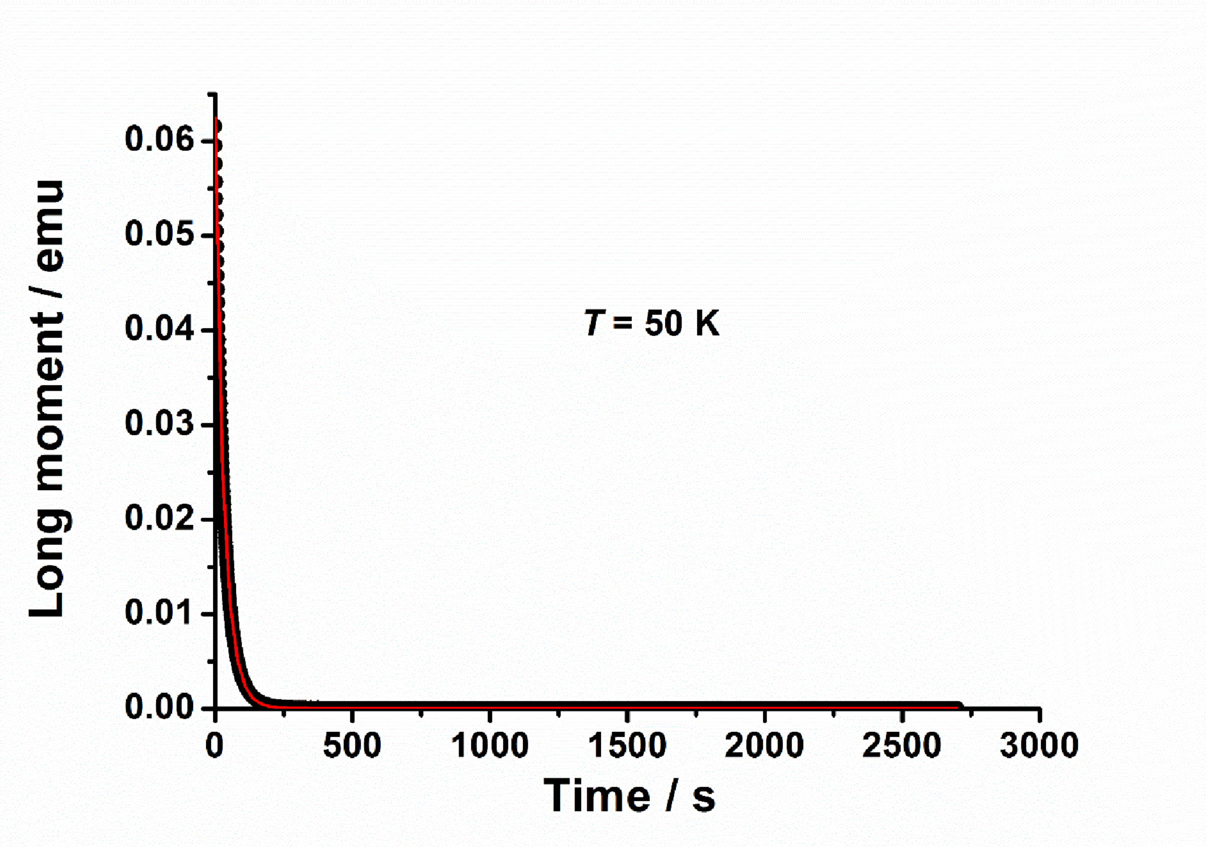

Figure S35. Plot of magnetization decay *vs.* time used to derive relaxation times for [**2**][B(C_6F_5)$_4$] at 50 K.

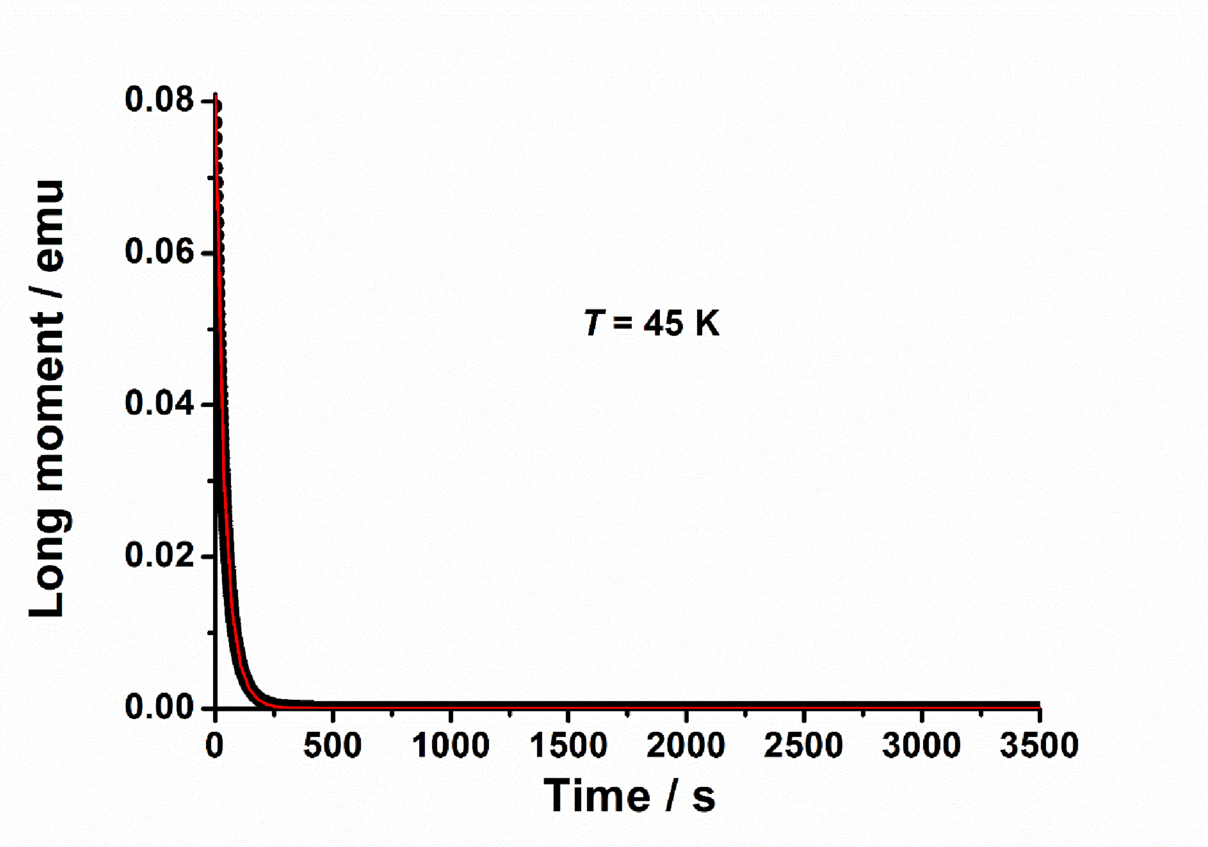

Figure S36. Plot of magnetization decay *vs.* time used to derive relaxation times for [**2**][B(C_6F_5)$_4$] at 45 K.

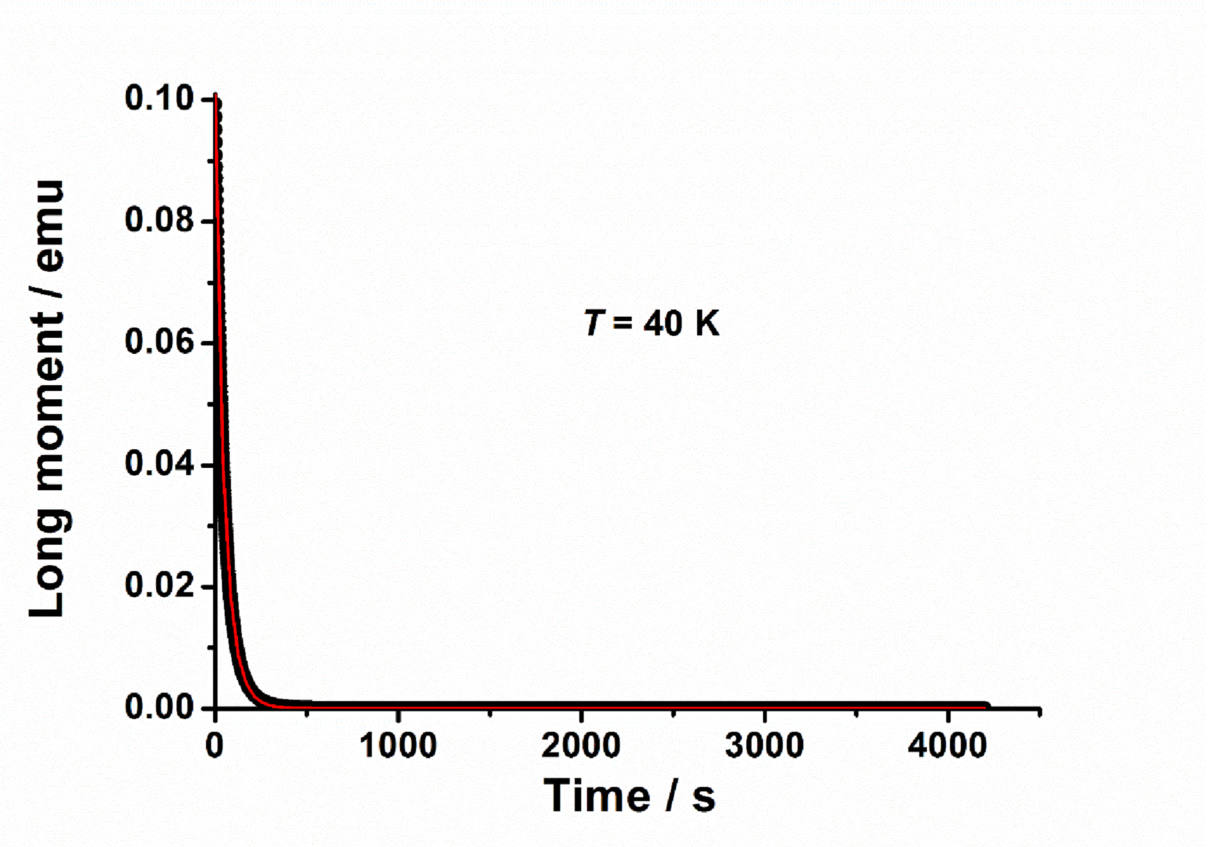

Figure S37. Plot of magnetization decay *vs.* time used to derive relaxation times for [**2**][$B(C_6F_5)_4$] at 40 K.

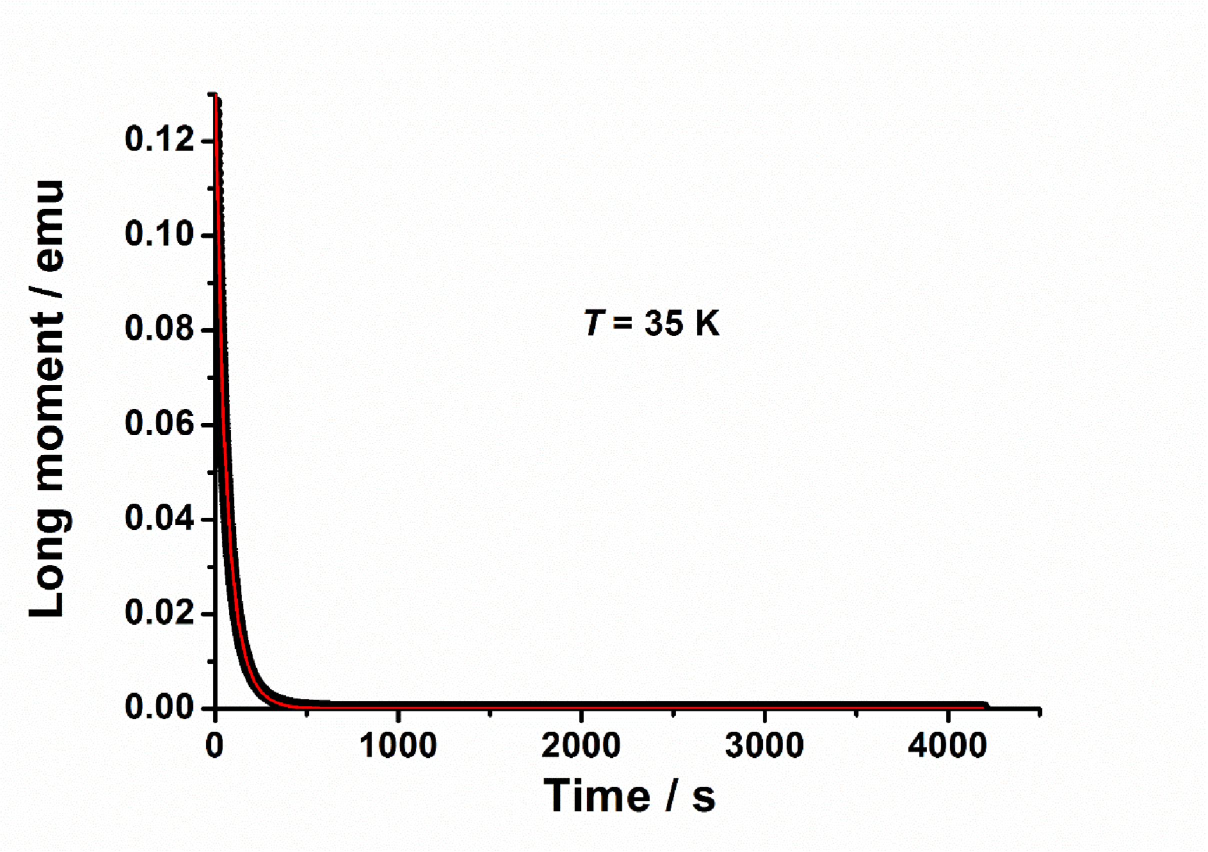

Figure S38. Plot of magnetization decay *vs.* time used to derive relaxation times for [**2**][B(C_6F_5)$_4$] at 35 K.

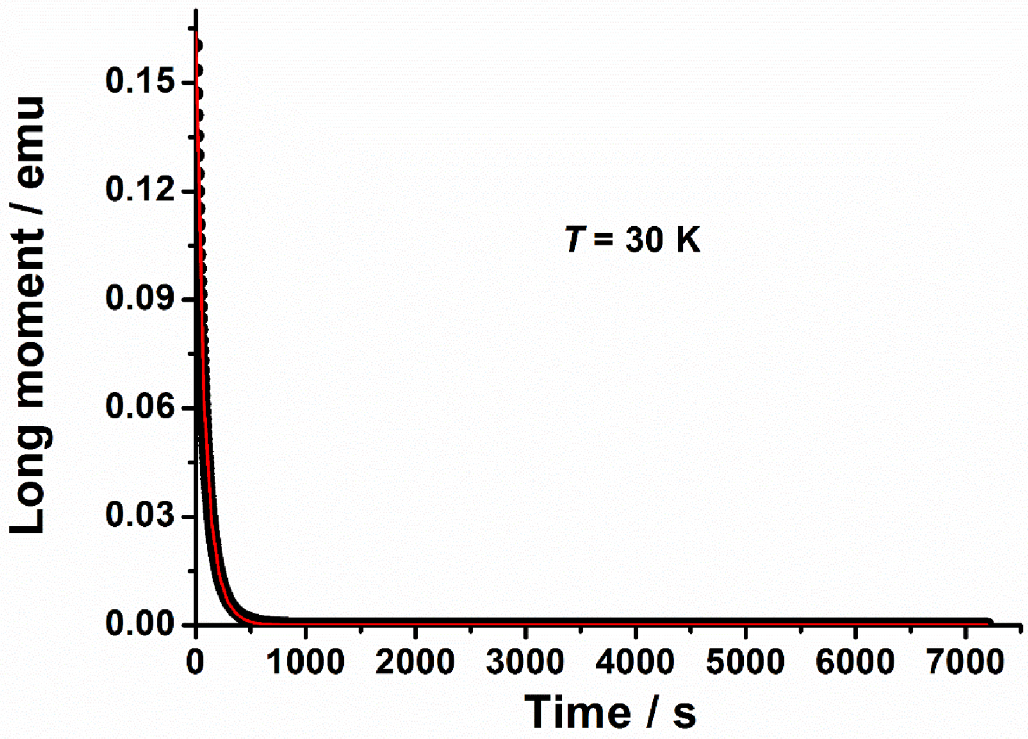

Figure S39. Plot of magnetization decay *vs.* time used to derive relaxation times for [**2**][$B(C_6F_5)_4$] at 30 K.

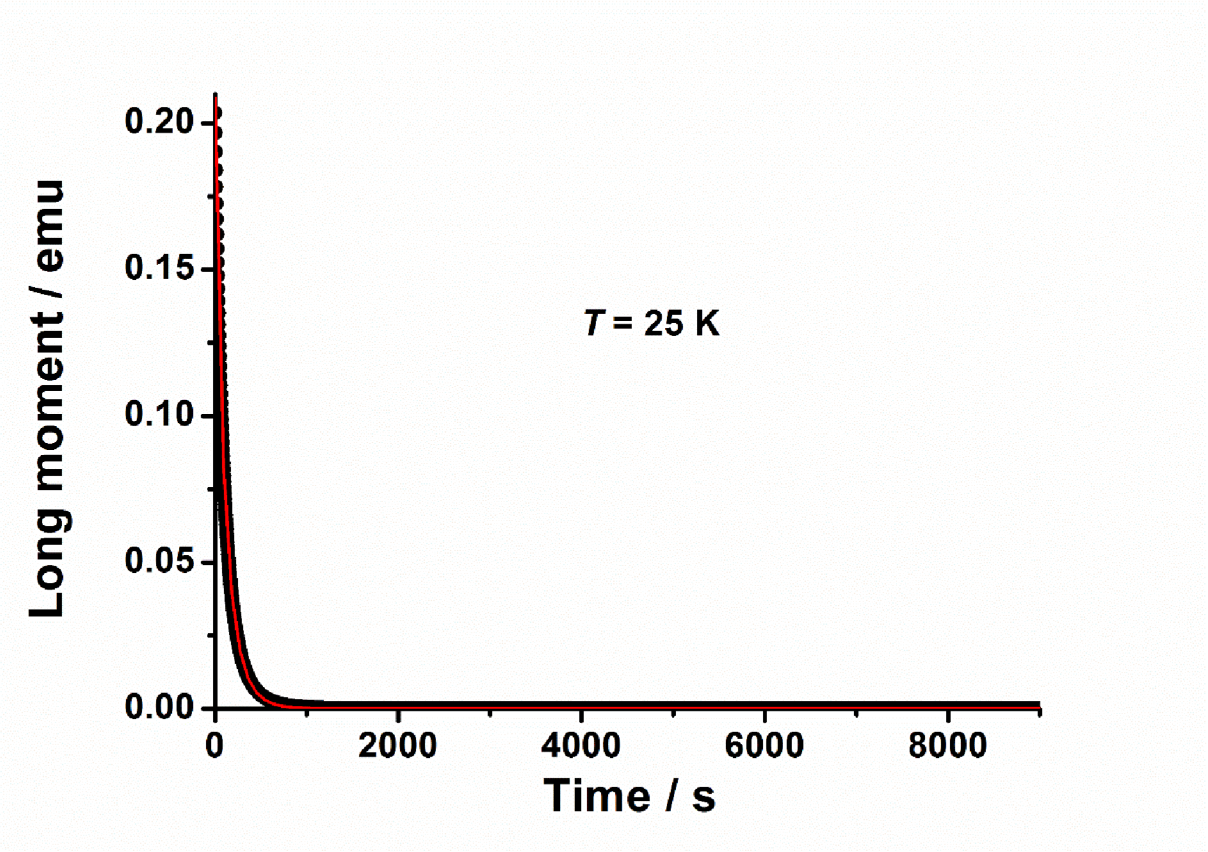

Figure S40. Plot of magnetization decay *vs.* time used to derive relaxation times for [**2**][B(C_6F_5)$_4$] at 25 K.

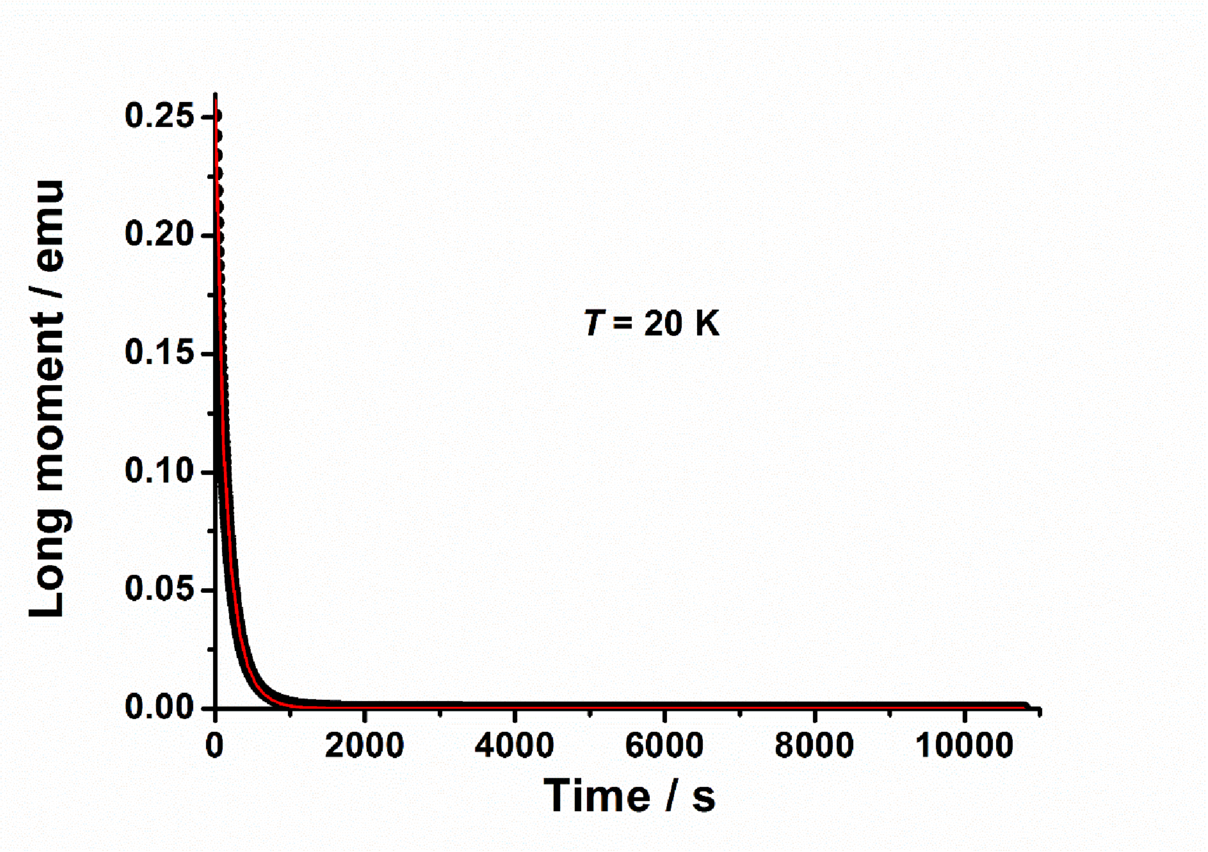

Figure S41. Plot of magnetization decay *vs.* time used to derive relaxation times for [**2**][B(C_6F_5)$_4$] at 20 K.

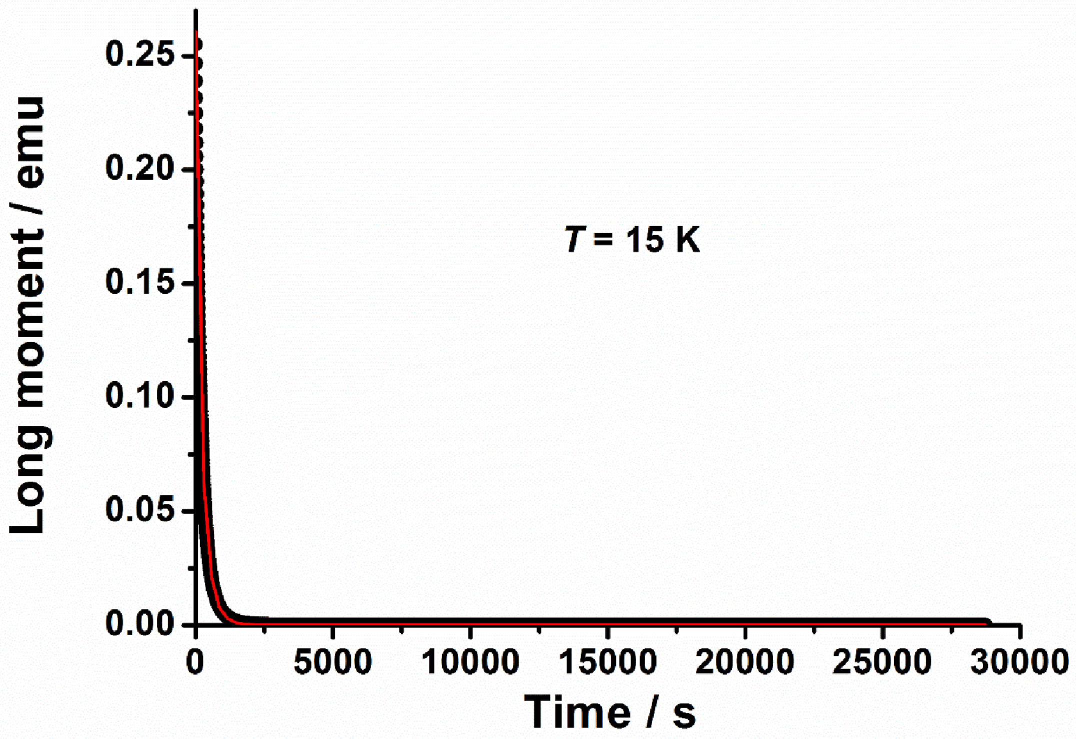

Figure S42. Plot of magnetization decay *vs.* time used to derive relaxation times for [**2**][B(C_6F_5)$_4$] at 15 K.

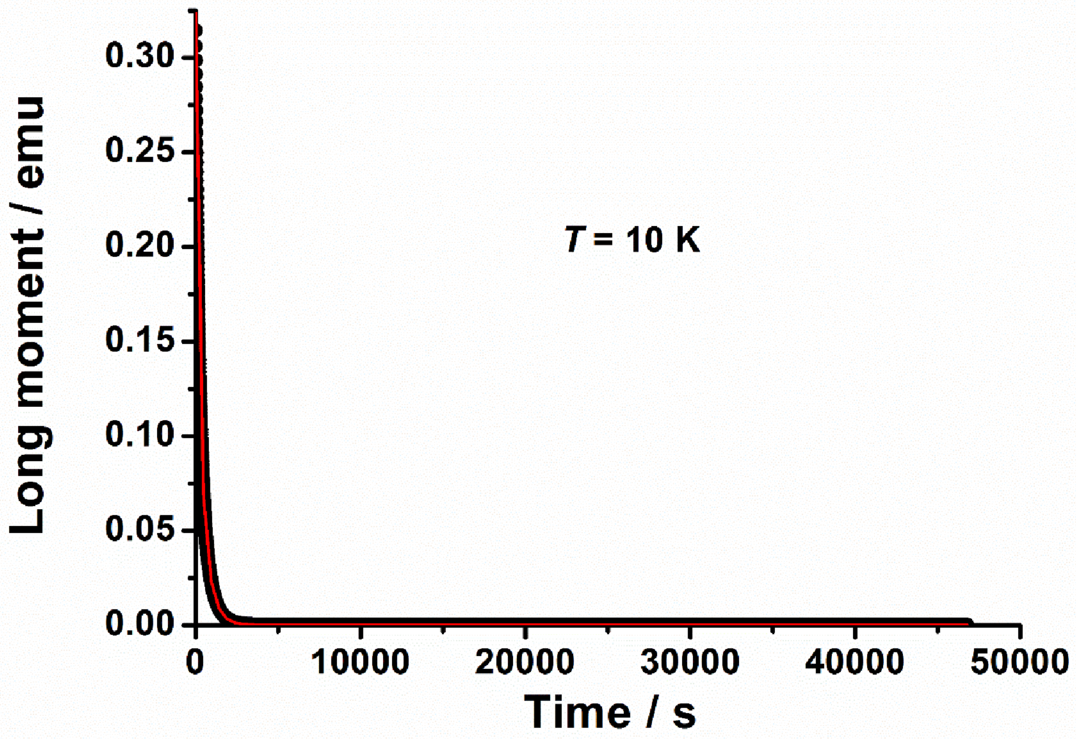

Figure S43. Plot of magnetization decay *vs.* time used to derive relaxation times for [**2**][$B(C_6F_5)_4$] at 10 K.

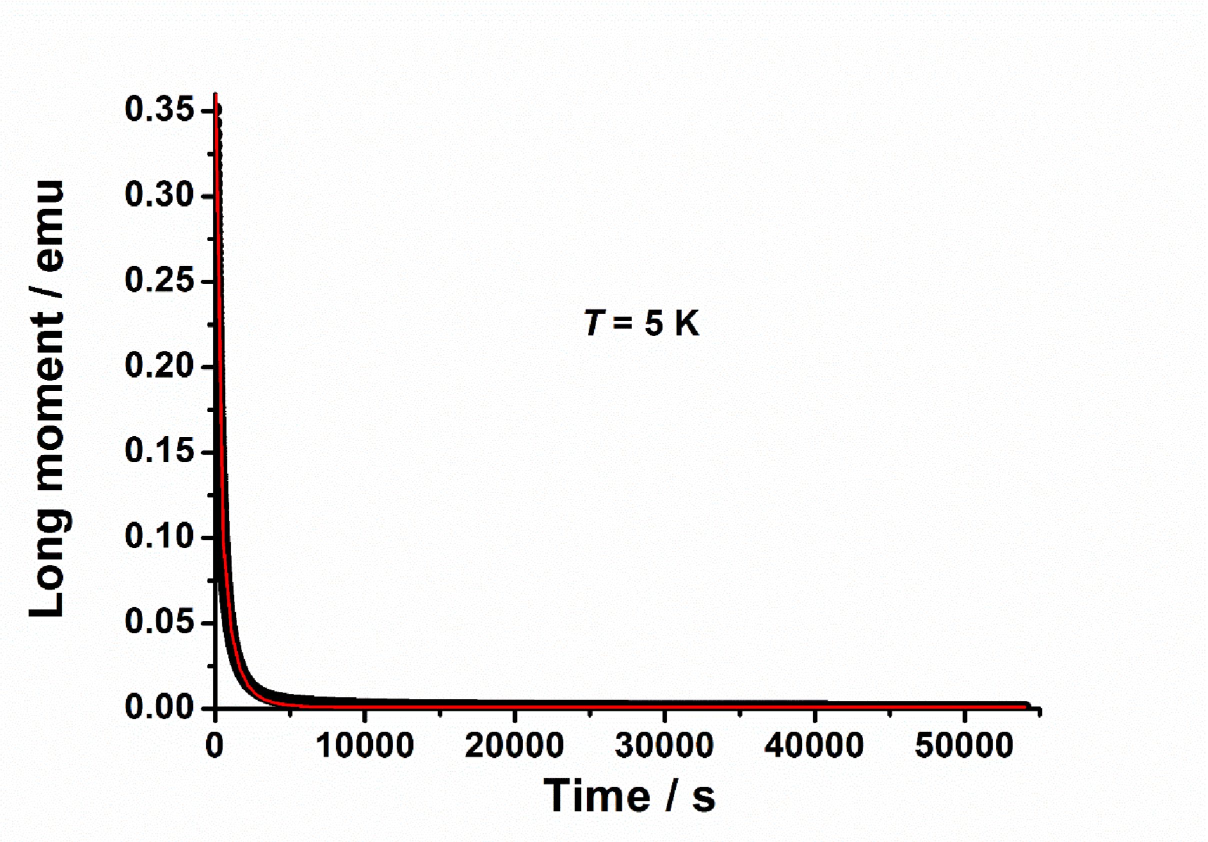

Figure S44. Plot of magnetization decay *vs.* time used to derive relaxation times for [**2**][B(C_6F_5)$_4$] at 5 K.

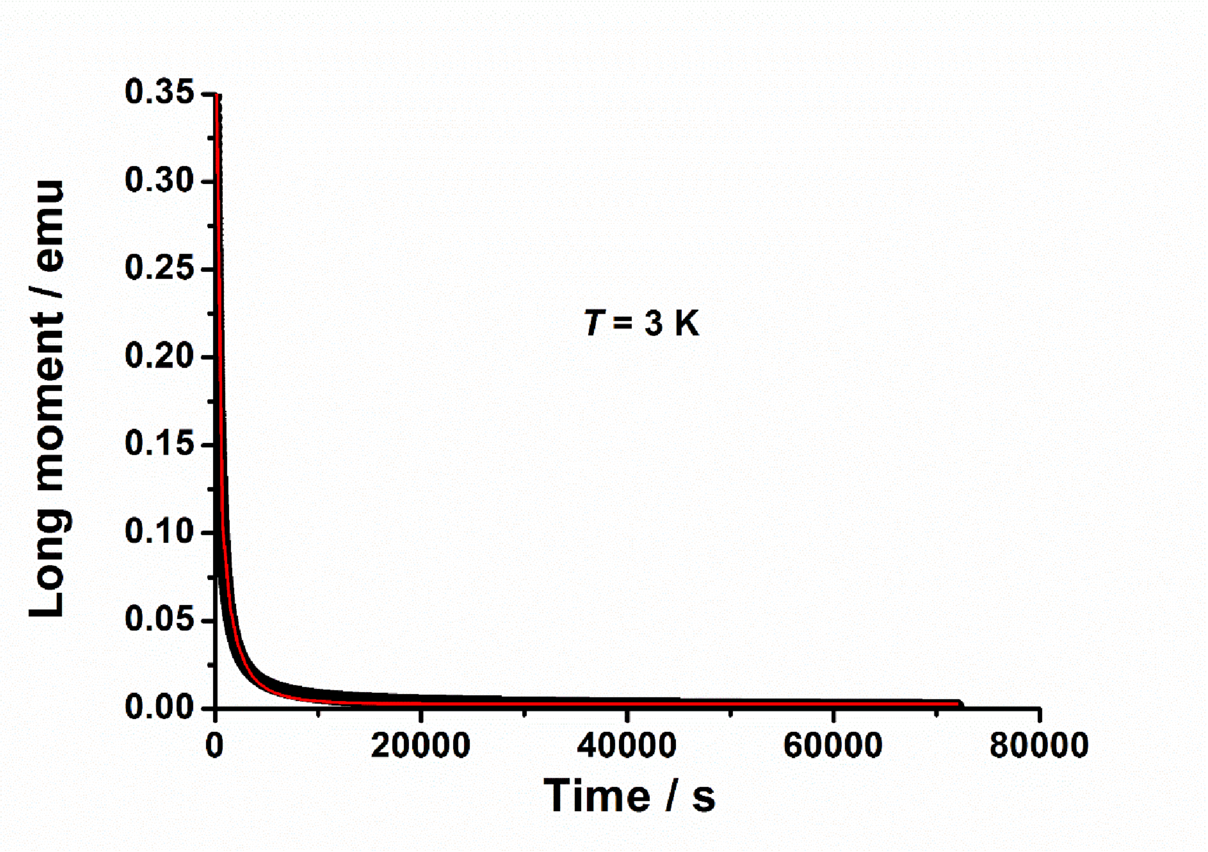

Figure S45. Plot of magnetization decay *vs.* time used to derive relaxation times for [**2**][$B(C_6F_5)_4$] at 3 K.

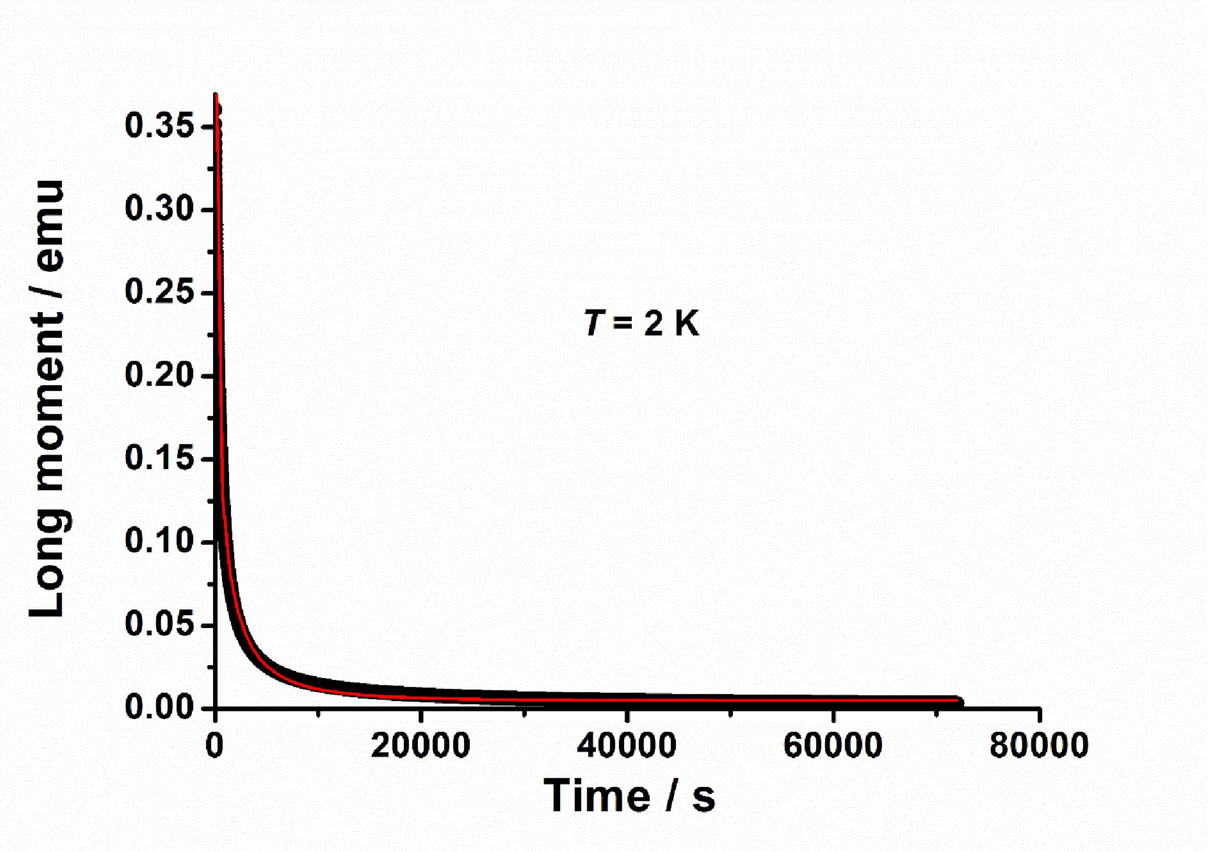

Figure S46. Plot of magnetization decay *vs.* time used to derive relaxation times for [**2**][$B(C_6F_5)_4$] at 2 K.

Table S9. Fitting parameters (initial magnetization (M_0), final magnetization (M_f), relaxation time (τ), and generalized coefficient (β)) for [**2**][$B(C_6F_5)_4$] at different temperatures from the least-squares fitting to the exponential decay as $M(t) = M_f + (M_0 - M_f)\exp[(-(t/\tau)^{\beta}]$, as shown in Figures. S28-S44.

***T* / K**	***M*f**	***M0***	**τ / s**	**β**
75	3.55283E-5	0.0022	1.9752	0.99946
70	3.8408E-5	0.01106	6.37419	0.98226
65	4.48027E-5	0.02438	13.22759	0.95965
60	4.44546E-5	0.03785	19.06457	0.94417
55	5.49101E-5	0.04865	24.236	0.93246
50	5.98953E-5	0.06243	30.10814	0.92496
45	6.71075E-5	0.08074	37.30459	0.9136
40	8.346E-5	0.10137	46.68922	0.90044
35	5.12631E-5	0.13095	58.98712	0.88591
30	6.23018E-5	0.16378	76.39537	0.87384
25	8.52658E-5	0.20873	100.03107	0.85543
20	1.03671E-4	0.25732	136.58272	0.84349
15	1.58857E-4	0.26074	190.96602	0.83432
10	7.09997E-5	0.3233	272.28663	0.77929
5	9.97628E-4	0.38353	338.14462	0.65582
3	0.00276	0.41586	384.68607	0.52618
2	0.00505	0.45148	412.43879	0.44874

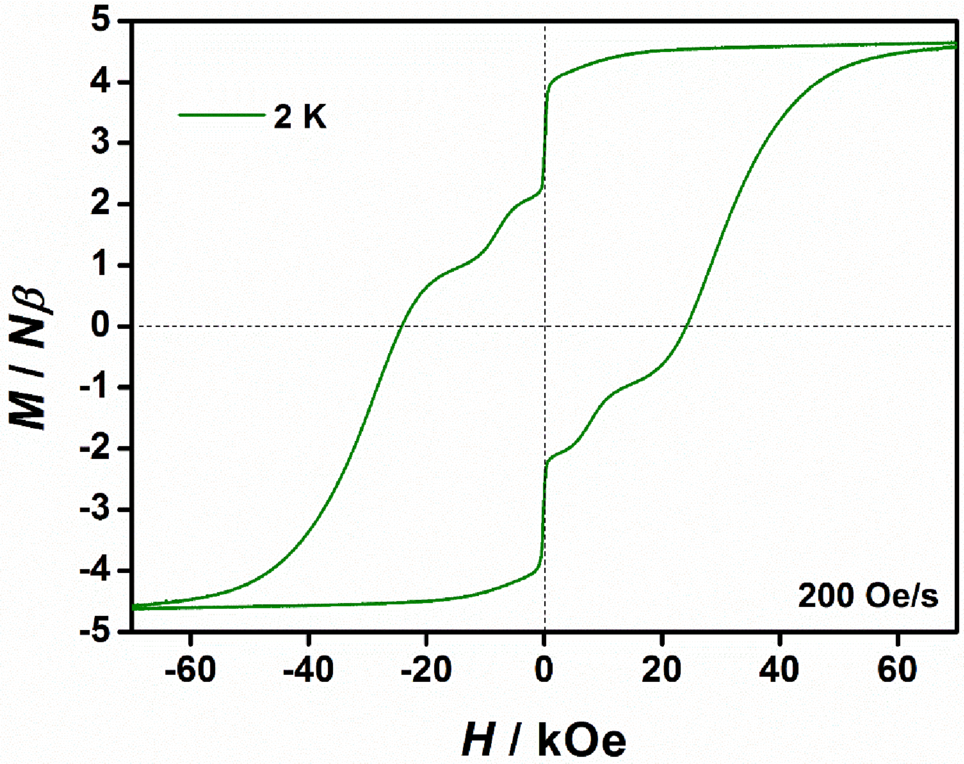

Figure S47. Magnetic hysteresis loops for [**2**][B(C_6F_5)$_4$]. The data were continuously collected at 2 K with a field sweep rate of 200 Oe s^{-1}.

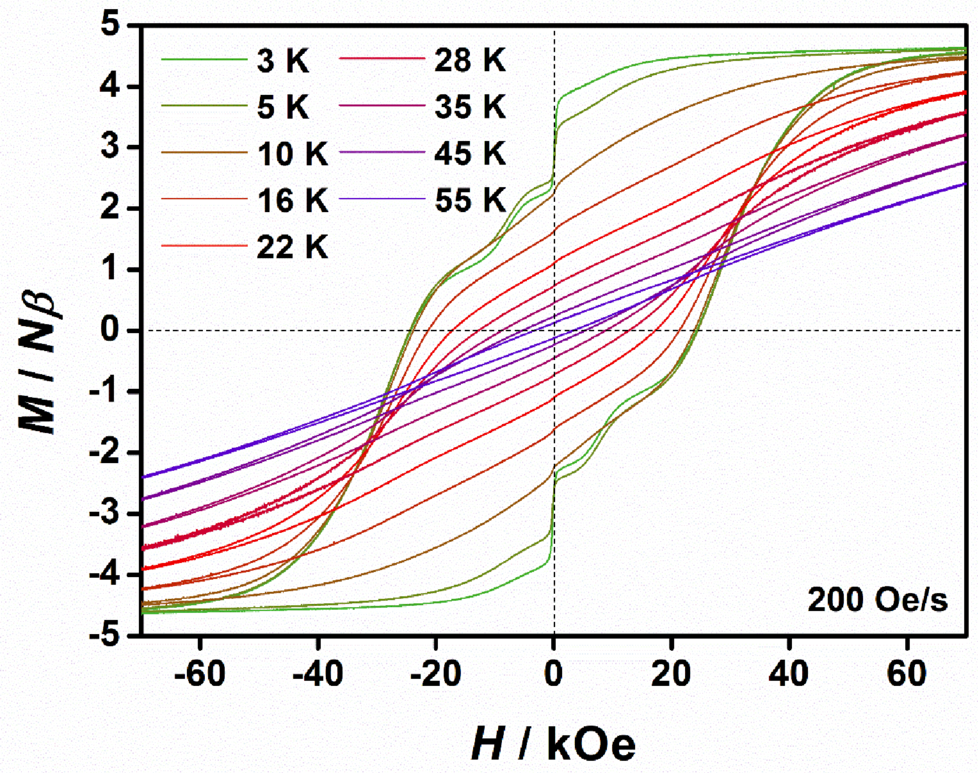

Figure S48. Magnetic hysteresis loops for [**2**][B(C_6F_5)$_4$]. The data were continuously collected at 2 K with a field sweep rate of 200 Oe s^{-1}.

Table S10. Coercive fields for [**2**][$B(C_6F_5)_4$] at different temperatures at sweep rate of 200 Oe s^{-1}.

Temperature / K	Coercive field / Oe
2	24169
3	24288
4	24369
5	24410
7	24386
10	23847
13	22827
16	21308
19	19448
22	17190
25	14968
28	12752
31	10869
35	8828
40	6810
45	5329
50	4209
55	3288
60	2548
65	1729
70	776
75	269

Table S11. Coercive fields for [**2**][$B(C_6F_5)_4$] at different temperatures at sweep rate of 25 Oe s^{-1}, corresponding to Figure 6 in the main text.

Temperature / K	Coercive field / Oe
60	277
62	237
64	196
66	151
68	112
70	63

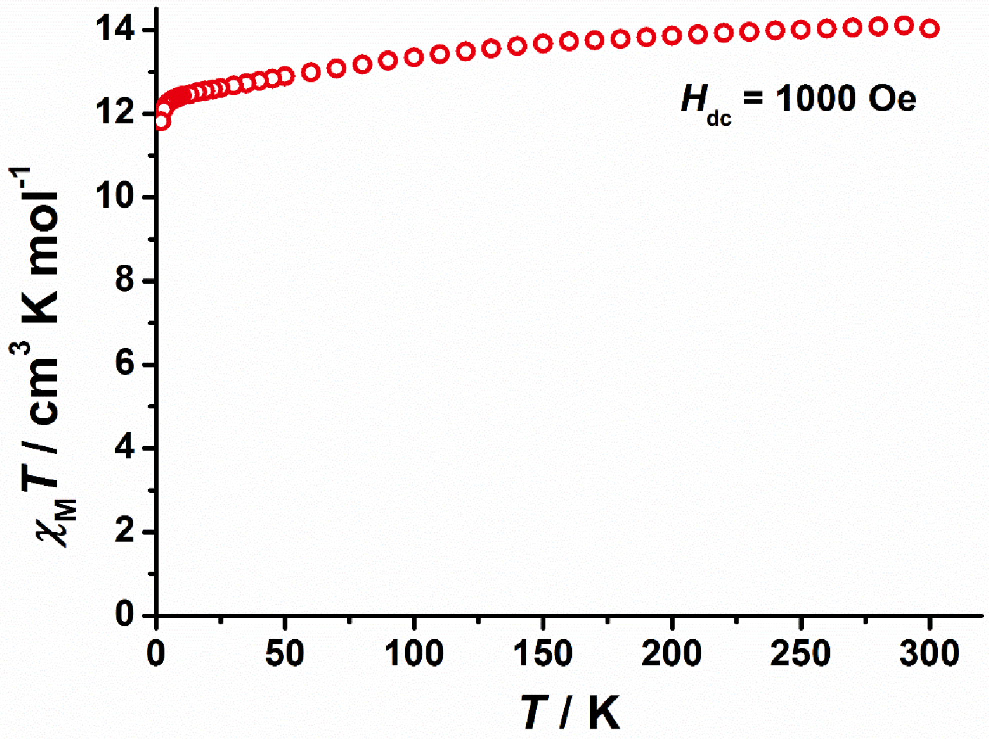

Figure S49. Plot of $\chi_M T$ versus temperature for [(Cp^{iPr5})Dy(Cp^{Et4P})(BH_4)] in an applied magnetic field of 1 kOe. $\chi_M T$(300 K) = 14.03 cm^3 K mol^{-1}, $\chi_M T$(2 K) = 11.82 cm^3 K mol^{-1}.

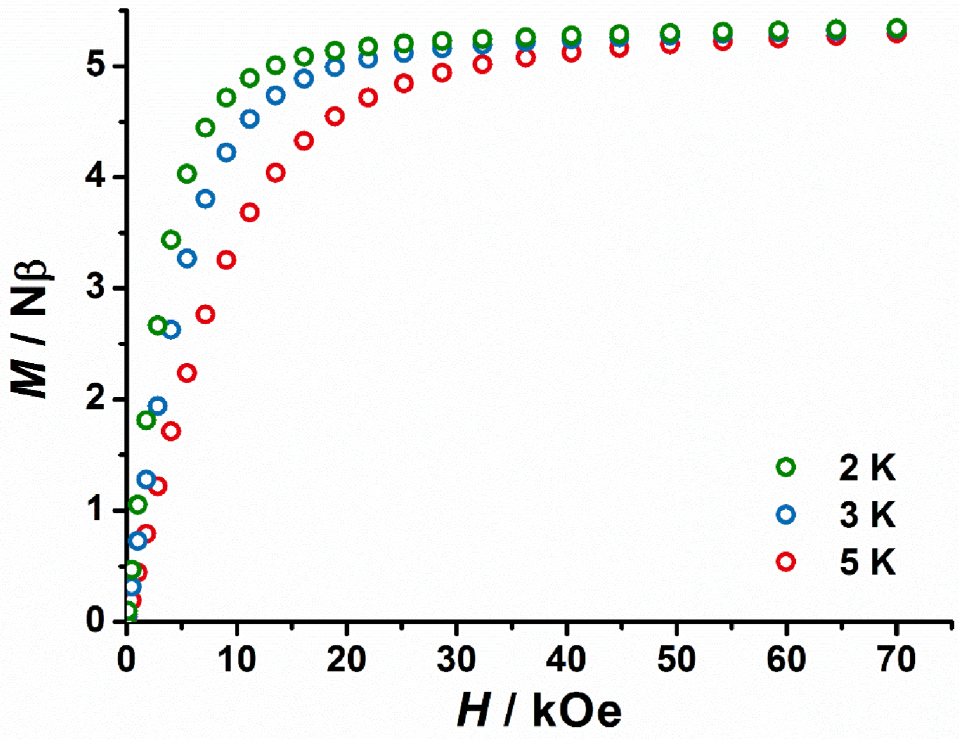

Figure S50. Field dependence of the magnetization (*M*) at 2 K, 3 K and 5 K for $[(Cp^{iPr5})Dy(Cp^{Et4P})(BH_4)]$. *M* = 5.34 Nβ at 2 K and 7 T.

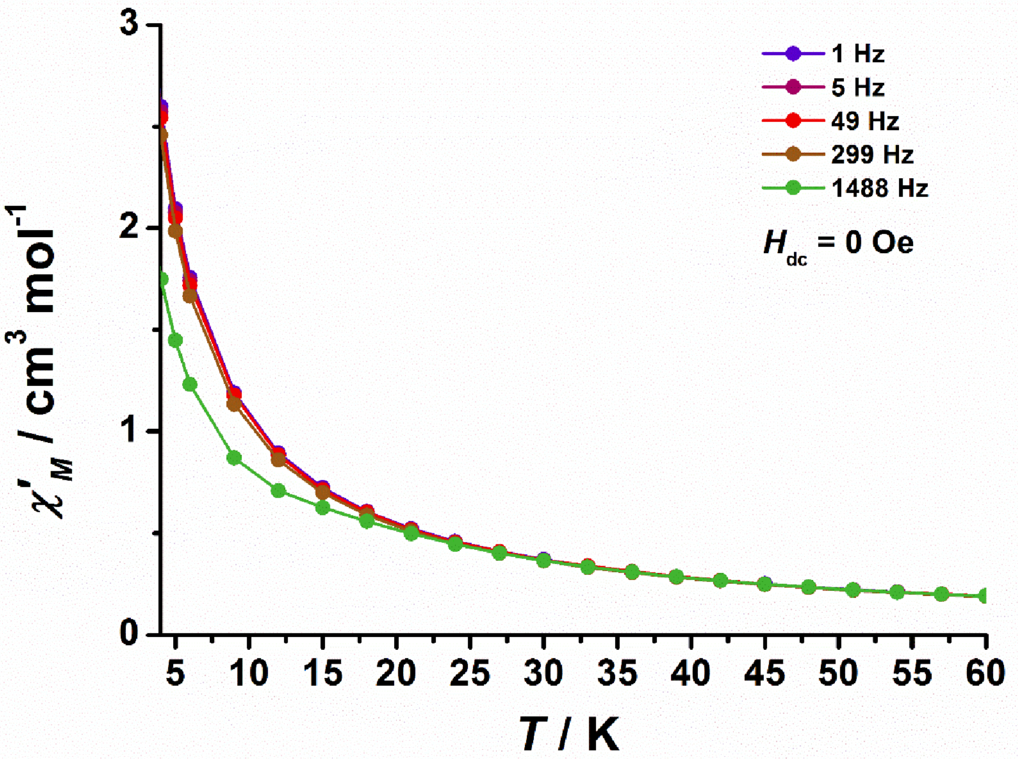

Figure S51. Temperature dependence of the in-phase susceptibility (χ'_M) for [(Cp^{iPr5})Dy(Cp^{Et4P})(BH_4)] in zero DC field at AC frequencies of 1-1488 Hz from 4 to 60 K. Solid lines are a guide to the eye.

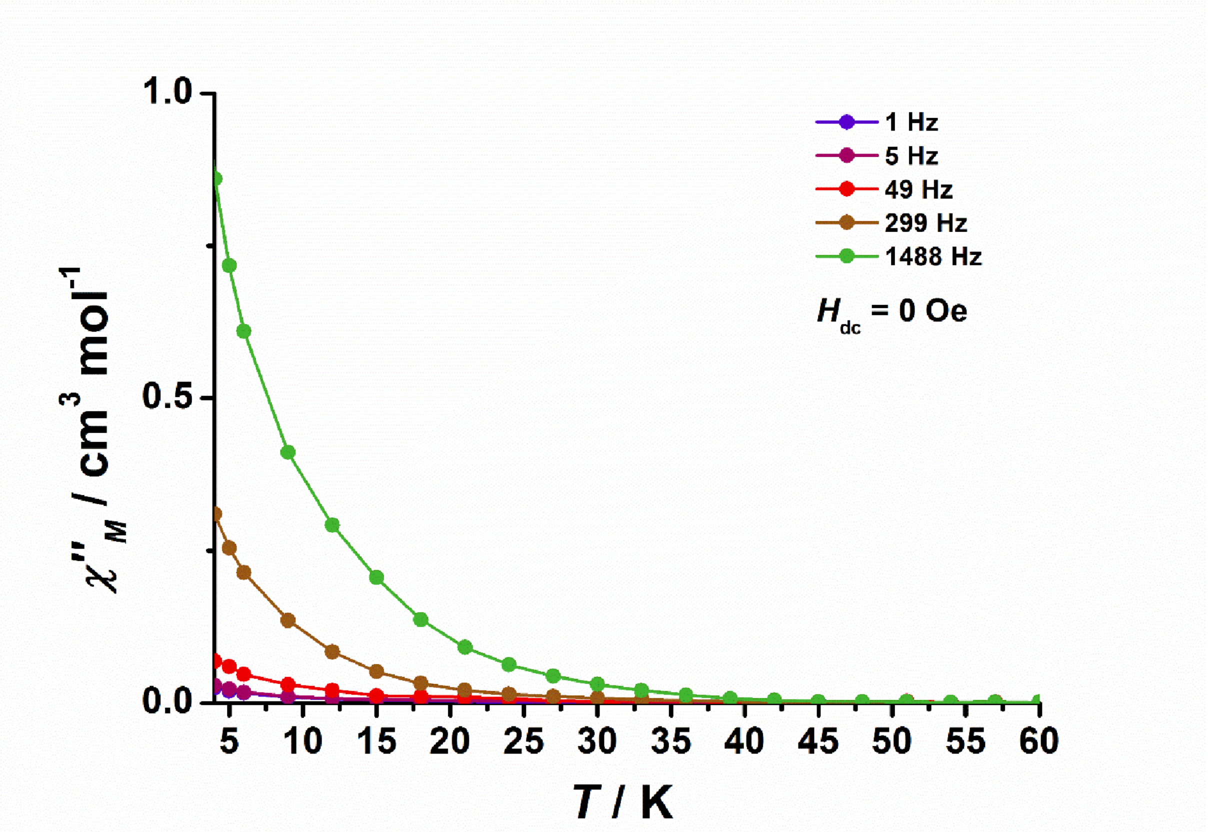

Figure S52. Temperature dependence of the out of phase susceptibility (χ''_M) for [(Cp^{iPr5})Dy(Cp^{Et4P})(BH_4)] in zero DC field at AC frequencies of 1-1488 Hz from 4 to 60 K. Solid lines are a guide to the eye.

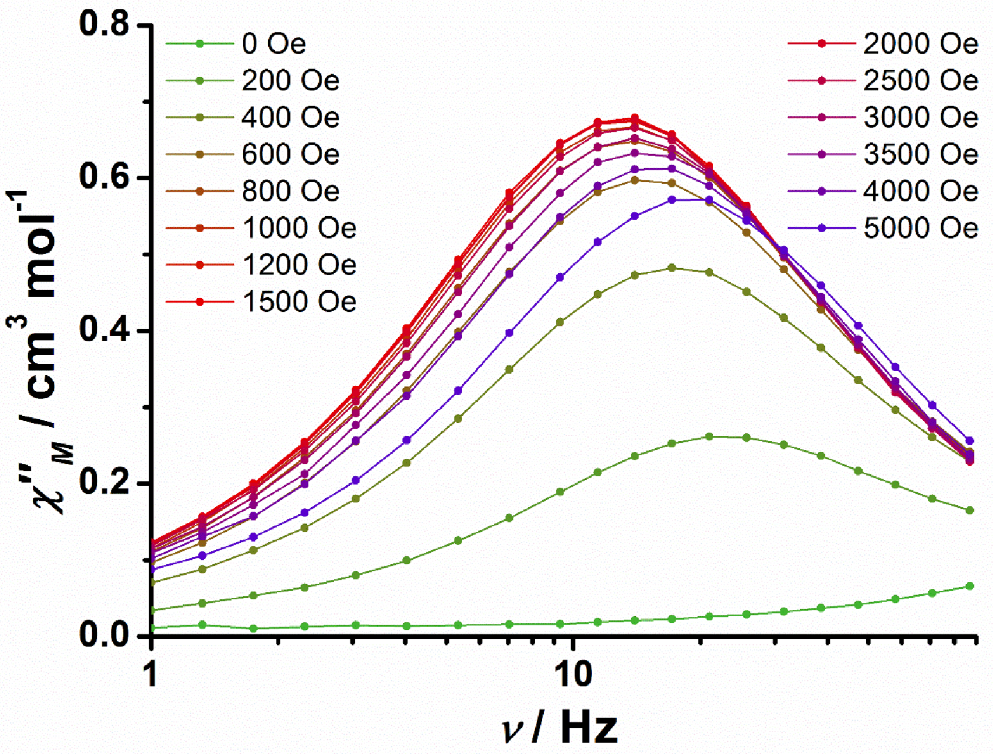

Figure S53. Frequency dependence of the out-of-phase (χ") AC susceptibility components at 7 K in various DC fields for [(Cp^{iPr5})Dy(Cp^{Et4P})(BH_4)].

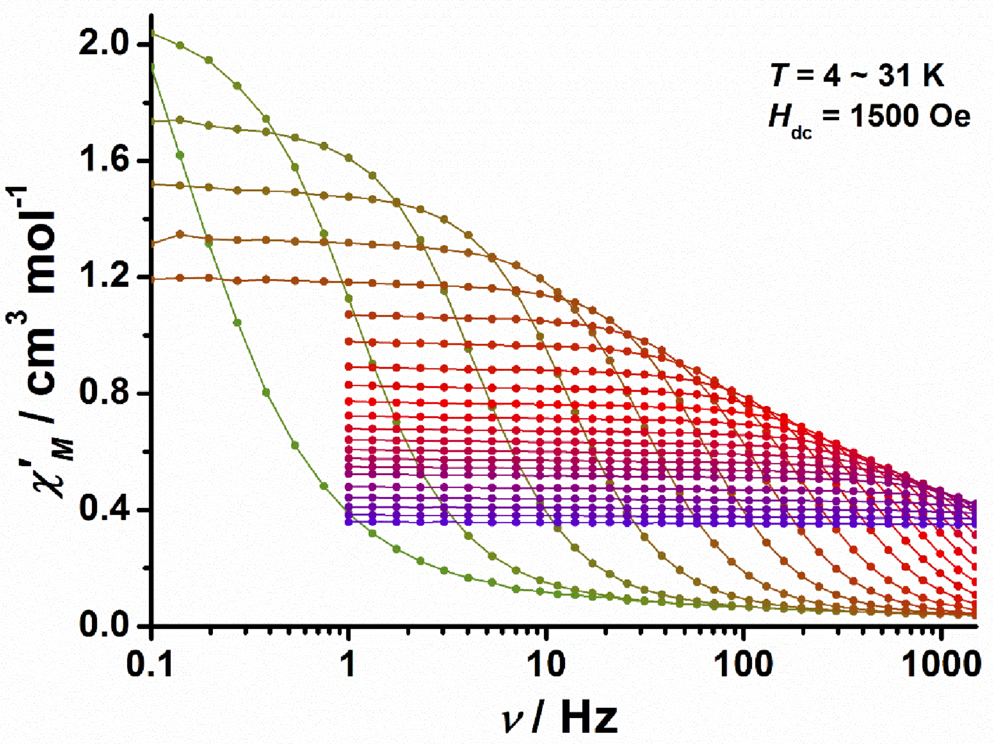

Figure S54. Frequency dependence of the in-phase (χ'_M) AC susceptibility component for $[(Cp^{iPr5})Dy(Cp^{Et4P})(BH_4)]$ in a DC field of 1500 Oe at AC frequencies of 1-1488 Hz from 4 to 31 K. Solid lines are a guide to the eye.

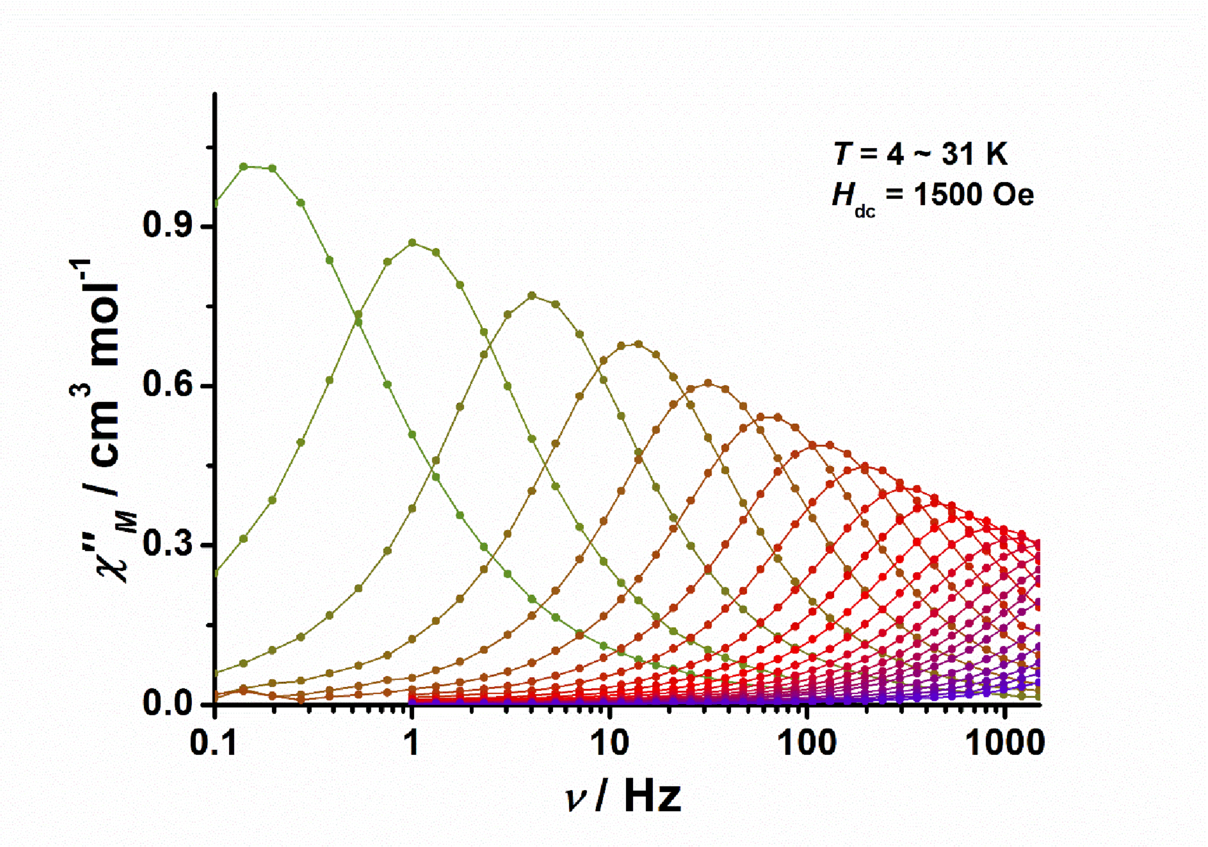

Figure S55. Frequency dependence of the out-of-phase (χ”) AC susceptibility components for [(Cp^{iPr5})Dy(Cp^{Et4P})(BH_4)] in a DC field of 1500 Oe at AC frequencies of 1-1488 Hz from 4 to 31 K. Solid lines are a guide to the eye.

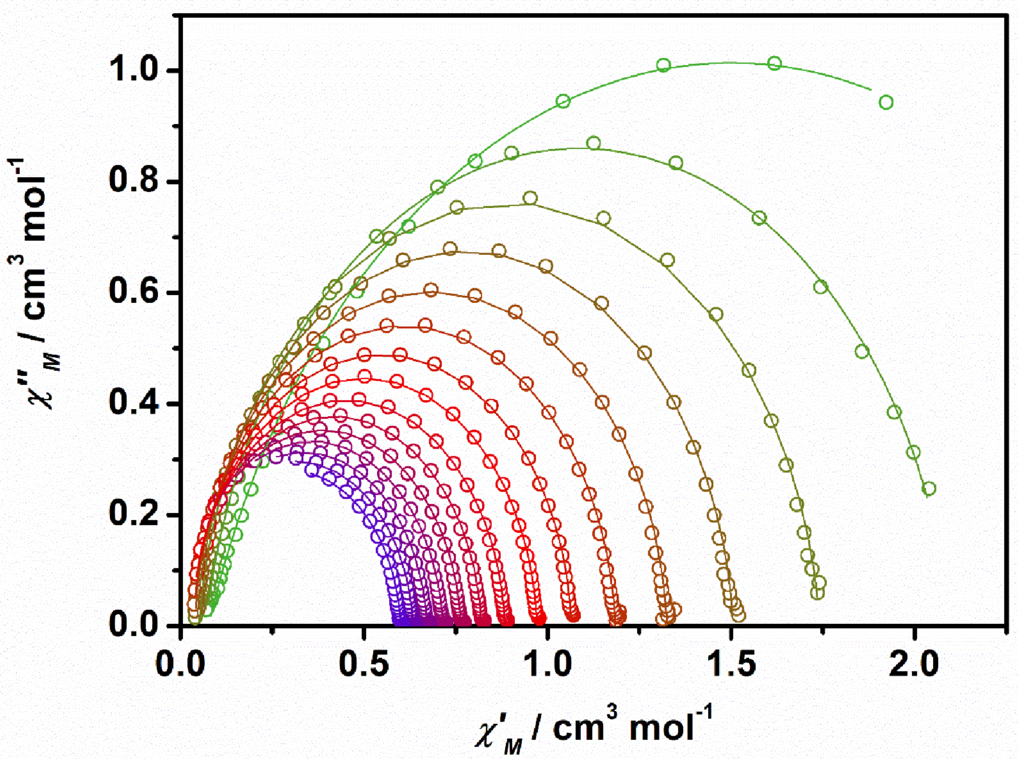

Figure S56. Cole-Cole plots for the AC susceptibilities in DC field of 1500 Oe for $[(Cp^{iPr5})Dy(Cp^{Et4P})(BH_4)]$ from 4-18 K. Solid lines represent fits to the data.

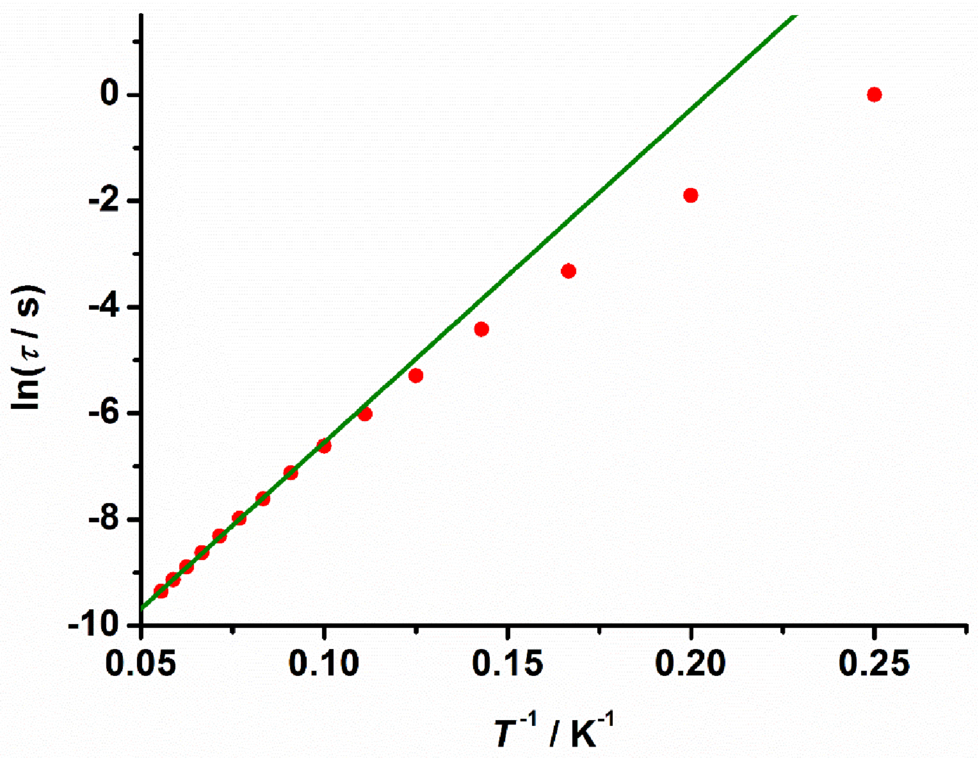

Figure S57. Plot of natural log of the relaxation time *vs.* inverse temperature for $[(Cp^{iPr5})Dy(Cp^{Et4P})(BH_4)]$. The red points are from the AC susceptibility measurements. The solid green line is the best linear Arrhenius fit, giving: U_{eff} = 43 cm^{-1}, τ_0 = 2.7 × 10^{-6} s.

Computational details

Two different types of geometry optimizations were carried out: a full optimization of the cation **2** and an optimization of the positions of the hydrogen atoms with the coordinates of heavier atoms frozen to their crystal-structure positions of the cations **2** and **3**. Both the major and minor disordered components of **2** and **3** were considered in the calculations. We will refer to the structures of the major components with the optimized hydrogen positions as **2a** and **3a**, and to the minor components as **2b** and **3b** when distinction between the structures is computationally relevant. The geometry optimizations were carried out using density functional theory (DFT).

The DFT calculations were conducted using the ADF 2019 software[1]. Scalar relativistic effects were accounted for with the zeroth order regular approximation (ZORA).[2] The standard Slater-type basis sets designed for ZORA calculations were used throughout.[3] A valence triple-ζ quality basis set with two sets of polarization functions (TZ2P) was used for the Dy^{3+} ion, valence triple-ζ quality basis set with a single set of polarization functions (TZP) was used for the P atoms and the C atoms in the Cp rings, and a valence double- ζ quality basis with a single set of polarization functions was used for the remaining C atoms and the H atoms.

The geometry optimization were carried out using the pure PBE exchange-correlation (XC) functional[4] and Grimme's DFT-D3 empirical dispersion correction[5] in conjunction with the Becke–Johnson (BS) damping function[6]. The "NumericalQuality" keyword in ADF was set to "Good" to avoid convergence issues due to numerical noise. To simulate the static correlation effects within the open 4f shell, the two unpaired β electrons were equally distributed over the seven 4f orbitals to yield non-integer occupation numbers. The geometry convergence tolerances were increased to 10^{-4}, 10^{-4}, 10^{-3} and 10^{-1} atomic units for the energy, gradient, bond length step size and bond angle step size, respectively. In case of the full optimization, a further frequency calculation was carried out to ensure that the stationary geometry corresponded to a minimum on the potential energy surface and to provide the normal modes needed for the spin-phonon calculations (*vide infra*). To smoothen convergence and to simulate static electron correlation effects, the nine 4f electrons were equally distributed over the seven 4f orbitals. In practice this means, that the occupations of the seven highest-energy β orbitals are fixed to the fractional values 2/7 ≈ 0.285714.

The hyperfine coupling tensors were calculated using geometry **2a**. The calculation was carried out using the PBE0 hybrid XC functional.[4,7] The basis sets of the target atoms (P and Dy) were augmented to TZ2P-J.[8] The TZ2P-J basis corresponds to the TZ2P basis but has additional steep *s* functions to better account for the electron density near the nucleus. The "NumericalAccuracy" keyword in ADF was increased to "VeryGood". The hyperfine coupling tensors were calculated using the ZORA methodology implemented in ADF with perturbative treatment of spin-orbit coupling (SOC) effects.[9]

The magnetic properties were calculated using multireference electron correlation methods as implemented in the *Molcas* quantum chemistry software version 8.4[10] and *OpenMolcas* version 21.02[11]. The static properties on structures **2a**, **2b**, **3a** and **3b** were calculated using OpenMolcas whereas the spin-phonon coupling constants were calculated with *Molcas*. First, a set of state-averaged (SA) complete active space self-consistent field (CASSCF) calculations[12] were carried out. The active space consisted of the nine 4f electrons in the seven 4f orbitals. All 21 sextet, 224 quartet and 490 doublet states were solved in three separate state-averaged calculations. Spin-orbit coupling (SOC) was accounted for using the restricted active space state-interaction (SO-RASSI) approach[13] where the SOC operator was constructed in a basis of the CASSCF eigenstates. All SA-CASSCF states up to an energy-cutoff of 50,000 cm^{-1} were included in the SO-RASSI treatment; this set of states consisted of all 21 sextet, lowest 128 quartet and lowest 130 doublet states. The static magnetic properties, the *ab initio* CF decomposition and the effective relaxation barrier were calculated using the SINGLE_ANISO routine in *Molcas* and *OpenMolcas*.[14,15] Scalar relativistic effects were included using the scalar version of the exact two-component (X2C) transformation as implemented in *Molcas*.[16] The atomic mean-field integral (AMFI) formalism was used in the construction of the SOC operator.[17] Relativistic atomic natural orbital (ANO-RCC) basis sets were used in all multireference calculations.[18] A polarized

valence quadruple-ζ basis set (VQZP) was used for the Dy ion, polarized valence triple-ζ basis sets (VTZP) were used for the P atom and the C atoms in the Cp rings, whereas a polarized double-ζ basis sets (VDZP) were used for the remaining C atoms and a simple double-ζ basis (VDZ) was used for the remaining H atoms. Cholesky decomposition with a threshold of 10^{-8} atomic units was used to reduce the necessary storage space for the two-electron integrals.

The spin-phonon coupling constants V_{IJ}^{α} of phonon α between CF eigenstates I and J were calculated as the matrix elements of the derivative of the CF operator $\hat{H}_{CF}$ with respect to unitless normal modes Q_{α}:

$$V_{IJ}^{\alpha} = \left\langle \psi_I \middle| \frac{\partial \hat{H}_{CF}}{\partial Q_{\alpha}} \middle| \psi_J \right\rangle.$$

From the Hermicity of $\hat{H}_{CF}$ it follows that $V_{IJ}^{\alpha} \vee V_{JI}^{\alpha} \vee$. The CF operator is written in terms of the equivalent operators $\hat{O}_{kq}(\hat{J})$ of rank *k* and component *q*[19]

$$\hat{H}_{CF} = \sum_{k,q} B_{kq} \hat{O}_{kq}(\hat{J}),$$

where B_{kq} is a complex CF parameter. The rank *k* has even, non-negative values. The largest value of *k* is 2*J*. The components range in integer steps from *q* = –*k* to *k*. The equivalent operators are used in the Iwahara–Chibotaru notation[20], where the matrix element between angular momentum eigenstates is given by a ratio of two Clebsch–Gordan coefficients:

$$\langle JM | \hat{O}_{kq}(\hat{J}) | JM' \rangle = \frac{C_{JM',kq}^{JM}}{C_{JJ,k0}^{JJ}}.$$

The phonons were approximated as the gas-phase normal modes of the molecule. This approximation neglects both phonon dispersion as well as all acoustic modes. The spin-phonon coupling constants were constructed from Cartesian derivatives of the CF operator by[21]

$$V_{IJ}^{\alpha} = \sum_i L_{i\alpha} \sqrt{\frac{\hbar}{\omega_{\alpha} m_i}} \sum_{k,q} \left(\frac{\partial B_{kq}}{\partial \xi_i} \right)_0 \sum_{M,M'} A_{MI} A_{M'J} \langle JM | \hat{O}_{kq}(\hat{J}) | JM' \rangle,$$

where the index *i* runs over Cartesian displacements ξ_i of all atoms, $L_{i\alpha}$ are the normalized eigenvectors of the Hessian in mass-weighted coordinates, ω_{α} is the angular frequency of normal mode α, m_i is the atomic mass of the atom displaced in displacement ξ_i, the indices *M* and *M'* run over the projections of the angular momentum *J*, A_{MI} are the CF eigenvectors.

The normal modes were determined from the full DFT optimization of **2**. The Cartesian derivatives of the CF parameters were calculated by conducting a series of multireference calculations on geometries displaced along each Cartesian displacement direction. Each atom was displaced in five steps of 0.001 Å in positive and negative directions along each three Cartesian coordinates. Thus, a totality of 2671 displacements (including the equilibrium structure) were calculated. The large number of calculations necessitated a reduction in the level of theory as compared to the calculation of the static magnetic properties. Only the 21 sextet states were considered in the SA-CASSCF and SO-RASSI calculations and electron correlation effects outside the 4*f* orbital space were neglected. A VTZP basis was used for the Dy^{3+} ion, VDZP basis sets were used for the P atom and the C atoms in the Cp rings and a valence double-ζ basis (VDZ) sets were used for the remaining C atoms and the H atoms. The optimization of the equilibrium geometry introduces some deviation between the static magnetic properties calculated for the crystal-structure geometry and for the optimized equilibrium geometry, although all considered properties are qualitatively similar. To keep the main text consistent, the energies of the KDs are discussed in terms of the values calculated for the crystal structure geometry.

The CF parameters were calculated for each displaced geometry using the *ab initio* CF theory.[15] The Cartesian derivatives of the parameters were extracted by conducting a linear fit on each parameter using the ten displaced geometries and the equilibrium geometry. The complex CF parameters are only determined up to a phase, which varies between the calculations on different displaced geometries. To eliminate the phase-dependence of the results, the phase of each CF parameter was fixed to that in the equilibrium geometry and only the magnitudes of the parameters were varied in the fit. The effect of this approximation on the quality of the results was estimated by diagonalizing the CF operator in each displaced geometry with and without the phase-shift and comparing the results. In each 2671 geometries both the maximum and mean error in each eigenvalue was less than 1 cm^{-1}. Thus, the effect of the phase-approximation on the results can be considered negligible. The numerical stability of the fits was studied by examining the coefficient of determination of each fit. In the case of large derivatives, the coefficient was usually larger than 0.98. However, in the case of small derivatives very low values of some coefficients was observed. To eliminate derivatives which could not be distinguished from numerical noise, all Cartesian derivatives with absolute values smaller than 1 cm^{-1} Å^{-1} were removed from the results. This necessarily introduces some error to the quantitative values of the spin-phonon coupling constants. However, the values of coupling constants with absolute values considerably smaller than 1 cm^{-1} still largely depend on the Cartesian derivatives with values larger than 1 cm^{-1} Å^{-1}; thus, the qualitative picture remains the same.

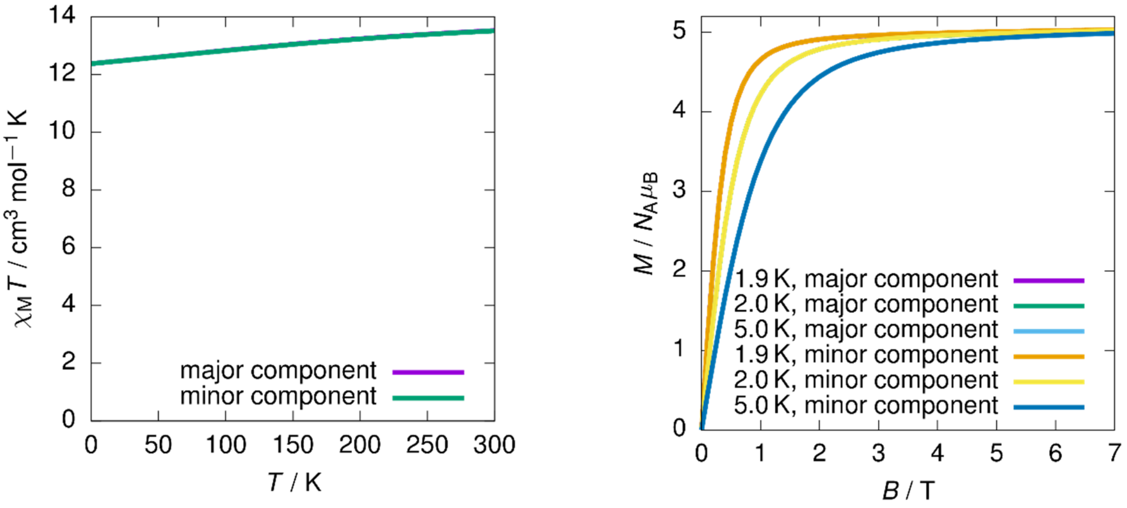

Figure S58. Calculated $\chi_M T(T)$ (1000 Oe DC field) and $M(H)$ data for **2a** and **2b**.

Table S12. Ab initio crystal-field parameters (in cm^{-1}) calculated for **2a** listed using the Iwahara–Chibotaru notation.[31]

k	***q***	**Re(*Bkq*)**	**Im(*Bkq*)**	**|*Bkq*|**
2	0	−868.227139	0.000000	868.227139
2	1	5.061376	−10.456741	11.617270
2	2	31.545291	27.346444	41.748454
4	0	−41.513038	−0.000000	41.513038
4	1	0.323129	0.880762	0.938166
4	2	−6.616396	−7.430522	9.949339
4	3	−1.883565	−0.216867	1.896008
4	4	4.302991	3.907587	5.812484
6	0	−51.584044	0.000000	51.584044
6	1	−2.332297	3.695986	4.370346
6	2	6.353888	7.014266	9.464239
6	3	1.234049	−0.716497	1.426971
6	4	2.404711	2.390912	3.391031
6	5	1.538731	1.577775	2.203876
6	6	−0.470872	1.588155	1.656489
8	0	1.823605	−0.000000	1.823605
8	1	0.135020	−0.204844	0.245339
8	2	−0.368489	−0.382945	0.531443
8	3	−0.040501	0.020944	0.045596
8	4	−0.061663	−0.079992	0.101000
8	5	−0.036828	−0.030330	0.047709
8	6	0.001548	−0.009378	0.009505
8	7	−0.000095	0.002507	0.002509
8	8	−0.001227	−0.000678	0.001401
10	0	0.079436	−0.000000	0.079436
10	1	0.002696	−0.009750	0.010116
10	2	0.004183	0.000873	0.004273
10	3	0.000124	0.001595	0.001600
10	4	−0.006898	−0.008527	0.010968
10	5	−0.003064	−0.003519	0.004666
10	6	0.000436	−0.002674	0.002710
10	7	−0.000268	−0.000163	0.000313
10	8	−0.000357	0.000313	0.000475
10	9	0.000066	0.000247	0.000256
10	10	−0.000377	0.000155	0.000407
12	0	0.007888	0.000000	0.007888
12	1	0.000988	−0.001339	0.001664
12	2	−0.002568	−0.002772	0.003779
12	3	−0.000454	0.000399	0.000604
12	4	−0.000399	0.000056	0.000403
12	5	0.000026	−0.000064	0.000069
12	6	0.000063	0.000010	0.000064

12	7	0.000047	0.000027	0.000054
12	8	0.000026	0.000018	0.000031
12	9	0.000001	0.000026	0.000026
12	10	−0.000007	0.000016	0.000017
12	11	−0.000012	0.000006	0.000014
12	12	−0.000006	−0.000004	0.000008
14	0	−0.000019	0.000000	0.000019
14	1	−0.000007	0.000005	0.000009
14	2	0.000018	0.000018	0.000026
14	3	0.000001	−0.000002	0.000002
14	4	0.000005	0.000005	0.000007
14	5	0.000003	0.000003	0.000004
14	6	−0.000000	0.000003	0.000003
14	7	0.000000	0.000000	0.000000
14	8	0.000000	−0.000000	0.000000
14	9	0.000000	−0.000000	0.000000
14	10	0.000000	−0.000000	0.000000
14	11	0.000000	−0.000000	0.000000
14	12	0.000000	0.000000	0.000000
14	13	0.000000	0.000000	0.000000
14	14	−0.000000	−0.000000	0.000000

a The CF parameters are only listed for non-negative values of *q*. The values with negative *q* are given by *Bk−q* = (−1)*qBkq**.

Table S13. Ab initio crystal-field parameters (in cm^{-1}) calculated for **2b** listed using the Iwahara–Chibotaru notation.[31]

k	***q***	**Re(*Bkq*)**	**Im(*Bkq*)**	**|*Bkq*|**
2	0	−889.492835	0.000000	889.492835
2	1	3.879029	14.665473	15.169804
2	2	2.370228	−49.596031	49.652637
4	0	−37.326588	−0.000000	37.326588
4	1	−6.712875	−4.990070	8.364418
4	2	3.243505	−6.220148	7.015024
4	3	−4.307976	3.578322	5.600272
4	4	−2.110193	3.783493	4.332174
6	0	−52.996172	−0.000000	52.996172
6	1	3.550491	−2.191329	4.172279
6	2	−5.422309	0.156772	5.424575
6	3	0.799638	−4.379689	4.452089
6	4	2.952585	−5.618559	6.347122
6	5	2.011088	−0.956246	2.226854
6	6	0.425539	−0.095043	0.436024
8	0	1.814500	−0.000000	1.814500
8	1	−0.196848	0.146244	0.245227
8	2	0.276264	0.069893	0.284968
8	3	−0.041968	0.241632	0.245250
8	4	−0.118700	0.215038	0.245624
8	5	−0.046081	0.018417	0.049625
8	6	−0.009060	−0.000281	0.009064
8	7	−0.001415	−0.000181	0.001427
8	8	−0.000650	0.000436	0.000783
10	0	0.085364	0.000000	0.085364
10	1	0.007636	0.013209	0.015257
10	2	0.001996	0.010284	0.010476
10	3	0.005647	−0.000613	0.005681
10	4	−0.004107	0.006853	0.007990
10	5	−0.004544	0.000127	0.004546
10	6	−0.000695	−0.000504	0.000859
10	7	0.000382	0.000970	0.001042
10	8	0.000268	0.001012	0.001047
10	9	−0.000048	0.000425	0.000428
10	10	−0.000040	0.000065	0.000076
12	0	0.007464	−0.000000	0.007464
12	1	−0.001719	0.001572	0.002329
12	2	0.002147	−0.000304	0.002168
12	3	−0.000629	0.001551	0.001674
12	4	−0.000407	0.000778	0.000878
12	5	−0.000099	0.000311	0.000326
12	6	−0.000136	0.000061	0.000149

12	7	−0.000094	−0.000104	0.000140
12	8	−0.000057	−0.000110	0.000124
12	9	0.000002	−0.000057	0.000057
12	10	0.000009	−0.000014	0.000016
12	11	0.000003	−0.000002	0.000004
12	12	0.000001	−0.000000	0.000001
14	0	−0.000012	−0.000000	0.000012
14	1	0.000013	−0.000005	0.000014
14	2	−0.000017	0.000004	0.000017
14	3	−0.000000	−0.000012	0.000012
14	4	0.000006	−0.000009	0.000011
14	5	0.000005	−0.000002	0.000005
14	6	0.000001	0.000000	0.000001
14	7	0.000000	0.000000	0.000000
14	8	0.000001	0.000001	0.000001
14	9	−0.000000	0.000000	0.000000
14	10	−0.000000	0.000000	0.000000
14	11	−0.000000	−0.000000	0.000000
14	12	−0.000000	−0.000000	0.000000
14	13	−0.000000	−0.000000	0.000000
14	14	−0.000000	−0.000000	0.000000

a The CF parameters are only listed for non-negative values of *q*. The values with negative *q* are given by *Bk−q* = (−1)*qBkq**.

Table S14. Decomposition of the wave functions calculated using the major component of the crystal structure of **2** (**2a**) corresponding to the eight lowest Kramers doublets (KDs) onto states with definite projections *M* of the pseudospin used in the construction of the *ab initio* crystal-field operator. The numbers quoted are the squared projections of the *ab initio* states onto the angular momentum states.

M	KD1		KD2		KD3		KD4		KD5		KD6		KD7		KD8	
−15/2	0.427	0.572	0.000	0.000	0.000	0.000	0.000	0.000	0.000	0.000	0.000	0.000	0.000	0.000	0.000	0.000
−13/2	0.000	0.000	0.069	0.929	0.000	0.002	0.000	0.001	0.000	0.000	0.000	0.000	0.000	0.000	0.000	0.000
−11/2	0.000	0.000	0.000	0.002	0.001	0.988	0.000	0.009	0.000	0.000	0.000	0.000	0.000	0.000	0.000	0.000
−9/2	0.000	0.000	0.000	0.001	0.000	0.008	0.000	0.985	0.000	0.004	0.000	0.001	0.000	0.000	0.000	0.000
−7/2	0.000	0.000	0.000	0.000	0.000	0.000	0.000	0.005	0.108	0.880	0.000	0.001	0.000	0.005	0.000	0.001
−5/2	0.000	0.000	0.000	0.000	0.000	0.000	0.000	0.001	0.000	0.001	0.022	0.942	0.014	0.000	0.013	0.006
−3/2	0.000	0.000	0.000	0.000	0.000	0.000	0.000	0.000	0.001	0.005	0.003	0.000	0.007	0.817	0.045	0.122
−1/2	0.000	0.000	0.000	0.000	0.000	0.000	0.000	0.000	0.000	0.000	0.001	0.030	0.154	0.002	0.601	0.211
1/2	0.000	0.000	0.000	0.000	0.000	0.000	0.000	0.000	0.000	0.000	0.030	0.001	0.002	0.154	0.211	0.601
3/2	0.000	0.000	0.000	0.000	0.000	0.000	0.000	0.000	0.005	0.001	0.000	0.003	0.817	0.007	0.122	0.045
5/2	0.000	0.000	0.000	0.000	0.000	0.000	0.001	0.000	0.001	0.000	0.942	0.022	0.000	0.014	0.006	0.013
7/2	0.000	0.000	0.000	0.000	0.000	0.000	0.005	0.000	0.880	0.108	0.001	0.000	0.005	0.000	0.001	0.000
9/2	0.000	0.000	0.001	0.000	0.008	0.000	0.985	0.000	0.004	0.000	0.001	0.000	0.000	0.000	0.000	0.000
11/2	0.000	0.000	0.002	0.000	0.988	0.001	0.009	0.000	0.000	0.000	0.000	0.000	0.000	0.000	0.000	0.000
13/2	0.000	0.000	0.929	0.069	0.002	0.000	0.001	0.000	0.000	0.000	0.000	0.000	0.000	0.000	0.000	0.000
15/2	0.572	0.427	0.000	0.000	0.000	0.000	0.000	0.000	0.000	0.000	0.000	0.000	0.000	0.000	0.000	0.000

Table S15. Decomposition of the wave functions calculated using the minor component of the crystal structure of **2** (**2b**) corresponding to the eight lowest Kramers doublets (KDs) onto states with definite projections *M* of the pseudospin used in the construction of the *ab initio* crystal-field operator. The numbers quoted are the squared projections of the *ab initio* states onto the angular momentum states.

M	KD1		KD2		KD3		KD4		KD5		KD6		KD7		KD8	
−15/2	0.299	0.701	0.000	0.000	0.000	0.000	0.000	0.000	0.000	0.000	0.000	0.000	0.000	0.000	0.000	0.000
−13/2	0.000	0.000	0.161	0.834	0.001	0.000	0.003	0.000	0.000	0.000	0.000	0.000	0.000	0.000	0.000	0.000
−11/2	0.000	0.000	0.000	0.001	0.959	0.021	0.010	0.000	0.000	0.008	0.000	0.000	0.000	0.000	0.000	0.000
−9/2	0.000	0.000	0.001	0.003	0.009	0.000	0.967	0.001	0.000	0.010	0.000	0.009	0.000	0.000	0.000	0.000
−7/2	0.000	0.000	0.000	0.000	0.009	0.000	0.008	0.000	0.017	0.948	0.000	0.008	0.006	0.003	0.001	0.001
−5/2	0.000	0.000	0.000	0.000	0.000	0.000	0.010	0.000	0.000	0.005	0.016	0.940	0.011	0.001	0.003	0.014
−3/2	0.000	0.000	0.000	0.000	0.000	0.000	0.001	0.000	0.000	0.010	0.004	0.006	0.625	0.250	0.093	0.012
−1/2	0.000	0.000	0.000	0.000	0.000	0.000	0.000	0.000	0.000	0.001	0.002	0.016	0.025	0.079	0.116	0.761
1/2	0.000	0.000	0.000	0.000	0.000	0.000	0.000	0.000	0.001	0.000	0.016	0.002	0.079	0.025	0.761	0.116
3/2	0.000	0.000	0.000	0.000	0.000	0.000	0.000	0.001	0.010	0.000	0.006	0.004	0.250	0.625	0.012	0.093
5/2	0.000	0.000	0.000	0.000	0.000	0.000	0.000	0.010	0.005	0.000	0.940	0.016	0.001	0.011	0.014	0.003
7/2	0.000	0.000	0.000	0.000	0.000	0.009	0.000	0.008	0.948	0.017	0.008	0.000	0.003	0.006	0.001	0.001
9/2	0.000	0.000	0.003	0.001	0.000	0.009	0.001	0.967	0.010	0.000	0.009	0.000	0.000	0.000	0.000	0.000
11/2	0.000	0.000	0.001	0.000	0.021	0.959	0.000	0.010	0.008	0.000	0.000	0.000	0.000	0.000	0.000	0.000
13/2	0.000	0.000	0.834	0.161	0.000	0.001	0.000	0.003	0.000	0.000	0.000	0.000	0.000	0.000	0.000	0.000
15/2	0.701	0.299	0.000	0.000	0.000	0.000	0.000	0.000	0.000	0.000	0.000	0.000	0.000	0.000	0.000	0.000

Table S16. Values of the transition magnetic moments (in Bohr magneton units) between the states in the eight lowest Kramers doublets (KDs) of **2a**.

Initial KD	Final KD	Climbing transition	Crossing transition
1	1	3.316782	0.000001
1	2	1.773428	0.000002
1	3	0.101155	0.000009
1	4	0.036114	0.000046
1	5	0.016271	0.000122
1	6	0.004963	0.000347
1	7	0.002737	0.000581
1	8	0.000527	0.000866
2	2	2.977834	0.000009
2	3	2.372449	0.000068
2	4	0.108840	0.000508
2	5	0.051592	0.000851
2	6	0.035269	0.002765
2	7	0.022744	0.004980
2	8	0.006608	0.007941
3	3	2.597674	0.001404
3	4	2.806993	0.001682
3	5	0.116977	0.004628
3	6	0.047126	0.004745
3	7	0.037157	0.018331
3	8	0.024956	0.023731
4	4	2.035588	0.009228
4	5	3.097958	0.016117
4	6	0.191681	0.060216
4	7	0.149601	0.023316
4	8	0.043139	0.037395
5	5	1.708221	0.130665
5	6	3.272481	0.094458
5	7	0.175630	0.292443
5	8	0.233925	0.065865
6	6	1.194928	0.470002
6	7	3.299662	0.175700
6	8	0.156709	0.838366
7	7	0.538954	2.573216
7	8	2.435672	0.428742
8	8	0.382646	3.830976

Table S17. Values of the transition magnetic moments (in Bohr magneton units) between the states in the eight lowest Kramers doublets (KDs) of **2b**.

Initial KD	Final KD	Climbing transition	Crossing transition
1	1	3.316671	0.000000
1	2	1.773681	0.000002
1	3	0.092586	0.000020
1	4	0.059098	0.000050
1	5	0.026215	0.000137
1	6	0.006959	0.000488
1	7	0.002218	0.000470
1	8	0.000499	0.000930
2	2	2.936122	0.000034
2	3	2.370933	0.000109
2	4	0.181216	0.000396
2	5	0.068288	0.000779
2	6	0.054788	0.002191
2	7	0.032128	0.003500
2	8	0.004168	0.007621
3	3	2.606484	0.000424
3	4	2.817343	0.001056
3	5	0.188001	0.004497
3	6	0.049647	0.002544
3	7	0.040251	0.020387
3	8	0.044246	0.007753
4	4	2.051494	0.008529
4	5	3.114517	0.010996
4	6	0.229695	0.028379
4	7	0.067960	0.017887
4	8	0.019972	0.074445
5	5	1.684389	0.068999
5	6	3.310906	0.055373
5	7	0.168545	0.140316
5	8	0.188967	0.054382
6	6	1.169539	0.486934
6	7	3.356491	0.278266
6	8	0.200201	0.699334
7	7	0.722647	2.129311
7	8	2.853678	0.444907
8	8	0.567124	3.921849

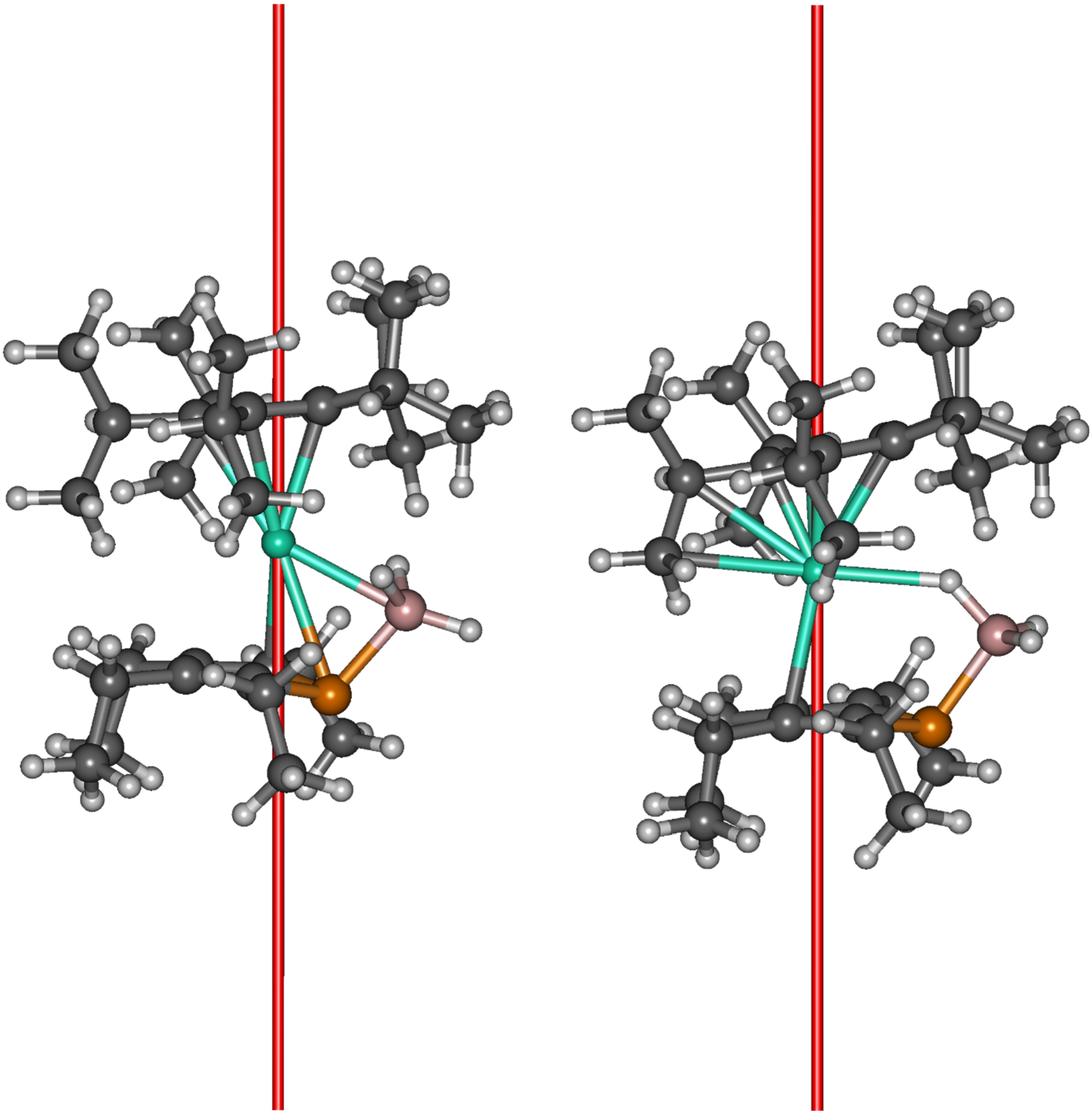

Figure S59. Principal magnetic axis of the ground Kramers doublet in **3a** (left) and **3b** (right). Green = Dy, orange = P, dark grey = C, pink = B, light grey = H.

Table S18. Energies and principal components of the g tensors of the eight lowest Kramers doublets of **3a** corresponding to the crystal-field split ground $^6H_{15/2}$ multiplet.

**KD**	***E*** **/ cm^{-1}**	g_x	g_y	g_z	θ^a
KD1	0	0.00064	0.00087	19.85733	
KD2	365	0.02798	0.03704	17.07395	167.2
KD3	521	0.13360	0.17277	14.55871	20.7
KD4	645	0.69973	0.94958	11.79266	176.6
KD5	748	1.38724	2.80092	9.20689	165.9
KD6	848	6.28006	5.53590	2.38159	107.7
KD7	933	1.75879	5.22869	12.85260	89.4
KD8	1140	0.07448	0.17403	19.30506	89.7

^{a} The angle between the principal magnetic axis of the given doublet and the that of the ground doublet.

Table S19. Energies and principal components of the **g** tensors of the eight lowest Kramers doublets of **3b** corresponding to the crystal-field split ground $^6H_{15/2}$ multiplet.

**KD**	***E*** **/ cm^{-1}**	g_x	g_y	g_z	θ^a
KD1	0	0.00010	0.00012	19.87564	
KD2	520	0.00443	0.00538	17.02460	172.4
KD3	779	0.05682	0.06036	14.39228	174.3
KD4	911	0.34792	0.44586	13.07364	27.1
KD5	1002	0.49297	1.25678	9.84737	30.0
KD6	1123	6.20997	4.37311	2.34667	103.1
KD7	1232	2.22868	6.38476	11.57777	89.9
KD8	1434	0.17029	0.47624	18.89621	90.6

^{a} The angle between the principal magnetic axis of the given doublet and the that of the ground doublet.

References

[1] F.-S. Guo, B. M. Day, Y.-C. Chen, M.-L. Tong, A. Mansikkamäki, R. A. Layfield, *Science.* **2018**, *362*, 1400–1403.

[2] X. Sava, L. Ricard, F. Mathey, P. Le Floch, *Organometallics* **2000**, *19*, 4899–4903.

[3] S. J. Connelly, W. Kaminsky, D. M. Heinekey, *Organometallics* **2013**, *32*, 7478–7481.

[4] O. V Dolomanov, L. J. Bourhis, R. J. Gildea, J. A. K. Howard, H. Puschmann, *J. Appl. Crystallogr.* **2009**, *42*, 339–341.

[5] G. M. Sheldrick, *Acta Crystallogr. Sect. C* **2015**, *71*, 3–8.

[6] G. M. Sheldrick, *Acta Crystallogr. Sect. A* **2008**, *64*, 112–122.

[7] G. te Velde, F. M. Bickelhaupt, E. J. Baerends, C. Fonseca Guerra, S. J. A. van Gisbergen, J. G. Snijders, T. Ziegler, *J. Comput. Chem.* **2001**, *22*, 931–967.

[8] C. Fonseca Guerra, J. G. Snijders, G. te Velde, E. J. Baerends, *Theor. Chem. Acc.* **1998**, *99*, 391–403.

[9] E. van van Lenthe, E. J. Baerends, J. G. Snijders, *J. Chem. Phys.* **1993**, *99*, 4597–4610.

[10] E. van Lenthe, E. J. Baerends, J. G. Snijders, *J. Chem. Phys.* **1994**, *101*, 9783–9792.

[11] E. van Lenthe, R. van Leeuwen, E. J. Baerends, J. G. Snijders, *Int. J. Quantum Chem.* **1996**, *57*, 281–293.

[12] E. van Lenthe, E. J. Baerends, *J. Comput. Chem.* **2003**, *24*, 1142–1156.

[13] J. P. Perdew, K. Burke, M. Ernzerhof, *Phys. Rev. Lett.* **1996**, *77*, 3865–3868.

[14] J. P. Perdew, K. Burke, M. Ernzerhof, *Phys. Rev. Lett.* **1997**, *78*, 1396.

[15] S. Grimme, J. Antony, S. Ehrlich, H. Krieg, *J. Chem. Phys.* **2010**, *132*, 154104.

[16] S. Grimme, S. Ehrlich, L. Goerigk, *J. Comput. Chem.* **2011**, *32*, 1456–1465.

[17] M. Ernzerhof, G. E. Scuseria, *J. Chem. Phys.* **1999**, *110*, 5029–5036.

[18] C. Adamo, V. Barone, *J. Chem. Phys.* **1999**, *110*, 6158–6170.

[19] S. Moncho, J. Autschbach, *J. Chem. Theory Comput.* **2010**, *6*, 223–234.

[20] J. Autschbach, S. Patchkovskii, B. Pritchard, *J. Chem. Theory Comput.* **2011**, *7*, 2175–2188.

[21] F. Aquilante, J. Autschbach, R. K. Carlson, L. F. Chibotaru, M. G. Delcey, L. De Vico, I. Fdez. Galván, N. Ferré, L. M. Frutos, L. Gagliardi, et al., *J. Comp. Chem.* **2015**, *37*, 506–541.

[22] B. O. Roos, R. Lindh, P. Å. Malmqvist, V. Veryazov, P.-O. Widmark, *Multiconfigurational Quantum Chemistry*, Wiley, Hoboken, NJ, USA, **2016**.

[23] B. O. Roos, in *Adv. Chem. Phys. Ab Initio Methods Quantum Chem. II, Vol. 69* (Ed.: K.P. Lawley), Wiley, New York, NY, USA, **1987**, pp. 399–455.

[24] P. Siegbahn, A. Heiberg, B. Roos, B. Levy, *Phys. Scr.* **1980**, *21*, 323–327.

[25] B. O. Roos, P. R. Taylor, P. E. M. Siegbahn, *Chem. Phys.* **1980**, *48*, 157–173.

[26] P. E. M. Siegbahn, J. Almlöf, A. Heiberg, B. O. Roos, *J. Chem. Phys.* **1981**, *74*, 2384–2396.

[27] T. Shiozaki, W. Gyroffy, P. Celani, H. J. Werner, *J. Chem. Phys.* **2011**, *135*, DOI 10.1063/1.3633329.

[28] B. O. Roos, P. Linse, P. E. M. Siegbahn, M. R. A. Blomberg, *Chem. Phys.* **1982**, *66*, 197–207.

[29] K. Andersson, P. A. Malmqvist, B. O. Roos, A. J. Sadlej, K. Wolinski, *J. Phys. Chem.* **1990**, *94*, 5483–5488.

[30] K. Andersson, P. Malmqvist, B. O. Roos, *J. Chem. Phys.* **1992**, *96*, 1218–1226.

[31] L. Ungur, L. F. Chibotaru, *Chem. Eur. J.* **2017**, *23*, 3708–3718.

[32] Y.-C. Chen, J.-L. Liu, Y. Lan, Z.-Q. Zhong, A. Mansikkamäki, L. Ungur, Q.-W. Li, J.-H. Jia, L. F. Chibotaru, J.-B. Han, et al., *Chem. – A Eur. J.* **2017**, *23*, 5708–5715.

[33] P. Å. Malmqvist, B. O. Roos, B. Schimmelpfennig, *Chem. Phys. Lett.* **2002**, *357*, 230–240.

[34] L. Ungur, M. Thewissen, J.-P. Costes, W. Wernsdorfer, L. F. Chibotaru, *Inorg. Chem.* **2013**, *52*, 6328–6337.

[35] L. F. Chibotaru, L. Ungur, *J. Chem. Phys.* **2012**, *137*, 64112.

[36] L. Ungur, L. F. Chibotaru, in *Lanthanides Actinides Mol. Magn.* (Eds.: R.A. Layfield, M. Murugesu), Wiley-VHC, Weinheim, Germany, **2015**, pp. 153–184.

[37] W. Kutzelnigg, W. Liu, *J. Chem. Phys.* **2005**, *123*, 241102.

[38] D. Peng, M. Reiher, *Theor. Chem. Acc.* **2012**, *131*, 1081.

[39] M. Filatov, *J. Chem. Phys.* **2006**, *125*, 107101.

[40] O. Christiansen, J. Gauss, B. Schimmelpfennig, *Phys. Chem. Chem. Phys.* **2000**, *2*, 965–971.

[41] B. A. Heß, C. M. Marian, U. Wahlgren, O. Gropen, *Chem. Phys. Lett.* **1996**, *251*, 365–371.
[42] P.-O. Widmark, P.-Å. Malmqvist, B. O. Roos, *Theor. Chim. Acta* **1990**, *77*, 291–306.
[43] B. O. Roos, R. Lindh, P.-Å. Malmqvist, V. Veryazov, and Per-Olof Widmark, *J. Phys. Chem. A* **2004**, *108*, 2851–2858.
[44] B. O. Roos, R. Lindh, P.-Å. Malmqvist, V. Veryazov, P.-O. Widmark, A. C. Borin, *J. Phys. Chem. A* **2008**, *112*, 11431–11435.
[45] K. W. H. Stevens, *Proc. Phys. Soc. Sect. A* **1952**, *65*, 209–215.
[46] N. Iwahara, L. Ungur, L. F. Chibotaru, *Phys. Rev. B* **2018**, *98*, 54436.
[47] N. Iwahara, L. F. Chibotaru, *Phys. Rev. B* **2015**, *91*, 174438.
[48] A. Lunghi, F. Totti, S. Sanvito, R. Sessoli, *Chem. Sci.* **2017**, *8*, 6051–6059.
